# Long term care facilities in England during the COVID-19 pandemic—a scoping review of guidelines, policy and recommendations

**DOI:** 10.1186/s12877-024-04867-9

**Published:** 2024-05-03

**Authors:** Danni Collingridge Moore, Alex Garner, Natalie Cotterell, Andrew J. E. Harding, Nancy Preston

**Affiliations:** 1 Division of Health Research, Lancaster University, Lancaster, UK; 2 Lancaster Medical School, Lancaster University, Lancaster, UK

**Keywords:** Coronavirus, COVID-19, long-term care facilities, Care homes, Nursing homes, Health policy, Public health

## Abstract

**Background:**

The disproportionate effect of COVID-19 on long term care facility (LTCF) residents has highlighted the need for clear, consistent guidance on the management of pandemics in such settings. As research exploring the experiences of LTCFs during the pandemic and the implications of mass hospital discharge, restricting staff movement, and limiting visitation from relatives are emerging, an in-depth review of policies, guidance and recommendations issued during this time could facilitate wider understanding in this area.

**Aims:**

To identify policies, guidance, and recommendations related to LTCF staff and residents, in England issued by the government during the COVID-19 pandemic, developing a timeline of key events and synthesizing the policy aims, recommendations, implementation and intended outcomes.

**Method:**

A scoping review of publicly available policy documents, guidance, and recommendations related to COVID-19 in LTCFs in England, identified using systematic searches of UK government websites. The main aims, recommendations, implementation and intended outcomes reported in included documents were extracted. Data was analysed using thematic synthesis following a three-stage approach: coding the text, grouping codes into descriptive themes, and development of analytical themes.

**Results:**

Thirty-three key policy documents were included in the review. Six areas of recommendations were identified: infection prevention and control, hospital discharge, testing and vaccination, staffing, visitation and continuing routine care. Seven areas of implementation were identified: funding, collaborative working, monitoring and data collection, reducing workload, decision making and leadership, training and technology, and communication.

**Discussion:**

LTCFs remain complex settings, and it is imperative that lessons are learned from the experiences during COVID-19 to ensure that future pandemics are managed appropriately. This review has synthesized the policies issued during this time, however, the extent to which such guidance was communicated to LTCFs, and subsequently implemented, in addition to being effective, requires further research. In particular, understanding the secondary effects of such policies and how they can be introduced within the existing challenges inherent to adult social care, need addressing.

**Supplementary Information:**

The online version contains supplementary material available at 10.1186/s12877-024-04867-9.

## Introduction

Across the majority of countries with ageing populations, the COVID-19 pandemic had a significant impact on older adults in long term care facilities (LTFCs) [1]. In England alone, during the first two months of the pandemic there were 12,526 deaths among LTCF residents either confirmed or suspected as related to COVID-19, with deaths in LTCFs increasing by 220% in the first ten weeks of the pandemic [2, 3]. In 2022, the LTFC population was nearly 8% lower than before the pandemic, falling from approximately 391,927 to 360,792, possibly due to widespread concern regarding the quality of care available in LTCFs [4]. Similar experiences occurred in Canada, Australia, and the United States [5–7]. In this paper, an LTCF is defined as a collective institutional setting where care is provided for the older people who live there, 24 h a day, seven days a week, for an undefined period, and can refer to care homes, nursing homes or residential aged care facilities [8, 9].

The timings of the COVID-19 pandemic varied by country, in terms of first cases, travel restrictions and lockdowns. In England, the first cases of COVID-19 were confirmed 31st January 2020, and the national response to the pandemic can loosely be described in four stages; the first national lockdown (23rd March 2020 to 13th May 2020), autumn/winter 2020 restrictions and the second national lockdown (14th October 2020 to 4th January 2021), the third national lockdown (5th January 2021 to 8th March) and Plan B (8th December – 27th January 2022).  (see Table 1).

**Table 1:** Timeline of issued guidance related to wider UK and international events (left column) and the management of COVID-19 in LTCFs in England (right column), from December 2019 to June 2022

First cases of COVID-19 in China reported [10]	31-Dec	Dec 19		
WHO declares COVID-19 a public health emergency of international concern [10]	30-Jan	Jan 20	30-Jan	NHS England declares a Level 4 National Incident [11]
First cases of COVID-19 in England are confirmed [12]	31-Jan			
		Feb 20	25-Feb	‘Guidance for social or community care and residential settings on COVID-19’^a^ [13]
First COVID-19 death in England is confirmed [14]	05-Mar	Mar 20	03-Mar	‘COVID-19 action plan’ published [15]
WHO defines COVID-19 as a pandemic [16]	11-Mar		16-Mar	CQC announce immediate cessation of routine inspections [17]
Social distancing measures announced [18]	16-Mar		17-Mar	'Next Steps on NHS Response to COVID-19'—freeing up inpatient and critical care capacity [11]
			19-Mar	‘COVID-19: hospital discharge service requirements’^a^ [19]
			19-Mar	‘Responding to COVID-19: the ethical framework for adult social care’^a^ [20]
First lockdown in England begins [21]	23-Mar		23-Mar	Appeal to recruitment agencies to work with social care providers [22]
			27-Mar	LGA/ADASS raise concerns regarding PPE provision for adult social care [23]
			29-Mar	COVID-19 testing to support retention of NHS staff [24]
			30-Mar	CQC release joint statement on advance care planning [25]
WHO guidance published on asymptomatic transmission [26]	02-Apr	Apr 20	02-Apr	‘COVID-19: admission and care of people in care homes’^a^ [27]
			04-Apr	‘COVID-19: management of staff and exposed patients and residents in health and social care settings’^a^ [28]
			09-Apr	**‘**Coronavirus (COVID-19): looking after people who lack mental capacity’^a^ [29]
			10-Apr	CQC requires care homes to report COVID-19 deaths [30]
			15-Apr	‘COVID-19: our action plan for adult social care’^a^ [31]
Lockdown extended for “at least” three weeks [32]	15-Apr		15-Apr	Deaths involving COVID-19 in care homes in England: transparency statement published [30]
			17-Apr	COVID-19: how to work safely in care homes^a^ [33]
			23-Apr	Adult social care recruitment care campaign launched [34]
			27-Apr	Death in service benefits for frontline NHS and social care staff [35]
			28-Apr	Daily briefing – “government will now publish data on deaths in care homes” [36]
			30-Apr	CQC launches Emergency Support Framework [37]
Conditional plan for lifting lockdown announced [38]	10-May	May 20	01-May	NHS sets out clinical service model for care home support [39]
			06-May	Dedicated app for social care workers launched [40]
			11-May	Government publishes 'Our Plan to Rebuild' [41]
			15-May	Coronavirus (COVID-19): support for care homes [42]
			19-May	Health and wellbeing of the adult social care workforce^a^ [43]
			20-May	Bereavement scheme extended to dependents of social care workers [44]
			21-May	Social care staff exempt from immigration health surcharge [44]
			26-May	‘Join Social Care' tool launched to speed up social care recruitment [45]
English primary schools encouraged to re-open [46]	01-Jun	Jun 20	06-Jun	NHS Volunteer Responders scheme extended to social care staff [47]
			07-Jun	Government meets its target to offer COVID-19 tests to every care home for over-65 s [48]
			08-Jun	Government announces Social Care Sector COVID-19 support taskforce [49]
Non-essential retail re-opened [50]	15-Jun		09-Jun	‘About the Adult Social Care Infection Control Fund’^a^ [51]
The first local lockdown is introduced in Leicester [52]	29-Jun		19-Jun	“Coronavirus (COVID-19): reducing risk in adult social care”^**a**^ [53]
Restrictions are eased in England [54]	04-Jul	Jul 20	03-Jul	Repeat testing strategy for LTCF staff (weekly) and residents (every 4 weeks) [55]
			17-Jul	'The next chapter in our plan to rebuild’ [56]
WHO issues a policy brief to prevent and mitigate the impact of COVID-19 across all aspects of long-term care [57]	24-Jul		20-Jul	‘COVID-19 supplement to the IPC resource for adult social care’ [58]
			22-Jul	‘Visiting arrangements in care homes’^a^ [59]
			31-Jul	‘Personal protective equipment: illustrated guide for community and social care settings’^a^ [60]
		Aug 20	25-Aug	Overview of adult social care guidance on coronavirus (COVID-19)’^a^ [61]
Social gatherings above six banned in England [62]	14-Sep	Sep 20	11-Sep	Letter to social care providers highlighting the importance of testing and PPE [63]
			18-Sep	‘Adult social care: our COVID-19 winter plan 2020 to 2021’^a^ [64]
Pubs and restaurants in England to close at 22:00 [65]	24-Sep		18-Sep	Government publishes the Social Care Sector COVID-19 Support Taskforce’s report on first phase of COVID-19 pandemic [66]
Three-tier system of restrictions begins in England [67]	14-Oct	Oct 20	01-Oct	‘Adult Social Care Infection Control and Testing Fund: round 2’^a^ [68]
			06-Oct	CQC sets out its transitional regulatory approach [69]
			13-Oct	‘Winter Discharges—Designated Setting’ [70]
Second lockdown in England begins [71]	05-Nov	Nov 20	23-Nov	'COVID-19 Winter Plan' [72]
			27-Nov	PHE publishes COVID-19 vaccination programme [73]
Second lockdown ends, returns to three tier system [74]	02-Dec	Dec 20	01-Dec	CQC publish information on regulating 'designated care settings' [75]
			01-Dec	Government rolls out lateral flow testing to enable indoor visiting in all LTCFs [76]
Regulatory approval of Pfizer/BioNTech vaccine [77]	02-Dec		04-Dec	Vaccinations in LTCFs programme launched [78]
COVID-19 vaccination delivered in England [79]	08-Dec		14-Dec	'COVID-19: our action plan for adult social care' – updated [31]
			16-Dec	‘Discharge into care homes: designated settings’^a^ [80]
Fourth tier of restrictions introduced in England [81]	19-Dec		20-Dec	NHS issue guidance on staffing to support vaccination in LTCFs [82]
England enters third national lockdown [83]	06-Jan	Jan 21	11-Jan	‘UK COVID-19 vaccines delivery plan’ [84]
Moderna vaccine approved [85]	08-Jan		15-Jan	‘Adult Social Care Rapid Testing Fund: guidance’^a^ [86]
			17-Jan	Social care sector to receive £269 million boost staffing and testing [87]
AstraZeneca/Oxford vaccine approved [88]	30-Jan		22-Jan	‘Your care home during winter’^a^ [89]
			29-Jan	Workforce Capacity Fund for adult social care [90]
Roadmap to ease lockdown restrictions announced [91]	22-Feb	Feb 21	01-Feb	Every older LTCF resident in England offered a COVID-19 vaccine [92]
			09-Feb	‘Care for Others. Make a Difference’ recruitment campaign launched [93]
			25-Feb	COVID-19 vaccine: one of UK's largest LTCF firms introduces 'no jab, no job' policy [94]
Step 1 of lockdown easing begins in England [95]	08-Mar	Mar 21	01-Mar	‘Restricting workforce movement between care homes and other care settings’^a^ [96]
Gatherings of six people allowed in England [91]	29-Mar		24-Mar	‘Coronavirus (COVID-19) testing available for adult social care in England’^a^ [97]
			29-Mar	‘Adult Social Care Infection Control and Testing Fund’^a^ [98]
Twice weekly rapid testing available in England [99]	09-Apr	Apr 21	12-Apr	LTCF residents in England allowed two visitors [100]
Step 2 of lockdown easing begins in England [91]	12-Apr		14-Apr	Consultation launched on COVID-19 vaccines among LTCF staff [101]
Further easing of COVID-19 restrictions announced [91]	17-May	May 21	04-May	LTCF residents can go on outdoor trips without isolating [102]
			17-May	LTCF residents allowed five named visitors [103]
Janssen vaccine approved [104]	28-May		20-May	‘Testing for professionals visiting care homes’^a^ [105]
		Jun 21	16-Jun	LTCF staff to be fully vaccinated under new law, to be implemented in October 2021 [106]
Further easing of COVID-19 restrictions announced [107]	19-Jul	Jul 21	02-Jul	‘Adult social care extension to Infection Control and Testing Fund 2021’^a^ [108]
			19-Jul	Frontline health and care staff can work rather than self-isolate [109]
Self-isolation removed for double-jabbed contacts	16-Aug	Aug 21	04-Aug	‘Coronavirus (COVID-19) vaccination of people working or deployed in care homes: operational guidance’^a^ [110]
		Sep 21	07-Sep	Record £36 billion investment to reform NHS and Social Care [111]
			14-Sep	JCVI issues updated advice on COVID-19 booster vaccination [112]
			15-Sep	Temporary medical exemptions for COVID-19 vaccination of LTCF staff [113]
		Oct 21	21-Oct	‘Adult Social Care Infection Control and Testing Fund: round 3’^a^ [114]
			29-Oct	Guidance updated to allow flexibility in booster programme for LTCF residents [115]
		Nov 21	03-Nov	Workforce Recruitment and Retention Fund^a^ [116]
			11-Nov	COVID-19 vaccination introduced as a condition of deployment for all frontline social care workers [117, 118]
			24-Nov	Lift COVID-19 ban on staff working in more than one LTCF [96]
Plan B implemented in England [119]	08-Dec	Dec 21	10-Dec	Support package to protect care sector this winter [120]
			10-Dec	‘People at the Heart of Care: adult social care reform’ [121]
Self-isolation for COVID-19 cases reduced from 10 to 7 days following negative LFD tests [122]	22-Dec		16-Dec	Workforce Recruitment and Retention Fund for adult social care, round 2^a^ [123]
			24-Dec	Health and Care Visa scheme expanded [124]
Positive LFT no longer required to take PCR test [125]	11-Jan	Jan 22	10-Jan	Adult Social Care Omicron Support Fund [126]
			13-Jan	Free PPE for frontline extended for another year [127]
Self-isolation can end after 5 days following 2 negative LFD tests [128]	17-Jan		27-Jan	Government eases social care restrictions after booster success, including unlimited visitors [129]
England to return to Plan A [130]	19-Jan		31-Jan	Consultation on removing vaccination as a condition of employment for social care staff announced [131]
Plan for living with COVID-19 announced [132]	24-Feb	Feb 22		
UK COVID-19 inquiry draft terms of reference set out [133]	11-Mar	Mar 22	01-Mar	Regulations making COVID-19 vaccination a condition of deployment to end [134]
			03-Mar	‘A guide to the spring booster for those aged 75 years and older and older residents in care homes’^a^ [135]
			31-Mar	‘Infection prevention and control in adult social care: COVID-19 supplement’^a^ [136]
Mass free testing stops [137]	01-Apr	Apr 22	05-Apr	‘Bereavement resources for the social care workforce’^a^ [138]
			06-Apr	Health and Social Care Levy to raise billions for NHS and social care [139]
		May 22	19-May	JCVI provides interim advice on an autumn COVID-19 booster programme [140]

Events marked with an ^a^refer to guidance documents included in the review

Acronyms: *ADASS* Association of Directors of Adult Social Services, *CQC* Care Quality Commission, *IPC* Infection Prevention and Control, *JCVI* Joint Committee on Vaccination and Immunisation, *LFD* Lateral flow device, *LGA* Local Government Association, *NHS* National Health Service, *PCR* Polymerase chain reaction, *PHE* Public Health England, *PPE* Personal protective equipment, *WHO* World Health Organisation

In hindsight, the likely effect of the COVID-19 pandemic on a relatively vulnerable LTCF population could have been predicted. Compared to older adults living privately in the community, LTCF residents are more likely to be frail, have existing comorbidities including dementia or some form of cognitive impairment, with an average age of over 80 years [141, 142]. Such characteristics are now associated with being more susceptible to, and to subsequently die from, COVID-19 infection [143]. In addition, contact between residents and staff, both in private rooms and in communal areas, is frequent, making isolating, segregating, or shielding residents and staff problematic [144]. The COVID-19 pandemic created additional burdens to maintaining an LTCF workforce, which was already characterised by comparatively low pay, high staff turnover, and limited opportunities for training, support and development which are largely dependent on the leadership and management of individual LTCFs [145].

Developing national policies to manage COVID-19 across such settings is challenging. During the pandemic, LTCFs reported difficulties in accessing and using personal protective equipment (PPE), managing COVID-19 related staff absences and the impact of the pandemic on health and wellbeing, and in reducing the use of agency staff across multiple sites [146–150].

Like most high and middle income countries, the UK government published national guidelines to tackle the spread of COVID-19 in LTCFs in England, including hastening hospital discharge, restricting visitation from family and friends and promoting remote primary care, among others [19, 31, 59]. At present, there has been no systematic synthesis of these policies, or of their aims, implementation or intended outcomes, within the wider context of the pandemic. Given the growing likelihood of future pandemics, and ongoing criticism at how the pandemic response was managed in LTCFs in England, reflecting on UK policies is imperative to understand why LTCFs were affected as they were, and how LTCFs can be managed during pandemics in the future both in the UK and internationally [151].

## Aims and objectives

Publicly available policy, guidance and recommendations from the UK government related to LTCFs in England during the COVID-19 pandemic are explored in this scoping review. Firstly, it aims to provide a timeline of key events related to LTCFs, and secondly it aims to synthesise the aims, recommendations, implementation, and intended outcomes of the guidance identified.

## Methods

A scoping review approach, as developed by Arksey and O'Malley, was used to synthesise key policy documents [152]. The five-stage approach included; identifying the research question, identifying relevant studies, study selection, charting the data and collating, summarizing and reporting the results [152].

### Identifying the research question

Firstly, the primary research question was discussed and refined, and the two review questions were identified.

### Identifying relevant studies

The UK government website was searched for policy, guidance and recommendations, using the key terms "COVID-19", "Coronavirus", "care home" and "adult social care", in October 2022 [153]. The results included policy or strategy documents, white papers, guidance or working papers. Included documents could either be published papers or online webpages. The approach was informed by recommended review methods for grey literature, and a snowballing strategy was used to identify sources referenced or linked to within included documents [154, 155]. Where required, an internet archive resource was used to access the original publications if no longer available online [156].

### Study selection

Guidance documents were included if they met the inclusion criteria shown in Table 2. In stage one, titles and executive summaries were reviewed for potential inclusion, and if suitable a full document was sourced for review in stage two. A randomly selected subset of 20% of the documents were reviewed by a second reviewer (AG).

**Table 2:** Inclusion and exclusion criteria

**Inclusion criteria**	**Exclusion criteria**
• Guidance, recommendations, or policy issued by a UK national governing body, relating to England • Guidance related predominantly to the management of COVID-19 or providing care during the COVID-19 pandemic either in LTCFs for older adults, or among older adult LTCF residents. Guidance related to adult social care, which does not specifically exclude LTCFs for older adults, would be included • An LTCF is operationally defined as a long-term care setting where several older people live, with access to on-site care services. It may be either CQC or non CQC registered • Guidance related to LTCFs in England • Published online between 1st January 2020 and 1st June 2022 • Original publication accessible online as of October 2022	• Guidance, recommendations, or policy issued by non-government bodies • Documentation reporting data only, press releases or that contained no guidance, recommendations, or policy • Content unrelated to the management of COVID-19 either in LTCFs, or among LTCF residents, such as infection control in general, or managing COVID-19 in the community, acute settings, sheltered accommodation etc. Guidance related to LTCFs for any group other than older adults, such as children or those with learning disabilities, were excluded • Guidance related to Northern Ireland, Scotland and Wales, or countries outside of the United Kingdom • Guidance related to a specific region or locality within England, such as local government guidance

### Charting the data

Five areas of data were extracted; publication data, including author, date and central theme; the stated aim of the guidance, the main recommendations, implementation of the recommendation, and intended outcome, where stated. A randomly selected subset of 20% of the included documents were discussed with a second reviewer (AG), who independently checked the data extracted. If disagreements arose between reviewers, these were discussed openly and if necessary, a third reviewer was included to make a final decision (NP). At this stage, included documents were added to a narrative timeline of key international, national and LTCF related events.

### Collating, summarizing and reporting of results

Finally, the extracted data was analysed using thematic synthesis, starting with coding the text, grouping the codes into descriptive themes, and developing analytical themes (DCM/AG) [157]. Thematic synthesis was used as the approach transparently connects the data collected to the conclusions interpreted from the analytical themes [157].

The analytical themes generated were then discussed by the research team, re-applied to the data, and subsequently refined, using NVivo v12 [158]. The Enhancing Transparency in Reporting the synthesis of Qualitative research (ENTREQ) statement and the Preferred Reporting Items for Systematic Reviews and Meta-Analyses Extension for Scoping Reviews (PRISMA-ScR) were used to direct the reporting of the review [159, 160].

## Results

**Fig. 1:**
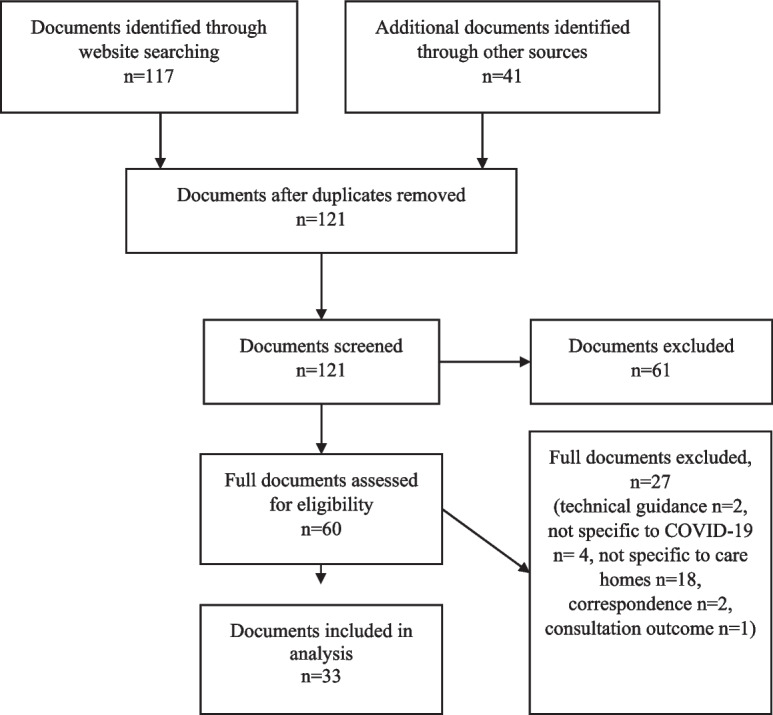
PRISMA flowchart

The screening process is shown in Fig. 1 and resulted in 33 included documents. The earliest guidance was from 25 February 2020, with the most recent guidance published 19 May 2022. Twenty-five documents were published by the Department of Health and Social Care, five by Public Health England/UK Health Security Agency and three were multi-authored, including by the Care Quality Commission (CQC) and NHS England.

The 33 documents focused on ten areas: nine focused on available funding to support LTCFs, seven provided multi-thematic guidance, such as outbreak management and controlling the spread of infection, five focused on COVID-19 testing or vaccination, and two on discharge and admission, caring for residents or staff with COVID-19, providing equitable care for residents, supporting the workforce, working safely in LTCFs and visiting/movement between LTCFs, respectively. Full data extracted is shown in the supplementary material. A timeline of key international and national events related to LTCFs in England during the pandemic is shown in Table 1, with the main funding streams shown in Table 3.

**Table 3:** Main funding streams in England for either adult social care or discharge to adult social care during the COVID-19 pandemic

**Funding title**	**Announced**	**Amount**	**Description**
Hospital Discharge Funding	Mar 2020	£1.3 billion	To support NHS and local authorities to work together to fund the additional needs of people leaving hospital during the pandemic
Adult Social Care Infection Control Fund	May 2020	£600 million	To support adult social care providers to reduce the rate of COVID-19 transmission in and between care homes and support wider workforce resilience
Adult Social Care Infection Control Fund: round 2	Oct 2020	£546 million	Same as the Adult Social Care Infection Control Fund
Adult Social Care Rapid Testing Fund	Jan 2021	£149 million	To support increased LFD testing in care settings
Workforce Capacity Fund for adult social care	Jan 2021	£120 million	To enable local authorities to deliver measures to supplement and strengthen adult social care staff capacity to ensure that safe and continuous care is achieved
Adult Social Care Infection Control and Testing Fund	Mar 2021	£341 million	Consolidates the Adult Social Care Infection Control Fund and the Adult Social Care Rapid Testing Fund
Adult Social Care Infection Control and Testing Fund—extension	Jul 2021	£250 million	Extension, consolidates the Adult Social Care Infection Control Fund and the Adult Social Care Rapid Testing Fund
Adult Social Care Infection Control and Testing Fund: round 3	Oct 2021	£388 million	Same as the Adult Social Care Infection Control and Testing Fund, including vaccine funding
Workforce Recruitment and Retention Fund	Nov 2021	£162.5 million	To support local authorities to address adult social care workforce capacity pressures through recruitment and retention activity
Workforce Recruitment and Retention Fund for adult social care, round 2	Dec 2021	£300 million	Same as the Workforce Recruitment and Retention Fund
Adult Social Care Omicron Support Fund	Jan 2022	£60 million	To support the sector with measures already covered by the infection prevention and control allocation of the Infection Control and Testing Fund (round 3) to reduce the rate of COVID-19 transmission within and between care settings through effective IPC practices

### Timeline of LTCFs in England during the COVID-19 pandemic

#### First national lockdown (23rd March 2020 to 13th May 2020)

The first publicly issued guidance for LTCFs was published on the 25th February 2020, more than three weeks after the first cases of COVID-19 in England were confirmed. By this time, the National Health Service (NHS) England had declared the pandemic a Level 4 National Incident, implementing a nationally coordinated response [11, 12, 161]. In hindsight, the recommendations underestimated the potential impact of COVID-19 in residential settings; despite recognition that older adults were likely to experience more severe COVID-19 symptoms, the guidance stated that it remained unlikely that those receiving care in a LTCF would become infected [161]. On the 11th March 2020, COVID-19 was defined as a pandemic, and national social distancing measures were announced [16, 18]. The ‘COVID-19 Action Plan’ was published, with minimal reference to adult social care, as the CQC, the independent regulator of health and social care in England. announced the immediate cessation of routine inspections [15, 17].

On 17th March 2020, the ‘Next steps on NHS response to COVID-19’ was published by NHS England, which aimed to free up hospital beds through postponing all non-urgent elective operations, and urgently discharging hospital inpatients as soon as it was clinically safe to do so, anticipating 1% of which would be discharged to LTCFs [11, 19]. The only reference to testing for COVID-19 was that, where applicable, test results would be included within patient discharge documentation [19]. By 2nd April 2020, guidance reiterated that negative tests were not required prior to LTCF admission, with family and friends advised not to visit LTCFs except next of kin in exceptional situations such as end of life [27]. As England entered its first lockdown, the implications of the pandemic on adult social care, in terms of staff absences, access to PPE and concerns regarding the blanket application of advance care plans at end of life, were being recognized within government briefings [21–23, 25].

National lockdown was extended for three weeks, and on 15th April 2020, ‘COVID-19: our action plan for adult social care’ was published, adopting a four-pillar approach based on (a) controlling the spread of infection, (b) supporting the workforce, (c) supporting independence, people at the end of life, and responding to individual needs, and (d) supporting local authorities and the providers of care [31, 32]. The action plan committed to an emergency release of seven million PPE items, alongside a further 34 million items of PPE across local resilience forums (LRFs); local multi-agency partnerships made up of representatives from local public services. The action plan initiated testing for social care workers and their households, in line with NHS staff workforce, introduced an online reporting system for LTCFs (the ‘Capacity Tracker’), implemented COVID-19 testing prior to LTCF admission, reiterated a commitment to providing appropriate end of life care to residents, and addressed increasing the social care workforce [24]. A recruitment campaign for adult social care commenced, and the CQC launched the ‘Emergency Support Framework’, to identify care providers that needed extra support to respond to the pandemic [34, 37].

As lockdown restrictions began to lift in May 2020, the COVID-19 recovery strategy ‘Our Plan To Rebuild’ re-emphasised the importance of protecting LTCFs, committing to the testing of all symptomatic LTCF residents and hospital patients discharged into LTCFs [38, 41]. It offered a polymerase chain reaction (PCR) COVID-19 test to every staff member and resident, symptomatic or asymptomatic, in LTCFs in England, by 6th June 2020. Further recommendations on reducing workforce movement between LTCFs were published, acknowledging significant asymptomatic transmission in LTCFs via both residents and staff [42]. The recommendations were supported by a £600 million Infection Control Fund [51].

#### Autumn and winter 2020 restrictions and second national lockdown (14 October 2020 to 4 January 2021)

On the 8th June 2020, it was confirmed that the testing target to distribute ‘whole home’ testing kits, for all residents and staff within any LTCF for residents over 65 or those with dementia, had been met, and by the 6th July 2020, weekly staff and monthly resident testing for all LTCFs had been implemented [55]. By mid- September, the first local lockdown was introduced in Leicester, and limitations on groups of more than six were introduced in England to curb the growing number of infections, including an increase among LTCF staff [62, 63]. These recommendations were timely, as on 24th September 2020 further restrictions were announced, pre-empting local lockdowns, prior to national lockdown on 5th November 2020 [65, 67, 71, 74, 81]. Further funding for adult social care was announced, and the CQC introduced ‘designated settings’, areas within LTCFs where newly admitted residents with COVID-19 could safely complete a period of isolation [64, 68, 80].

#### Third national lockdown (from 6 January 2021 to 8^th^ March)

England’s third national lockdown was introduced on 6th January 2021, as new variants of COVID-19 emerged and the vaccination programme was being rolled out [83, 84, 89]. LTCF residents and staff were prioritized for vaccination by the Joint Committee on Vaccination and Immunisation, and by 1st February 2021 every LTCF resident over 65 years had been offered a COVID-19 vaccine [73, 92]. By April 2021, free lateral flow device (LFD) tests were provided to everyone in England, which further supported visitation to LTCFs; and from 12th April 2021, LTCF residents were allowed two regular visitors, indoors, on the condition of providing a negative LFD test [99, 100, 105]. As further easing of COVID-19 restrictions were announced double-vaccinated staff were permitted to attend work instead of self-isolating, contingent on a negative PCR test and daily negative LFD tests [107, 109]. By September 2021, LTCF residents and staff were offered a COVID-19 booster vaccine [112, 115].

As LTCF staff became eligible for vaccination, some care providers introduced a 'no jab, no job' policy, which was widely criticised at a time of staff shortages [94]. In June 2021 it was announced that anyone working in an LTCF required two doses of a COVID-19 vaccine, unless medically exempt [101, 106, 110, 113]. In November 2021, COVID-19 vaccination became a condition of employment for all frontline social care workers, and restrictions on staff working in multiple settings were lifted to tackle staff shortages [117, 118].

#### Plan B (8 December—27 January 2022) and ending of restrictions

In December 2021, as COVID-19 infections again increased, England temporarily moved to ‘Plan B’, which recommended working from home and face coverings in public indoor venues [119]. These restrictions were subsequently removed on the 27th January 2022, which was said to be possible due to the success of the booster vaccination programme [130]. On 1st March 2022, regulations making vaccines a condition of deployment for social care staff were revoked, after widespread criticism [134]. In February 2022, the government’s plan for removing the remaining legal restrictions was published, including removing the need for self-isolation after a positive test, and discontinuation of mass free testing on 1st April 2022 [132, 137]. LTCF residents and staff would still be able to access free symptomatic/ asymptomatic testing, with residents offered a second, spring booster, and a third booster in Autumn 2022 [135, 140].

### Thematic analysis

The aims, main recommendations, implementation and outcomes of the documents were analysed using thematic analysis. Six areas of recommendations were identified: infection prevention and control, hospital discharge, testing , staffing, visitation and continuing routine care. In addition, seven areas of implementation were identified: funding, collaborative working, monitoring and data collection, reducing workload, decision making and leadership, training and technology and communication.

## Aims and intended outcomes of the included documents

Where stated, the guidance predominantly aimed to reduce the risk of, or help prevent and control, COVID-19 transmission in LTCFs, and prevent future outbreaks, while ensuring that residents continued to receive appropriate care. As the pandemic progressed, this focus shifted to supporting care providers to reduce the rate of COVID-19 transmission in, and between, LTCFs. While the guidance consistently focused on protecting residents and staff, over time the additional need to protect vulnerable staff from COVID-19 infection was recognised. Specific aims related to hospital discharge service requirements, providing effective infection prevention and control (IPC) practices, supporting workforce resilience, capacity and health and wellbeing, reducing movement between sites, enabling visiting, and increasing testing and vaccination uptake. Seasonal guidance, such as providing care in LTCFs during winter, was also published [64, 89].

In terms of implementation, the policies aimed to disseminate guidance across services, including to local authorities, NHS organisations and care providers, at local, regional and national level. In some cases, the guidance required further dissemination, such as asking care providers to pass on advice to their staff, or to support health professionals in developing facility specific policies in line with their own professional codes of conduct and regulations [20, 29].

Overall, the intended outcomes, where explicitly stated in the guidance, reflected the aims, and focused on reducing the risk of transmitting the infection to others and avoiding exposure to COVID-19. Specific outcomes included preventing and controlling COVID-19, protecting staff, reducing the rate of transmission in and between LTCFs, increasing uptake of staff vaccination and providing funding to support these outcomes. In some cases, outcomes centred on supporting decision makers, such as in conducting risk assessments [53].

## Main recommendations of the included documents

Six themes relating to recommendations were identified: infection prevention and control, hospital discharge, testing, staffing, visitation and continuing routine care. The guidance was updated throughout the pandemic, as the rate of transmission varied, and local lockdowns were introduced.

### Infection prevention and control

In the early days of the pandemic, initial guidance focused on the management of those exposed to COVID-19, and was limited to residents, visitors or staff who had visited specific countries. Normal practice was recommended for LTCF staff who had come into contact with COVID-19 without PPE, on the basis that exposure would be short-lived, and LTCF closures were not required [28, 161]. In addition, if a resident or staff member was asymptomatic, no change to care was required [161]. Within days, IPC guidance was updated, with emphasis on keeping asymptomatic residents safe through daily symptom monitoring and social distancing measures amongst residents and staff [27]. General PPE use was recommended for providing personal care, regardless of whether the resident had symptoms or was known to have COVID-19, recognising that older residents often had minimal symptoms of infection [33, 60].

Arguably the central strategy to minimising COVID-19 transmission in LTCFs, and nationally, was social distancing. Care providers were advised to follow social distancing measures for everyone within the facility, with extremely vulnerable groups subject to additional shielding [27]. This included reducing contact between staff, holding team meetings and handovers remotely, staggering times of entry to collect equipment, reducing communal activities, and having a smaller number of workers dedicated to supporting residents with COVID-19 [61, 136]. Any resident showing COVID-19 symptoms was to be isolated and separated immediately in a single room, with a separate bathroom, and isolation, ‘cohorting’ and infection control measures strictly implemented [27]. Cohorting referred to limiting residents and staff to floors or wings, segregating COVID-19-positive and COVID-19-negative residents [61]. Cohorting and zoning recommendations were published, and included early discussions with care providers regarding the safety and feasibility of implementing these arrangements within LTCFs [64]. In Dec 2021, the CQC released guidance on ‘designated settings’, areas within a LTCF that had additional policies, procedures, equipment, staffing and training in place to maintain infection control to safely care for COVID-19 positive residents admitted to the LTCF [80]. Funding to support social distancing was provided, and to pay for the costs associated with implementing cohorting, recruiting and paying extra staff, paying for structural or physical changes to support cohorting, and providing accommodation for staff who proactively chose to live in the facility, therefore reducing social contact outside work [51, 96, 98, 108, 114].

Two recommendations require further exploration: staff isolation and restricting staff movement.

Guidance on staff isolation was relatively consistent across the pandemic; staff with COVID-19 symptoms were asked to notify their line manager immediately and self-isolate for seven days, later extended to 10 days [27, 28, 61]. This included staff with a symptomatic or COVID-19 positive household member, or those notified to isolate by the NHS Test and Trace system, with funding available to reimburse the wages of self-isolating staff [64, 68].

In terms of staff movement, care providers were recommended to limit all staff movement between settings unless necessary. This applied to staff working for one care provider across several facilities, staff working on a part-time basis for multiple employers in multiple facilities, and agency staff [42, 68]. Where the use of agency staff was needed, care providers were asked to use block bookings, review exclusivity arrangements with recruitment agencies and recruit additional staff over winter [42, 68]. A ten day interval between staff attending the two settings and a negative test result prior to entering the facility was also recommended [96]. Again, the Infection Control Fund could be used to meet associated costs [64].

In response to concerns over access to PPE, an emergency provision of seven million items of PPE was provided, alongside 23 million items of PPE for onward sale to social care providers and the release of a further 34 million items of PPE across LRFs [31, 42]. Three emergency routes to access PPE were developed, an online PPE Portal, LRFs and the National Supply Disruption Response system, which responded to emergency PPE requests, supported by a 24/7 helpline and an express freight service [31]. Ongoing monitoring was provided through the Capacity Tracker, which collected key adult social care data, collating daily information on bed capacity, workforce absences, PPE levels, and overall risks in LTCFs, and a CQC community care survey [61, 64]. Maintaining PPE stocks was a consistent message throughout the guidance, especially during winter, and was sustained by IPC funding [68, 89, 98, 108, 114, 136]. Support and training for LTCF staff on implementing IPC was provided through training videos on using PPE, support from infection control nurses, identification of a lead individual for IPC within the facility to ensure adherence to infection prevention guidance, and undertaking post reflective learning reviews [31, 33, 42, 60, 64].

### Hospital discharge

Discharge from hospital for patients as soon as it was clinically safe to do so was implemented early on in the management of COVID-19 in England [19]. In practice, this meant that at the beginning of the pandemic older adults were discharged to LTCFs, without the requirement for a negative COVID-19 test prior to admission [27]. New residents required isolating for a 14-day period following admission, regardless of COVID-19 status. The guidance emphasised that no care provider would be forced to admit a resident if they were unable to safely cohort or isolate COVID-19 positive residents, with the responsibility on local authorities to provide alternative accommodation to quarantine and isolate residents [61].

This policy was later amended to testing all residents 48 h prior to discharge, with results communicated to the LTCF provider in advance and included in discharge documentation prior to admission [64, 80, 136]. Again, this was monitored by local health protection teams and through the Capacity Tracker [64, 136].

### Testing

Available, accessible COVID-19 testing was integral to the policy response for LTCFs. For two or more possible cases of COVID-19, testing to confirm an outbreak was arranged through health protection teams, who arranged for swabbing for up to five initial possible cases, with testing of all cases not required as it would not change subsequent outbreak management [27]. In addition to testing, local health protection teams provided advice on and supported outbreak management, including on isolating cases and reinforcing infection control practices, such as PPE use, appropriate staffing, and restricting visitation [31].

By July 2020, this approach had changed to testing all symptomatic residents, with the introduction of ‘whole home’, repeat testing for all residents implemented in July 2020 [31, 61]. Repeat testing included weekly PCR testing of staff and testing of residents every 28 days in LTCFs without outbreaks, with access testing for all their residents and staff via a digital portal [97]. Initially, testing was available for LTCFs with a new outbreak, COVID-19 free LTCFs with over 50 beds and LTCFs referred by local authorities, before extending to LTCFs for over-65s and those with dementia [42, 61].

The funding included the costs of PCR testing, ensuring that staff who needed to attend work or another location for the purposes of being tested for COVID-19 were paid their usual wages, as were any costs associated with travel to a testing facility, and any reasonable administrative costs associated with organising and recording outcomes of COVID-19 tests [89, 108, 114]. Testing was available for LTCF staff and their households through local test centres, in line with NHS staff, albeit introduced at a later date, and also for visiting health professionals and relatives [31, 97, 105].Testing was supported by multiple funding steams, which could be used to pay for staff costs associated with training and conducting LFT testing within the LTCF 86, 98.

### Staffing

Multiple policies focused on supporting adult social care staff during the COVID-19 pandemic. Firstly, staff were able to receive normal wages while self-isolating, funded through the Infection Control Fund [64, 98]. In addition, support was available in the form of increases to statutory sick pay, universal and working tax credit and the furlough scheme, whereby staff unable to work due to the pandemic were continued to be paid wages through a combination of government and employer contributions. [31, 117]. The Workforce Capacity Fund provided funding to address staff shortages, support restricted staff movement and to allow care providers to access additional staffing resources to minimise deployment of those who work in multiple settings [90]. In addition to self-isolating, staff classed as clinically vulnerable could be removed from providing direct care to symptomatic residents, with risk assessments encouraged to identify and protect potentially vulnerable workers [27, 53, 64].

Secondly, the guidance addressed recruiting and retaining staff through the launch of a national recruitment campaign to attract people to the social care workforce. Temporary arrangements to provide free, fast safety vetting checks to aid recruitment were introduced, including access to rapid online induction training for new staff, and the redeployment of existing staff into new roles [31, 90]. In addition, existing benefits NHS staff were made available to adult social care workers, including death in service benefits, designation as key workers and the establishment of the ‘CARE’ brand, a logo to recognise and identify the adult social care sector [31, 34, 35, 40, 45].

Thirdly, further support on managing health and wellbeing among the adult social care workforce was provided, including the extension of a crisis text messaging support service and a dedicated free-to-caller support helpline.

### Visitation

Restrictions on visitation from relatives were a point of contention throughout the pandemic. Despite recognition that restricting contact with relatives would likely have a detrimental effect on residents, for the majority of the pandemic family and friends were advised not to visit LTCFs, except in exceptional situations such as at the end of life [27, 110]. Specific guidance was issued for visitors, including limiting visitors to one at a time, minimising contact with other residents and staff, enabling a booking system for visitors, keeping personal interaction with the resident to a minimum and limiting visits to one room [27, 59]. Visiting policies were largely facility specific, with visiting restrictions rapidly imposed in the event of an outbreak, if local incidence rates increased or if the LTCF was located in an 'area of intervention’ [64]. As the rate of COVID-19 transmission reduced, limited visits were allowed, ensuring every resident was enabled to continue to receive one visitor [136].

The guidance provided advice for LTCFs on developing visiting policies, emphasising the need to provide regular, personalised updates on residents and the active involvement of the resident and their family or friends in making decisions regarding visitation [59]. Funding could be used to support safe visiting, including assigning staff to support and facilitate visits, putting in place additional IPC measures between visits, and alterations to the LTCF to allow safe visiting such as developing a dedicated space [68, 98, 108]. The guidance also supported alternative options to maintain social contact for residents during times of limited visitation, including the use of telephones or video calling [27].

### Continuing routine care

Finally, guidance focused on maintaining routine care for LTCF residents. Early on in the pandemic, LTCF managers were required to postpone routine, non-essential appointments, including those that would involve residents visiting a hospital or other health care facilities [27]. Care providers were asked to work with NHS partners to reduce unnecessary emergency admissions, by assessing the appropriateness of hospitalisation, consulting a resident's advance or emergency care plan and through discussions with the resident and their relatives to determine if hospitalisation was the best course of action [64].

Continuing care within LTCFs was supported by primary care networks, who were responsible for delivering the ‘Enhanced Health in Care Homes’ framework, which provided access to clinical advice for staff and residents, including a named clinical lead and weekly multidisciplinary team support [31]. The guidance emphasised that if medical advice on routine care was needed, LTCFs should consider telemedicine consultations, delivered remotely via a phone call or video conferencing, alongside virtual rounds and multidisciplinary team meetings, unless a physical presence was clinically required [27, 31, 39, 42]. An accelerated rollout of cross-service e-mail and conferencing software was delivered to LTCFs to enhance communication with healthcare providers, allowing secure sharing of information between services [31, 61].

Early in the pandemic, concerns regarding anecdotal reports of blanket application of advance care plans led to a joint statement issued from the CQC, British Medical Association, Care Provider Alliance and Royal College of General Practitioners reiterating that any advance care plan, including Do Not Attempt Cardiopulmonary Resuscitation orders, should be person centred and made on an individual basis [25]. In particular, any advance care decision should be fully discussed with the resident and their family, and signed by the clinician responsible for their care [61, 64]. Further guidance was issued on end of life care in the context of the mental capacity act, and removing the requirement for family testing for residents at end-of-life [29, 64, 136].

## Implementation of the included documents

Seven areas of recommendations were identified: funding, collaborative working, monitoring and data collection, reducing workload, decision making and leadership, training and technology and communication. These are expanded on below.

### Funding

Throughout the pandemic, multiple funding streams were established to support hospital discharge, infection prevention and control, workforce capacity and later testing and vaccination, as shown in Table 3. The specific aims of the funding have been referenced in the main recommendation’s discussion. In most cases, funding was provided to local authorities, who were able to pass on approximately 80% of this funding to LTCFs, with the rest of the funding allocated at the discretion of the local authority  [68, 86].

### Collaborative working

The need for collaborative working across services was repeatedly emphasised, specifically between the NHS and care providers. This included recommendations for timely access to clinical advice, including a named clinical lead with weekly check-ins, proactive support for residents through personalised care and support, support for residents with suspected or confirmed COVID-19 through remote monitoring, and sensitive and collaborative decisions around hospital admissions for residents [42]. Outside the NHS, local resilience forums were responsible for managing the local response to the pandemic, in addition to wider stakeholders, such as Care Provider Associations [31, 59, 161].

### Monitoring and data collection

Monitoring systems were developed, namely the Capacity Tracker, which provided intelligence on adult social care for decision making [19, 31]. In addition, suspected and confirmed COVID-19 deaths were reported by care providers to the CQC, adding to data already collected by the Office for National Statistics [31]. Online systems were put in place for testing, with LTCFs required to register the results of all of LFD tests [86, 98].

### Reducing workload

Several steps were made to reduce workloads during the pandemic, facilitating transfers between settings. These included CQC cessation of routine inspections and launch of the Emergency Support Framework, removing the requirement for NHS Continuing Health Care assessments and suspending the need for funding panels for hospital discharge, where required [19, 29, 36, 37].

### Decision making and leadership

A key focus of implementing the guidance was on the role of leadership and decision making, including guidance for decision makers on applying ethical values and principles in urgent and uncertain circumstances for LTCF residents [20, 29]. This could be, for example, through applying a risk assessment on deciding on whether to admit a resident without a negative COVID-19 test, identification of clinically vulnerable staff or having difficult conversations, such as using the Vaccine Communications Toolkit for Adult Social Care [53, 105, 110].

### Training

Much of the guidance recognised the need for further training to implement the recommendations proposed. The training available included online webinars, guidance followed up by competency assessments and annexes, with decision-making flow charts or case studies of good practice. [19, 27, 29, 31].

### Technology and communication

The COVID-19 pandemic highlighted the need for secure, consistent communication and dissemination across services. Initiatives to facilitate this included implementing NHSmail in LTCFs, distributing Microsoft Teams to all care providers and offering discounted broadband deals to improve internet connectivity and to further introduce new technologies [19, 64]. Nearly all the guidance included in this review signposted to further guidance, including those produced by other agencies. In some cases, guidance was co-produced, such as using PPE in social care settings [80].

## Discussion

This review has identified publicly available policy, guidance and recommendations related to LTCFs, their residents, and staff, in England issued by the UK government during the COVID-19 pandemic. In doing so, the key guidance developments within the wider pandemic are provided in a narrative timeline of the main recommendations. Six themes of recommendations and seven areas of implementation emerging from the management of COVID-19 have been identified.

### Strengths and limitations

Despite widespread criticism of the management of the COVID-19 pandemic in LTCFs, academic literature on national policies is relatively scarce [162]. A strength of this review is its location of the guidance within the wider key events; the timeline illustrates how the approach to managing COVID-19 evolved over time, highlighting policies that could be implemented earlier in future pandemics. In addition, the review has focused on recommendations and their implementation, identifying how such polices were intended to be delivered and supported.

The review is timely in that it synthesises over two years of policy, providing a contextual reference for wider published outputs in the research area. Given the need for accessible, updatable guidance during the pandemic, all the documents included in this review were published online. Sourcing such data was challenging, in terms of finding the literature online and accessing original versions. In addition, not involving an academic librarian could be considered a limitation of this review. Without a clear repository of guidance, it is difficult to judge whether this review has included all the relevant documents published, despite following best practice methodological approaches [154, 155].

The pathways through which LTCFs accessed the policy recommendations and guidance discussed in this review, and subsequent updates, is an area for further research, however the need for guidance to be clear, consistent and accessible is apparent. In addition, the review focused solely on LTCFs for older adults, rather than wider adult social care, and did not extract data on updates to the guidance, however these updates mainly referred to local lockdowns and are reflected in the narrative timeline. In addition, whilst the review focused on policy in England, it may reflect practice internationally in other countries with comparative LTCFs for older adults.

## Connection to wider literature

The aim of this review was to identify, collate and synthesise guidelines, policy and recommendations, and has identified four areas which require further discussion in terms of managing pandemics in LTCFs.

Firstly, in some cases policies had unintended consequences; for example, the mass discharge of hospital patients to LTCFs has been associated with COVID-19 outbreaks, however the relative risk of transmission through hospital discharge compared to that of transmission from community routes into LTCFs is unclear [163].

Secondly, the predominant focus on preventing the spread of COVID-19 may have been at the detriment of wider health and wellbeing. It is possible that the risk of COVID-19, for some residents, may have been less of a priority compared to the impact on quality of life of not having contact with relatives or the effect of social isolation, however this was acknowledged relatively little in the policies included in this review.

Thirdly, the extent to which the policies identified were effective is debatable. In the case of residents approaching end of life, during which visits from relatives were allowed, one survey found that 18% of LTCFs surveyed did not allow visitors at the end of life, and of those that did 51% experienced challenges in providing bereavement support to relatives [164, 165]. In addition, despite the introduction of mental health and wellbeing resources for adult social care staff, health care workers in LTCFs reported experiencing high levels of stress, especially among those with personal health issues, and high levels of post-traumatic stress disorder [166–168].

Finally, the extent to which the policies issued were able to be implemented in LTCFs is also questionable. For example, the ability of non-purpose built LTCFs to successfully isolate residents from one another, is unclear. Alternatively, efforts to digitise LTCFs and introduce new technologies, such as remote conferencing, while a welcome development, were dependant on the availability of training and support required to introduce, embed and sustain such interventions [169–171].

Arguably, some of the areas that policies were aimed at were longstanding challenges faced by adult social care in England, and trying to address historic issues exacerbated by the pandemic could be difficult. For example, strategies to manage COVID-19 related staff shortages were central in the policies identified, despite high staff turnover, relatively low pay and a reliance on external agency staff across multiple sites existing pre-pandemic [145]. In addition, the pandemic highlighted the need for joint working between LTCFs and wider sectors, an area of concern that has existed for years prior to COVID-19 [172, 173].

From a more positive perspective, the experience of COVID-19 in LTCFs may have improved some long-standing challenges experienced by adult social care in England. Firstly, identification as key workers and albeit delayed access to household testing repositioned adult social care staff in line with the wider healthcare workforce. Secondly, one arguably successful policy was the introduction of the Capacity Tracker to collect data on LTCFs, and the wider implementation of upgrading technology in the process. The paucity of data on adult social care has also been highlighted prior to the pandemic, supporting further calls for development of a minimum dataset [174, 175].

## Implications for policy, practice and further research

From a policy perspective, this review has highlighted the need for effective, accessible, and timely guidance and recommendations on managing pandemics in LTCFs. In particular, the secondary effects of the outcomes of such policies, and how such impacts can be measured, beyond numbers of infections, outbreaks or deaths, requires further thought. For example, while hospital admissions from LTCFs declined during COVID-19; the extent to which this reflects appropriate care within the facility or unmet need is unclear [176].

The timing of such guidance also warrants further discussion, particularly in reference to asymptomatic presentation and testing availability [177]. The role of asymptomatic presentation was likely under-estimated in the early stages of the pandemic. In one study on COVID-19 symptomology within an LTCF, of 40% of residents who tested positive, 43% were asymptomatic, and 4% of staff tested positive, all of whom were asymptomatic [178]. In hindsight, had ‘whole home’ testing been available after one suspected case, asymptomatic cases could have been identified earlier, allowing more time for the updating and if necessary, implementing, of advance care plans, cohorting exposed residents and planning for potential staff shortages [179, 180].

The extent to which wider stakeholders, including LTCF staff, residents and relatives’ groups and charities such as the National Care Forum, were consulted during policy development, and how this involvement would have shaped the policy recommendations, is also unclear [181]. An example of the need for stakeholder engagement can be seen in vaccine hesitancy among LTCF staff [182]. In future, further engagement with wider stakeholders is needed to identify areas of importance to LTCF residents, relatives and staff during pandemics, and how policies can be successfully implemented on the ground.

In addition, a better understanding of the mechanisms by which LTCFs access policy recommendations could further enhance policy development in this area. Throughout the pandemic, there were repeated calls by LTCF managers for clear, consistent guidance, however the routes of dissemination through which guidance is accessed, and how these can be enhanced, needs further development [183, 184].

In terms of further research, this review has identified three areas of priority. Firstly, exploring why some LTCFs experienced the pandemic differently to others, and how this relates to the implementation of issued policies and guidance, is a priority. As of Dec 2020, 70% of all LTCFs in England had experienced a COVID-19 infection, and 33.1% of these had experienced multiple outbreaks [185]. The likelihood of outbreaks has been associated with higher bed occupancy, lower staff levels and the use of agency staff across multiples sites, however this knowledge base is far from complete [186, 187]. In comparison, in Canada 54% of resident deaths were in privately owned, profit oriented LTCFs, and in Australia COVID-19 outbreaks were associated with areas of increased community transmission and no face-to-face infection control training [188, 189]. Comparing the experiences of LTCFs internationally and understanding the mechanisms behind the differences between countries is a key area for exploration. In addition, inequalities in the number of COVID-19 infections in LTCFS in areas of higher and low deprivation require further investigation [190].

Secondly, further research is needed to explore how effective policies across the themes identified can be implemented in LTCFs, within the context of pre-existing challenges to adult social care and the immediate pressures of a pandemic. Ongoing research in some of these areas is already emerging, such as how LTCF staff can be supported in providing end of life care and delivering training on the use of PPE, however this needs expanding, with training ideally covering more than one aspect of pandemic management [168, 171, 191, 192].

Thirdly, international comparison to countries with comparable long-term care systems would support further development in this area. Initiatives such as the International Long-Term Care Policy Network are already making progress in this area, and developing an understanding of how other countries approached pandemic management in LTCFs could provide valuable learning for England [193].

In relation to changing practice, further research is needed to explore the extent to which policies were implemented, and the barriers, facilitators and challenges to doing so. Understanding how current approaches to providing care in this setting can be ‘pandemic proofed’, and whether there are preventative measures that could be addressed to avoid repeating the mistakes of the COVID-19 pandemic in the future could be beneficial. This could include sustaining a system to ensure equitable access to PPE supplies and testing facilities, or maintaining procedures limiting staff movement between sites.

There is also potential for wider discussion on how residents with dementia and those lacking mental capacity can be cared for [194]. While the policies issued referred to the specific care needs of older adults in managing COVID-19, further guidance on practical approaches to prognostic trajectory, advance care planning and recovery from COVID-19 could be useful [168, 195]. Recognising COVID-19 in residents with dementia can be especially problematic, often presenting atypically; with residents more likely to experience delirium and deteriorate relatively fast. These residents may be less able to understand social distancing or handwashing requirements, and experience the mental and emotional impact of isolation and decreased socialisation more severely [196].

Finally, managing the impact of COVID-19 on residents who are not infected is also an area of interest. As discussed by Burton et al., excess deaths, both COVID-19-related and non-COVID-19-related, were concentrated in LTCFs with a confirmed outbreak of COVID-19 [197], indicating that the extra burden of caring for residents with COVID-19 had a detrimental indirect impact on residents not infected. Again, how to ensure appropriate care is maintained during outbreaks for all residents requires further exploration.

## Conclusion

The impact of the COVID-19 pandemic on LTCFs in England should not be forgotten, and the opportunity to learn from the experience not missed. This review has provided an overview of key themes within the policy, guidance and recomendations issued, and identified areas for further development in terms of pandemic preparedness. As the ageing population continues to grow across the world and the long-term care needs of older adults increases, developing effective responses for managing future pandemics in LTCFs should remain a priority, in England and internationally.

### Supplementary Information

**Supplementary Material 1.**

## Data Availability

All data generated or analyzed during this study are included in this published article and its supplementary information files.

## Abbreviations

CQC: Care Quality Commission
DNACPR: Do Not Attempt Cardiopulmonary Resuscitation
ENTREQ: Enhancing Transparency in Reporting the synthesis of Qualitative research
HPT: Health protection team
IPC: Infection prevention and control
LFD: Lateral flow device
LRF: Local resilience forum
NHS: National Health Service
NSDR: National Supply Disruption Response
PPE: Personal protective equipment
PCR: Polymerase chain reaction
PRISMA: Preferred Reporting Items for Systematic Reviews and Meta-Analyses
UK: United Kingdom
