# Supplementary material for: Long term care facilities in England during the COVID-19 pandemic—a scoping review of guidelines, policy and recommendations
Source: BMC Geriatr. 2024 May 3;24:394. doi: 10.1186/s12877-024-04867-9 (PMC11069159; doi:10.1186/s12877-024-04867-9)
Supplement: Supplementary file 1 — Supplementary Material 1. [file 12877_2024_4867_MOESM1_ESM.docx]

| Guidance for social or community care and residential settings on COVID-19 (1)  Published: 25/02/2020  Author:  Public Health England (PHE) | **Aims:**  Guidance developed to assist social, community and residential care employers in providing advice to their staff on COVID-19, including how to help prevent spread respiratory infections, what to do if someone suspected or confirmed to have COVID-19 has been in a health or social care setting, or if staff come into contact with someone who is self-isolating or is a possible or confirmed case of COVID-19. |
| --- | --- |
|  | **Recommendations:**  **Preventing the spread of infection**   - The best way to prevent infection is to avoid being exposed to the virus, including washing hands often, covering your cough or sneeze with a tissue, then throwing the tissue in a bin, avoid touching your eyes, nose, and mouth with unwashed hands and to clean and disinfect frequently touched objects and surfaces. - If people feel unwell, they should stay at home and should not attend work. If staff are worried about their symptoms or those of a family member or colleague, please call National Health Service (NHS) 111. They should not go to their General Practitioner (GP) or other healthcare environment.   **Guidance on facemasks**   - During normal day-to-day activities facemasks do not provide protection from respiratory viruses, such as COVID-19 and do not need to be worn by staff in any of these settings. Facemasks are only recommended to be worn by infected individuals when advised by a healthcare worker, to reduce the risk of transmitting the infection to other people. It remains very unlikely that people receiving care in a care home or the community will become infected. - PHE recommends that the best way to reduce any risk of infection for anyone is good hygiene and avoiding direct or close contact (within 2 metres) with any potentially infected person.   **What to do if an employee becomes unwell and believe they have been exposed to COVID-19**   - If the staff, member of the public or resident has not been to specified areas in the last 14 days, then normal practice should continue.   **What to do if someone with confirmed COVID-19 has recently been in the office, workplace or residential setting**   - Closure of the office, workplace or residential setting is not recommended.   **What to do if someone in the office, workplace or residential setting has had contact with a confirmed case of COVID-19**   - If a confirmed case is identified in this setting, the local Health Protection Team (HPT) will provide the relevant people with advice. - Contacts are not considered cases and if they are well, they are very unlikely to spread the infection to others, those who have had close contact will be asked to self-isolate at home or in their own room in a care or residential home for 14 days from the last time they had contact with the confirmed case and follow the home isolation advice sheet. They will be actively followed up by the HPT. - People who have not had close contact with the confirmed case do not need to take any precautions and can continue their routines as usual.   **Additional guidance**   - No restrictions or special control measures are required in these settings while a member of staff or resident is waiting for laboratory test results for COVID-19. In particular, there is no need to close or send staff home at this point. - Specific guidance for staff members who become unwell and have travelled to affected countries, or those returning from travel overseas to affected areas, including self-isolating, are advised to call NHS 111 for advice, and advised against going to their GP or other health care environment. - Specific guidance for people if they have returned from travel anywhere else in the world within the last 14 days. These people can continue to attend work and go about their daily routine unless they have been informed otherwise by their local HPT. - Guidance provided on cleaning the office, workplace or residential setting where there are confirmed cases of COVID-19, and rubbish disposal including tissues. - Guidance provided for social and community care staff visiting patients at home or providing care to residents with potential COVID-19, including conducting a risk assessment to assess the suitability of a domiciliary visit. If during a telephone consultation with a patient or their representative it is thought that COVID-19 is possible, then a face-to-face assessment must be avoided. Instead, call NHS 111 and arrange for a clinical assessment to be made before proceeding. - If the person is asymptomatic or has a negative COVID-19 test - there is no need to change the approach. - If the person is symptomatic - further guidance on isolating the patient and conducting a full risk assessment with an infection control specialist is provided, including calling NHS 111. - If the person has a positive COVID-19 test - a contact tracing exercise will be undertaken by the local HPT. - If the patient is critically ill and requires an urgent medical attention or ambulance transfer to a hospital - inform the ambulance call handler of the potential links to COVID-19. |
|  | **Implementation:**   - The guidance is intended for the current position in the UK where there is currently no transmission of COVID-19 in the community. It is therefore very unlikely that anyone receiving care in a care home or the community will become infected. - Generally, these infections can cause more severe symptoms in people with weakened immune systems, older people, and those with long-term conditions like diabetes, cancer and chronic lung disease. - There is currently little evidence that people without symptoms are infectious to others. - Guidance includes links to further information including the PHE Blog and NHS UK.   **What to do if someone with confirmed COVID-19 has recently been in the office, workplace or residential setting**   - The management will be contacted by the PHE local HPT, who will provide contract tracing, conduct risk assessments, and advise on cleaning, and management and control of outbreaks of infectious disease in the community.   **What to do if someone in the office, workplace or residential setting has had contact with a confirmed case of COVID-19**   - Guidance includes links to PHE local HPT and home isolation advice sheet. - The local HPT will provide advice on cleaning.   **What social, community and residential care settings need to do now**   - If any staff do become infected through travel to affected countries, the local HPT will contact the care provider to take them through a risk assessment for the particular setting. - HPTs are part of PHE and will provide advice and guidance on infectious disease and non-infectious environmental hazards, manage and control outbreaks of infectious disease in the community and are a source of expert advice on new infections. - The local public health team is led by the Director of Public Health and will link closely with the Director of Adult Social Services in working with partners locally to respond to any cases of this infection. |
|  | **Intended outcomes:**  …prevent spread of all respiratory infections, including COVID-19, by avoiding being exposed to the virus, and by reducing the risk of transmitting the infection to other people. …self-isolating to allow closer monitoring in order to identify early symptoms, and to enable prompt medical action if required. |

| COVID-19 Hospital Discharge Service Requirements (2)  Published: 19/03/2020  Author:  Department for Health and Social Care | **Aims:**  Guidance sets out the hospital discharge service requirements for all NHS trusts, community interest companies and private care providers of acute, community beds and community health services and social care staff in England. It also sets out requirements around discharge for health and social care commissioners. |
| --- | --- |
|  | **Recommendations:**   - Unless required to be in hospital, patients must not remain in an NHS bed. - Acute and community hospitals must discharge all patients as soon as they are clinically safe to do so. Transfers from the ward should happen within one hour of a decision being made to a designated discharge area, with discharge from hospital as soon after that as possible, normally within 2 hours. - The discharge to assess model is based on using four clear pathways for discharging patients.   **Specific to care homes:**  Whilst most people will be discharged to their homes, a very small proportion will need and benefit from short or long term residential or nursing home care. The  Discharge Service will be able to access live information from a national community bed tracker system. The existing North of England Commissioning Support (NECS) care home tracker will be extended to cover all care home places, all NHS community hospital beds and hospice beds. All providers must sign up and start using the tracker by 23 March 2020  **What does this mean for patients?**  For patients whose needs are too great to return to their own home (about 5% of patients admitted to hospital) a suitable rehabilitation bed or care home will be arranged. During the COVID-19 pandemic, patients will not be able to wait in hospital until their first choice of care home has a vacancy. This will mean a short spell in an alternative care home. Care coordinators will follow up to ensure patients are able to move as soon as possible to their long-term care home.  **Finance support and funding flows**  The Government has agreed to fully fund the cost of new or extended out-of-hospital health and social care support packages, referred to in this guidance, for people being discharged from hospital or who would otherwise be admitted into it for a limited time, to enable quick and safe discharge and more generally reduce pressure on acute services. Further guidance provided on finance support and funding flows. |
|  | **Implementation:**   - The guidance must be adhered to from 19 March 2020. - Acute and community hospitals must keep a list of all those suitable for discharge and report on the number and percentage of patients on the list who have left the hospital and the number of delayed discharges through the daily situation report. - Therefore, NHS Continuing Health Care (CHC) assessments for individuals on the acute hospital discharge pathway and in community settings will not be required until the end of the COVID-19 emergency period. - The Government has agreed the NHS will fully fund the cost of new or extended out- of-hospital health and social care support packages, referred to in this guidance. This applies for people being discharged from hospital or who would otherwise be admitted into it, for a limited time, to enable quick and safe discharge and more generally reduce pressure on acute services. - Guidance supplemented by specific action cards outlining how key roles should work differently during this period, which will be published separately and discussed as part of webinar sessions on these changes.   **Specific to care homes:**  **What are the actions for acute care organisations and staff?**  Provides guidance at ward level, for hospital discharge teams and hospital clinical and managerial leadership teams. At ward level, social care colleagues should be involved in daily ward reviews. This will help with the early identification of any possible support, placement or housing issues with discharge and allow the multidisciplinary team (MDT) to undertake arrangements in good time.  **What are the actions for councils and adult social care services?**   - Identify an executive lead for the leadership and delivery of the discharge to assess model. - Redeploy social work staff from the hospital setting to community settings to support discharged patients. Safeguarding investigations should continue to take place in a hospital setting if necessary. - Ensure there are robust tracking mechanisms to track care placements so that care users do not get lost in the system at a time of very rapid response. - Suspend need for funding panels for hospital discharge during the level 4 incident, with additional funding available to local authorities to cover any increased costs during this period. - Provide social care capacity to work alongside local community health services to provide a single point of contact for hospital staff. - Support real time communication between the hospital and the single point of contact, not just by email. - Provide capacity to review care provision and change, if necessary, at an appropriate point. - Work closely with community health providers over the provision of equipment. - Ensure there is 7-day working for community social care teams (to be commissioned by local authorities). - Deploy adult social care staff flexibly in order to avoid any immediate bottlenecks in arranging step down care and support in the community and at the same time focusing on maintaining and building capacity in local systems.   **What are the actions for care providers?**   - Maintain capacity and identify vacancies that can be used for hospital discharge purposes. - From 23 March 2020, adopt and implement the Capacity Tracker during the COVID-19 outbreak to make vacancy information available to NHS and social care colleagues in real time. - Providers of care homes, in partnership with their local Primary Care Networks (PCNs) and Community Health Providers, should consider how best to support residents, and where already in place, embed the Enhanced Health in Care Home Framework in line with timescales already outlined by NHS England and NHS Improvement which have been communicated to primary care providers. This will ensure their residents are better supported (7 days a week) by the NHS. - Implement NHSmail in their care home from 23 March 2020, to ease communication between NHS and social care colleagues. From 23 March 2020, faster NHSmail roll-out will be available to all care providers, to support safe and secure transfer of information. NHSmail is accredited for sharing patient identifiable and sensitive information, meaning it meets a set of information security controls that offer an appropriate level of protection against loss or inappropriate access. - To improve communication between health and social care during the COVID-19 outbreak, NHSX is speeding-up the roll-out of NHSmail and temporarily waiving the completion of Data Security Protection Toolkit (DSPT) to allow for quicker on boarding. This is in-line with information governance guidance for COVID-19. - These are temporary measures to improve communication during COVID-19. NHSX is committed to enabling care providers to choose the right communication solutions for them. Providers will be asked to give their own assurance that they are secure and post-COVID-19, afterward NHSmail regional teams will take providers through the full DSPT process, supporting them to accredit their secure email system or NHSmail for sharing in future.   **Additional resources and support**  To support implementation, NHS England will be running webinars to run through the guidance and provide local areas with the opportunity to ask questions. Links provided to supporting guidance, and annexe includes patient discharge choice leaflets. |
|  | **Intended outcomes:**  Implementing these service requirements is expected to free up to at least 15,000 beds by 27 March 2020, with discharge flows maintained after that.  Free acute beds can be immediately used by someone being admitted who is acutely unwell. |

| Responding to COVID-19: the ethical framework for adult social care (3)  Published: 19/03/2020  Author:  Department for Health and Social Care | **Aims:**  The framework intends to provide support to ongoing response planning and decision-making to ensure that consideration is given to ethical values and principles when organising and delivering adult social care as COVID-19 develops.  Framework is aimed at planners and strategic policy makers at local, regional and national level to support response planning and organisation of adult social care during and as COVID-19 develops. It also aims to support the work of professionals and others in the health and social care workforce who are developing policies and responding to the outbreak, in line with their own professional codes of conduct and regulations. |
| --- | --- |
|  | **Recommendations:**  **Values and principles**  This section outlines each ethical value and principle and associated actions and best practice when considering and applying them. These should be considered alongside professional codes of conduct and the most recent official guidance and legislation where these apply.   - Respect - Reasonableness - Minimising harm - Inclusiveness - Accountability - Flexibility - Proportionality - Community |
|  | **Implementation:**  Guidance should be considered alongside professional codes of conduct and the most recent official guidance and legislation where these apply. It might be useful to use the framework as a checklist to ensure ethical considerations are taken into account, however, the values and principles described in this document are not exhaustive. When implementing the ethical values and principles in urgent and uncertain circumstances, professionals may encounter tension between them which will require a judgement to be made on the extent that a particular value or principle can be applied in the context of each particular decision. |
|  | **Intended outcomes:**  Recognising increasing pressures and expected demand, it might become necessary to make challenging decisions on how to redirect resources where they are most needed and to prioritise individual care needs. This framework intends to serve as a guide for these types of decisions and reinforce that consideration of any potential harm that might be suffered, and the needs of all individuals, are always central to decision-making. |

| Admission and Care of Residents during COVID-19 Incident in a Care Home (4)  Published: 02/04/2020  Author:  [Department of Health and Social Care](https://web.archive.org/web/20200404122850/https:/www.gov.uk/government/organisations/department-of-health-and-social-care), [Care Quality Commission](https://web.archive.org/web/20200404122850/https:/www.gov.uk/government/organisations/care-quality-commission), [Public Health England](https://web.archive.org/web/20200404122850/https:/www.gov.uk/government/organisations/public-health-england), and [NHS England](https://web.archive.org/web/20200404122850/https:/www.gov.uk/government/organisations/nhs-commissioning-board) | **Aims:**  Guidance supports the safe admission and care for patients during the pandemic.  …to support care home providers to protect their staff and residents, ensuring that each person is getting the right care in the most appropriate setting for their needs. |
| --- | --- |
|  | **Recommendations:**  Guidance covers six key areas:  **Admission of residents**  Guidance states that "if an individual has no COVID-19 symptoms or has tested positive for COVID-19 but is no longer showing symptoms and has completed their isolation period, then care should be provided as normal". Hospital discharge service and staff will clarify with care homes the COVID-19 status of an individual and any COVID-19 symptoms, during the process of transfer from a hospital to the care home. Tests will primarily be given to:   - all patients in critical care for pneumonia, acute respiratory distress syndrome (ARDS) or flu like illness. - all other patients requiring admission to hospital for pneumonia, ARDS or flu like illness. - where an outbreak has occurred in a residential or care setting, for example long-term care facility or prisons.   Negative tests are not required prior to transfers / admissions into the care home.  **Caring for residents, depending on their COVID-19 status**  If a resident who has been discharged from hospital and has tested positive for COVID-19, the discharging hospital will provide the following information upon discharge:   - The date and results of any COVID-19 test. - The date of the onset of symptoms. - A care plan for discharge from isolation.   Keeping asymptomatic residents safe and monitoring symptoms: Care home providers should follow social distancing measures for everyone in the care home, wherever possible, and the shielding guidance for the extremely vulnerable group. Care homes should implement daily monitoring of COVID-19 symptoms amongst residents and care home staff.  Symptomatic residents: Any resident presenting with symptoms of COVID-19 should be promptly isolated and separated in a single room with a separate bathroom, where possible.  Testing residents:   - Single symptomatic resident: Testing may be offered following contact with NHS 111 or according to local protocol for swabbing and testing. - More than one symptomatic resident: Inform the HPT. They may arrange swabbing for up to 5 initial possible cases to confirm the existence of an outbreak. Testing all cases is not required as this would not change subsequent management of the outbreak.   Continue all strict control measures including isolation, cohorting and infection control measures until results for all residents who were tested are obtained or until the period of isolation has been completed.  **Reporting of COVID-19 cases**  Inform the local HPT of two or more possible or confirmed cases within the care home. The HPT will advise on further communication to local infection control teams and local authority colleagues and Clinical commissioning groups (CCG)s.   - The HPT will provide advice and support along with local authority partners to help the care home to manage the outbreak. - Follow the outbreak control measures advised by the HPT. - The outbreak can be declared over once no new cases have occurred in the 14 days since the appearance of symptoms in the most recent case.   **Providing care after death**  The infection control precautions described in this document continue to apply whilst an individual who has died remains in the care home.  **Advice for staff**  Staff who come into contact with a COVID-19 positive resident without PPE can remain at work. This is because in most instances this will be a short-lived exposure, unlike exposure in a household setting that is ongoing. These are guiding principles and there should be an individual risk assessment based on staff circumstances, for example staff who are vulnerable should be carefully assessed when assigning duties, and where a possible or confirmed COVID-19 case is present in a care home, efforts should be made to cohort staff caring for that person.  For staff who have COVID-19 symptoms, they should:   - Not attend work if they develop symptoms. - Notify their line manager immediately. - Self-isolate for 7 days, following the guidance for household isolation. - Care home capacity will be monitored via the capacity tracker and this data will be shared with Local Resilience Forums (LRFs) via the daily national Situation Reports to support capacity planning and response. However, where providers consider there to be imminent risks to the continuity of care, such as the potential closure of a service, they should raise this with the local authority without delay.   **Supporting existing residents that may require hospital care**  If care staff think one of their residents may need to be transferred to hospital for urgent and essential treatment, consider the following checklist:  If a resident shows symptom of COVID-19:   - Assess the appropriateness of hospitalisation: consult the resident's Advance Care Plan/Treatment Escalation Plan and discuss with the resident and/or their family member(s) or Lasting Power of Attorney as appropriate following usual practice to determine if hospitalisation is the best course of action for the resident.   If hospitalisation is required:   - Follow IPC guidelines for patient transport. - Inform the receiving healthcare facility that the incoming patient has COVID-19 symptoms.   If hospitalisation is not required:   - follow IPC, and isolation procedures and consult the resident’s GP for advice on clinical management / end of life care as appropriate.   If a resident requires support with general health needs:   - Consult the resident's Advance Care Plan. - Consult the resident's GP and community healthcare staff to seek advice. - Alternatively, contact NHS 111 for clinical advice.   Postpone routine non-essential medical and other appointments.   - Review and postpone all non-essential appointments (medical and non-medical) that would involve residents visiting the hospital or other health care facilities. - If medical advice is needed to manage routine care, consider arranging this remotely via a phone call with the GP or named clinician. |
|  | **Implementation:**  Guidance states that to support care providers, care homes need to have access to the right knowledge, staff and resources, so they are equipped to deliver care in this challenging time. In return care homes will be supported in the following ways:   - Ensure they have the information and support they need to safely admit and care for patients during the pandemic. - Ensure a longer-term supply of all aspects of personal protective equipment (PPE) for care homes - and home care providers - so that staff can provide care, as well as providing a national supply disruption line for immediate concerns. - Establish the Capacity Tracker as the single mechanism across the country to report bed vacancies and help manage demand during this incident. - Work with commissioners to ensure fair and prompt payment for the existing care commitments and additional care provided during the response to the pandemic, recognising that both PPE and staffing costs are higher than usual. - Make NHSMail available for secure communication and transfer of information and this must be used for communication with the NHS.   Guidance is intended for care homes, local HPTs, local authorities, CCGs and registered providers of accommodation for people who need personal or nursing care.  **Caring for residents, depending on their COVID-19 status**  Signposts to NHS 111 COVID-19 service for advice on assessment and testing. If further clinical assessment is advised, contact the GP. Care home staff should note that people with dementia and cognitive impairment may be less able to report symptoms because of communication difficulties, and therefore staff should be alert to the presence of signs as well as symptoms of the virus. This could include delirium, which people with dementia are more prone to suffer from if they develop an infection.  Annexe contains isolation of COVID-19 symptomatic patients:   - Single case - Isolation of a symptomatic resident - More than one case - Cohorting of all symptomatic residents: - Symptomatic residents should ideally be isolated in single occupancy rooms. - Where this is not practical, cohort symptomatic residents together in multi-occupancy rooms. Staff caring for symptomatic patients should also be cohorted away from other care home residents and other staff, where possible/practical. - Isolation and cohorting of contacts: Isolation of contacts individually in single rooms for 14 days after last exposure to a possible or confirmed case. Cohorting of contacts within one unit rather than individually: and Protective cohorting of unexposed residents:   Annex contains instructions on receiving residents being discharged from hospital.  **Reporting of COVID-19 cases**  Annexe contains definitions of COVID-19 cases and contacts, including possible case of COVID-19 in the care home, confirmed case of COVID-19, infectious case, resident contacts, staff contacts and an outbreak.  **Providing care after death**  Links to further information provided.  **Advice for staff**  Guidance links to PPE that should be worn when caring for possible or confirmed COVID-19 patients, and guidance on risk assessments for staff.  **National support available to implement this guidance**  To support implementation, NHS England and Improvement, in collaboration with other national organisations, will be running webinars to build on this guidance and provide Care Homes and their partner organisations with the opportunity to ask questions. For support to use the Capacity Tracker, NECS has set up a contact centre to support those providers who are being required to register and update their information daily.  Annexe includes COVID-19 symptoms and including persons at higher risk of COVID-19 in a care home setting:   1. Anyone who falls under the category of extremely vulnerable should follow the shielding guidance to protect these individuals. 2. Anyone aged 70 years or older (regardless of medical conditions) should follow social distancing guidance for the clinically vulnerable. 3. Anyone aged under 70 years with an underlying health condition – for most this will align with eligibility for the flu jab on medical grounds – should follow social distancing guidance for the clinically vulnerable.   Symptoms may be more nuanced in older people with co-morbidities in care homes who may present with influenza Like Illness, respiratory illness, new onset confusion, reduced alertness, reduced mobility, or diarrhoea, and sometimes do not develop fever. This may be true for COVID-19, so such changes should alert staff to the possibility of new COVID-19 infection.  **Communications**  Considerations for visitors and non-essential staff:   - Family and friends should be advised not to visit care homes, except next of kin in exceptional situations such as end of life. - Follow the social distancing guidance. - Visitors should be limited to one at a time to preserve physical distancing. - Visitors should be reminded to wash their hands for 20 seconds on entering and leaving the home and catch coughs and sneezes in tissues. - Visitors to minimise contact with other residents and staff (less than 15 minutes / 2 metres etc.). - Alternatives to in-person visiting should be explored, including the use of telephones or video, or the use of plastic or glass barriers between residents and visitors. - Visitors should visit the resident in their own room directly upon arrival and leave immediately after the visit. - Cancel all gatherings and plan alternative arrangements for communal activities which incorporate social distancing.   **Support for care home staff -**   - Review sick leave policies and occupational health support for care home staff and support unwell staff to stay at home as per PHE guidance. Support for employers is available here. - Staff who have a symptomatic household member must stay at home and not leave the house for 14 days. The 14-day period starts from the day when the first person in the house became ill. If the staff member develops symptoms during this period, they can return to work 7 days after their symptoms started and they are no longer symptomatic. Further guidance is available here. - Staff who fall into the clinically vulnerable group should not provide direct care to symptomatic residents. - Ensure staff are provided with adequate training and support to continue providing care to all residents. - All care homes should have a business continuity policy in place including a plan for surge capacity for staffing, including volunteers.   Additional annexes on IPC measures, PPE and decontamination and cleaning processes for care homes with possible or confirmed cases of COVID-19, and use of Capacity Tracker to support effective discharge planning and continue care outside of hospitals. |
|  | **Intended outcomes:**  … to avoid the virus spreading to other residents in the care home and stop staff members becoming infected. HPTs will provide advice and support to help the care home to manage outbreaks. Staff should immediately instigate full infection control measures to care for the resident with symptoms, which will avoid the virus spreading to other residents in the care home and stop staff members becoming infected. |

| COVID-19: management of staff and exposed patients and residents in health and social care settings (5)  Published: 04/04/2020  Author:  Public Health England | **Aims:**  Not explicitly stated. |
| --- | --- |
|  | **Recommendations:**  **Staff exposures**   - Health care workers who come into contact with a suspected or confirmed COVID-19 patient while not wearing PPE can remain at work, as this will most likely be a short-lived exposure. - Health care workers should not attend work if they develop symptoms, notify their line manager immediately and self-isolate. The guidance principles should be applied in the context of individual staff risk assessments. - Symptomatic staff can return to work on day 8 after the onset of symptoms, if clinical improvement has occurred (further guidance on symptoms provided).   **Staff return to work criteria**  Symptomatic staff can return to work:   - on day 8 after the onset of symptoms if clinical improvement has occurred and they have been afebrile (not feverish) for 2 days, - if a cough is the only persistent symptom on day 8, they can return to work (post-viral cough is known to persist for several weeks in some cases).   **Patient exposures**   - In-patients who are known to have been exposed to a confirmed COVID-19 patient should be isolated or cohorted until their hospital admission ends, or until 14 days after last exposure. - If symptoms or signs consistent with COVID-19 occur in the 14 days after exposure then relevant diagnostic tests, including the COVID-19 test, should be performed. |
|  | **Implementation:**  **Staff exposures**   - Health care workers are directed to their occupational health department, use the NHS 111 online COVID-19 service or for a medical emergency dial 999 if their symptoms do not get better after 7 days, or their condition gets worse, - The recommended PPE to be worn when providing care for COVID-19 patients is described in the IPC guidance.   **Patient exposures**   - On discharge, patients should be given written advice to stay at home and referred to the stay at home guidance if less than 14 days has elapsed since their exposure – links to written advice provided. |
|  | **Intended outcomes:**  Not explicitly stated. |

| Coronavirus (COVID-19): looking after people who lack mental capacity - Guidance for Hospitals, Care Homes and Supervisory Bodies (6)  Published: 09/04/2020  Author:  [Department of Health and Social Care](https://web.archive.org/web/20200404122850/https:/www.gov.uk/government/organisations/department-of-health-and-social-care) | **Aims:**  Emergency guidance intended for is for all decision-makers in England and Wales who are caring for, or treating, a person who lacks the relevant mental capacity. |
| --- | --- |
|  | **Recommendations:**  Guidance recognises that during the pandemic, it may be necessary to change a person’s usual care and treatment arrangements to, examples include:   - providing treatment to prevent deterioration when they have or are suspected to have contracted COVID-19, - moving them to a new hospital or care home to better utilise resources, including beds, for those infected or affected by COVID-19, and - protecting them from becoming infected with COVID-19, including support for them to self-isolate or to be isolated for their own protection.   New arrangements may be more restrictive than they were, for the person, before the pandemic. It is important than any decision made under the Mental Capacity Act (MCA) is made in relation to that individual; MCA decisions cannot be made in relation to groups of people. Guidance reiterates that If the person lacks capacity to provide consent, the decision maker should where necessary make a best interests decision under the MCA regarding the care or treatment that needs to be provided. |
|  | **Implementation:**  This guidance is only valid during the COVID-19 pandemic and applies to those caring for adults who lack the relevant mental capacity to consent to their care and treatment. In most cases, it will be sufficient to make a best interest’s decision in order to provide the necessary care and treatment and put in place the necessary arrangements, for a person who lacks the relevant mental capacity to consent to the arrangements during this emergency period.  The Deprivation of Liberty Safeguards (DoLS) process will not apply to the vast majority of patients who need life-saving treatment who lack the mental capacity to consent to that treatment, including treatment to prevent the deterioration of a person with COVID-19.  In cases where a new authorisation may be needed, an urgent authorisation can come into effect instantly when the application is completed and lasts for up to a maximum of seven days, which can be extended for a further seven days if required. During the pandemic, a shortened form is needed to grant an urgent authorisation and request an extension to that urgent authorisation, from the supervisory body.  Annex includes decision-making flow chart for decision makers in hospitals and care homes. |
|  | **Intended outcomes:**  Guidance will help decision makers to make these decisions quickly and safely, whilst keeping the person at the centre of the process. |

| COVID-19: our action plan for adult social care (7)  Published:  15/04/2020  Author:  [Department of Health and Social Care](https://web.archive.org/web/20200404122850/https:/www.gov.uk/government/organisations/department-of-health-and-social-care) | **Aims:**  The action plan sets out the approach for all settings and contexts in which adults receive social care. The action plan supports care providers, the care workforce, unpaid carers, local authorities and the NHS to maintain services and continue to provide high quality and safe social care throughout the pandemic. |
| --- | --- |
|  | **Recommendations:**  **Four pillar approach:**  **Controlling the spread of infection**   - **Support and advice on how to keep care settings safe:** publication of guidance that sets out advice for those affected on how to minimise the risks of transmission through good infection control practices and sets out some of the steps that local authorities and the NHS should take to support care providers through the pandemic. - **Provision and use of PPE:** government support to the supply and distribution to the care sector, to ensure an emergency supply is in place. In response to requests from the care sector for consistent messaging and training on PPE, PHE are producing training videos on donning and doffing PPE, and providing tailored insights into how the PPE guidance applies in care settings. In response to distribution, an emergency drop of 7 million PPE has been provided to the Care Quality Commission (CQC) registered care providers. In addition, 23 million items of PPE were released for onward sale to social care providers, to support the existing supplier network, including the release of a further 34 million items of PPE across LRFs. - **Managing outbreaks:** signposting to HPTs for outbreak management, which may include isolating cases, determining the best approach to isolating residents and reinforcing infection control practices, including an appropriate response to PPE, staffing, and controlling visitors. Move to testing all symptomatic residents. - **Safe discharge from the NHS to social care settings:** any patient who does not need an NHS bed will continue to be discharged in line with the current discharge requirements. Move to institute a policy of testing all residents prior to admission to care homes, where a test result is still awaited, the patients will be discharged and pending the result, isolated in the same way as a COVID-19 positive patient. Some care providers will be able to accommodate COVID-19 positive individuals through effective isolation strategies or cohorting policies. If not, local authorities will be asked to secure alternative appropriate accommodation and care for the remainder of the required isolation period, supported by £1.3 billion available to support enhanced discharge from the NHS. Where a resident tests negative, isolation for 14 days is still recommended. For individuals coming from the community, residents will be tested prior to admission.   **Supporting the workforce**   - **Ensuring we have the staff that they need:** Testing for social care workers and those in their household who have symptoms enabled through local test centres, in line with NHS staff and their families. Designation of social care staff as key workers, allowing children of those working in social care to continue to attend school where there is no safe option for them to stay at home. Launch of a new national recruitment campaign to attract people to the social care workforce. Temporary arrangements to provide Disclosure and Barring Service (DBS) checks and fast-track emergency checks of the Adults’ and Children’s Barred Lists free of charge, to aid recruitment. Easier access to rapid online induction training for new staff through Skills for Care. Return to work of social workers, occupational therapists, deployment of nurses and nursing students and working to enable these volunteers to carry out appropriate tasks in social care also supported. - **Security and wellbeing**: Guidance refers to with £1.6 billion of additional funding provided local authorities in March 2020 which could be used to pay for the cost of backfilling shifts while as far as possible maintaining income for those that are unable to work as a result of the public health advice and wider social distancing measures. This is in addition to statutory sick pay being paid from the first day of sickness and increases in universal and working tax credit (to support staff picking up extra shifts) and furlough. In terms of wellbeing, extension of a text messaging support service for social care staff, free on all major mobile networks, to go for support. In addition, extending the package of support that is available to the NHS to the social care sector, including access to a dedicated website with a range of resources to help individuals and their teams manage. A dedicated free-to-caller support helpline extended to all social care workers. Skills for Care is creating a package of support for registered managers, recognising that they are facing particular challenges, and a new CARE branded website and app for the social care workforce being introduced. Establishment of the CARE brand to sit alongside the NHS brand in England. - **Using technology to support social care and quality of life:** launch of the TechForce19 competition to reach out to innovators who can support the elderly, vulnerable and self-isolating during COVID-19, such as by enabling remote care both in care homes, and optimising staffing in the care sector. Facebook will provide up to 2,050 portal video calling devices for free to hospitals, care homes and other settings, and 50 devices have already been deployed to pilot sites. Publishing of new guidance to assist social, community and residential care employers during the COVID-19 outbreak to support care while being mindful of handling people’s information securely. In addition, accelerated the rollout of NHS Mail and Microsoft Teams for care providers to communicate with healthcare providers.   **Supporting independence, people at the end of life, and responding to individual needs**   - **Supporting those who require health and care services:** people in care homes may be offered telemedicine consultations, however it may be clinically necessary for GPs to come into direct contact with people to provide them with the necessary treatment and care. Where a home care provider or care home is caring for someone suffering from more severe COVID-19 symptoms, the individual may need to be admitted to hospital. Where appropriate, the provider and the individual should receive additional support from local primary care providers, NHS community services and hospice-at-home to enable the best possible quality of care within the home setting, including home oxygen services and palliative care, in line with the wishes of the individual. - **Supporting people at the end of their lives:** Visits at the end of life are important and should continue, despite recommendations that care homes limit unnecessary visits. The blanket application of advance care plans, including Do Not Attempt Resuscitation orders, to any group of people is unacceptable. End of life care, including palliative care, must continue to be planned in a holistic way involving social care, community nursing, general practice, occupational therapy, and others.   **Supporting local authorities and the providers of care**   - **Funding:** two tranches of extra funding to local authorities – £1.6 billion on 19 March 2020 and a further £1.6 billion on 18 April 2020 – to support them in meeting pressures across the range of public services, including adult social care, along with £1.3 billion via the NHS specifically to support safe and timely discharge from hospitals into care. - **Collaboration across services:** General practice to identify more at-risk patients, who live in care homes, and work with community service providers to co-ordinate interventions. Regular care home rounds should be delivered virtually unless a physical presence is required for clinical reasons. - **Local and national oversight:** Local authorities to draw on all available flows of information, including the Capacity Tracker and links with care providers, to identify and address any emerging risks. CQC to ensure safety and quality standards are maintained/improved using the Emergency Support Framework, which offers providers support and advice, and helps local and national system partners identify and respond to safety concerns. - **Emergency response:** For adult social care, the lead role in responding to incidents is with the local authority. - **Monitoring:** The Capacity Tracker, a new tracker of key adult social care, collates daily information on bed capacity, workforce absences, PPE levels, and overall risks in care homes. Increased ability to track deaths of people with COVID-19 in care homes, combining Office for National Statistics (ONS) and CQC data. The Department for Health and Social Care (DHSC) is funding a national support offer to the adult social care sector worth almost £9 million to give local authorities access to expert advice and support, in addition to providing resources to the Social Care Institute for Excellence (SCIE) to help bring partners together virtually to share best practice in real time. CQC’s ‘Encouraging people to give feedback on care’ campaign collects intelligence from people who use adult social care, mental health and learning disability services and care staff. CQC and Think Local Act Personal are also working on proposals to understand the impact of Care Act easements and COVID-19 more widely on people with care and support needs. - **Communications**: Continue to publish guidance in response to the changing situation and the sector’s requests for greater information, while seeking to ensure guidance is clear and practical and that messages from government are joined-up and non-duplicative. |
|  | **Implementation:**  **Controlling the spread of infection**   - Signposting to the governments PPE plan and PPE guidance, which includes guidance for usage in the social care sector. Keeping under review what other forms of training and support may be required locally to ensure safety and respond to the needs of staff working in the sector. - LRFs to manage the local response to the pandemic. - National Supply Disruption Response (NSDR) system mobilised to respond to emergency PPE requests, alongside a 24/7 helpline for providers who have an urgent requirement (i.e. stock in less than 72 hours) and an express freight desk solution. A parallel supply chain will also be set up to take and dispatch orders directly from/ to health and social care providers. - Development of a new unit to identify and buy PPE supplies from across the globe as well as encouraging UK manufacturers to produce PPE in a national call to action. - Managing outbreaks through HPTs and local authorities. - Case studies of good practice provided.   **Supporting the workforce**   - CQC is leading co-ordination of testing and are contacting all registered care providers. - Development of a national campaign website which links to advertised social care jobs. - Development of a new online platform with access to online training and multiple job opportunities via a matching facility. - Allowing furloughed workers from other sectors to undertake paid employment in social care. - Swifter recruitment; temporary DBS arrangements and Skills for Care rapid online induction. - Major supermarkets asked to give social care workers access to priority hours, and to help care workers and care providers shop for supplies for the people they care for.   **Supporting independence, people at the end of life, and responding to individual needs**   - Signposts to guidance for GPs on managing essential face-to-face services. - Signposts to infection control guidance during end of life visits. - Everyone at risk of losing mental capacity or nearing the end of their life should be offered the opportunity and supported, if they wish, to develop advance care planning.   **Supporting local authorities and the providers of care**   - From Friday 10 April 2020, CQC has asked care providers to let CQC know if a resident died from either suspected or confirmed COVID-19. |
|  | **Intended outcomes:**  **Controlling the spread of infection**  … to help minimise the spread of infection within all care settings.  …guidance sets out advice for those affected on how to minimise the risks of transmission through good infection control practices and sets out some of the steps that local authorities and the NHS should take to support care providers through the pandemic.  … minimise the spread of the COVID-19 virus in social care, while also ensuring safe and timely discharge from NHS beds.  **Supporting the workforce**  … will help give workers, their families and those that they care for peace of mind and help providers who are struggling with staff absences.  … to ensure that those working in social care have the security necessary so that the only factor in their decision to work, isolate or shield themselves, is that same public health advice given to everybody in order to keep individuals, families and the wider public safe.  … aims to ensure that staff who are generally able to work but are unable to do so for short periods of time because they are unwell or self-isolating, do not lose out financially because they are doing the right thing.  … ensure that social care gets the recognition and parity of esteem that it deserves.  … to build recognition of providers of social care as part of the wider team delivering an essential public service.  … to attract 20,000 people into social care over the next 3 months.  … aims to support 8,000 social workers to return to the register.  **Supporting independence, people at the end of life, and responding to individual needs**  It is vital that this care is maintained as far as practically possible throughout the pandemic to protect the health and quality of life of this vulnerable group of individuals.  **Supporting local authorities and the providers of care**  … to strengthen the voice of people with lived experience, provide transparency, and help lessons to be learned. |

| COVID-19: how to work safely in care homes (8)  Published: 17/04/2020  Author:  Public Health England | **Aims:**  Guidance provided on the use of PPE for care staff working in care homes.... derived from, and should be read in conjunction with, full IPC and PPE guidance. Guidance applies to all care … and in circumstances of wandering residents and applies to all residents including those in the extremely vulnerable group. |
| --- | --- |
|  | **Recommendations:**  Guidance provides recommendations/recommended PPE:   - when providing personal care which requires a worker to be in direct contact with the resident(s) (e.g., touching) or within 2 metres of a resident who is coughing, - when performing a task requiring a worker to be within 2 metres of resident(s) but no direct contact with resident(s) (i.e., no touching), - when working in communal areas with residents- no direct contact with resident(s) though potentially within 2 metres of resident(s). - As there is sustained transmission of COVID-19 there is recommend use of PPE in general, in circumstances where no resident has symptoms of fever or cough and where no staff member or visitor has experienced these symptoms in the preceding 14 days, then PPE may not be required.   Guidance states that “where COVID-19 is circulating in the community at high rates, and symptoms can differ from person to it is not always obvious who might be infectious” and acknowledges that elderly residents often have minimal symptoms of respiratory infection. |
|  | **Implementation:**   - Provides links to wider PHE guidance on IPC. - PPE is only effective when combined with good hand hygiene, good respiratory hygiene and effective infection control practice. - Sustained transmission means that there are many people with COVID-19 infection who do not have a single recognisable contact with an individual who has had symptoms of infection. |
|  | **Intended outcomes:**  Not explicitly stated. |

| Coronavirus (COVID-19): support for care homes (9)  Published: 15/05/2020  Author:  [Department of Health and Social Care](https://www.gov.uk/government/organisations/department-of-health-and-social-care) | **Aims:**  Guidance focuses on how to prevent and control COVID-19 in all registered care homes...  …document sets out the steps that must now be taken to keep people in care homes safe, and the support that will be brought together across national and local government to help care providers put this into practice. |
| --- | --- |
|  | **Recommendations:**  **IPC**   - The NHS is offering support on training to care homes, with infection control nurses acting as “training the trainers” in care homes on the recommended approach to infection prevention control, PPE usage and testing advice.   **PPE**   - Distributing additional PPE to LRFs, for care homes with urgent shortages. - Training offered to the social care sector to ensure that PPE is being used safely and efficiently.   **Reducing workforce movement between care homes and minimising risk for care workers**   - Providers should take all possible steps to minimise staff movement between care homes, to stop infection spreading between locations. Subject to maintaining safe staffing levels, providers should employ staff to work at a single location.   **Quarantining**   - All patients being discharged to care homes will be tested prior to discharge. Local authorities should ensure that there is sufficient alternative accommodation as required to quarantine and isolate residents, if needed, before returning to their care home from hospital.   **Building our scientific understanding and sharing good practice across the sector**   - - Sub-group focusing specifically on the scientific issues affecting care homes and their residents established by SAGE.   - SCIE developing practical tools and a hub of best practice for care home staff.   **Stepping up NHS Clinical Support**   - Primary and community support to care homes, including introduction and use of key medical equipment, and to roll out video consultations within care homes.   **Comprehensive testing**   - Care homes now have access testing for all their residents and staff via a digital portal. Testing is available for care homes with a new outbreak, COVID-19 free care homes with over 50 beds and care homes referred by local authorities based on size (over 50 beds) and local knowledge.   **Oversight and compliance**   - Local authorities to review or put in place a care home support plan, drawing on local resilience and business continuity plans. - Increase compliance with the daily provider reporting requirements through capacity tracker.   **Building the workforce**   - Launch of a new national recruitment campaign.   **Funding**  Two tranches of extra funding to local authorities – £1.6 billion on 19 March 2020 and a further £1.6billion on 18 April 2020 – to support them in meeting pressures across the range of public services, along with £1.3billion via the NHS specifically to support safe and timely discharge from hospitals into care. |
|  | **Implementation:**  **IPC**   - Signposts to PHE guidance on preventing and controlling infections, including the use of PPE, isolation practices, and decontamination and cleaning processes.   **PPE**   - Testing and rolling out a PPE distribution portal to supplement normal supply chains to the care sector. - Coproduction of guidance for using PPE in social care settings.   **Reducing workforce movement between care homes and minimising risk for care workers**   - The infection control fund is intended to help providers pay for additional staff and/or maintain the normal wages of staff who, in order to reduce the spread of infection need to reduce the number of establishments in which they work, reduce the number of hours they work, or self-isolate.   **Quarantining**   - Costs of providing this accommodation are covered by the £1.3 billion COVID-19 discharge funding via the NHS.   **Stepping up NHS Clinical Support**  Provision of:   - - timely access to clinical advice, including a named clinical lead with weekly check-ins,   - proactive support for care home residents through personalised care and support,   - support for care home residents with suspected or confirmed COVID-19 through remote monitoring (face-to-face assessment where clinically appropriate),   - sensitive and collaborative decisions around hospital admissions for care home residents.   **Building the workforce**  Recruitment campaign supported by fast-track DBS checks for new recruits and volunteers, £3 million to support free rapid online training for new recruits, existing staff and volunteers, a new dedicated app for the adult social care workforce in England under the new CARE brand. |
|  | **Intended outcomes:**  …the government is working round the clock with industry, the NHS, social care providers and the armed forces to improve the supply of PPE. The fund will support adult social care providers to reduce the rate of transmission in and between care homes and support wider workforce resilience. |

| COVID-19: Adult Social Care Risk Reduction Framework: Assessing and reducing the risk to your workforce (10)  Published: 19/06/2020  Author:  [Department of Health and Social Care](https://web.archive.org/web/20200404122850/https:/www.gov.uk/government/organisations/department-of-health-and-social-care) | **Aims:**  Guidance developed in response to requests from employers and concerns that workers who are potentially more vulnerable to infection or adverse outcomes from COVID-19, may not be adequately supported. Guidance focuses specifically on how employers can support workers with factors which may make them more vulnerable to infection or adverse outcomes from COVID-19 to make decisions about their risks in the workplace. |
| --- | --- |
|  | **Recommendations:**  Intended to provide support to:   - Manage the process of undertaking risk assessments to identify those workers who may be more vulnerable to infection or experiencing significant health challenges as a result. - Have sensitive, one-to-one conversations with workers, to acknowledge concerns, discuss the options available and agree any next steps. - Identify and implement ways of mitigating the risk to these workers within the context of their role and the setting.   Employers are responsible for ensuring that risk assessments are carried out and steps are taken to minimise the risk to workers. If a worker is identified as being at higher risk, a one-to-one conversation should be used to identify how risk could be reduced, such as:   - Engaging the workforce and involving them in decision-making, solutions and support. - Giving workers opportunities to express concerns. - Reviewing the workplace following the working safely guidance. - Encourage people to follow the guidance on hand washing and hygiene. - Consideration of impacts of working pattern (including long shifts/night shifts, additional shifts or multiple jobs) on fatigue levels. - Supporting taking regular breaks, provide food and water while on shift. - Following DHSC and PHE advice on testing. - Providing clear information to all workers if PPE is or is not needed, when it should be used and ensuring all workers have adequate access in line with current guidance. - Redeployment to a setting or a role where the risks are lower- for example to support people who are not thought to be infected by COVID-19 or to work remotely where possible, as per the 5 steps to working safely guidance. - Adjustments to work arrangements   If the workplace is not safe for the employee and the employee cannot work from home or be redeployed, then employers could consider paid or unpaid leave until such time that it is safe for the employee to return to work. |
|  | **Implementation:**  The risk assessment process should be in two stages; identification of those who are potentially at higher risk and then assessing the risks associated with those individuals and identifying actions to minimise the risks. An initial identification process should take place with each worker to understand their potential individual risk. This could be having a conversation with each worker to identify if they have any potential risk factors and if a risk assessment is required, or requesting all workers complete a form or survey individually and have conversations only with those who have identified as having potential risk factors to undertake the risk assessment.  Guidance states that the identification and assessment process should not be a one-off process and should regularly review the assessment and management of risk to consider any new evidence on risk factors. Further guidance provided on having conversations with workers who are identified as high risk to complete a risk assessment, and steps that can be taken once workforce members who are at increased risk have been identified, including measures across the workforce and individual measures.  Provides links to guidance on IPC, PPE and supporting workers' health and wellbeing. |
|  | **Intended outcomes:**  Not explicitly stated. |

| Visiting arrangements in care homes (11)  Published: 22/07/2020  Author:  [Department of Health and Social Care](https://web.archive.org/web/20200404122850/https:/www.gov.uk/government/organisations/department-of-health-and-social-care) | **Aims:**  …our first priority continues to be to reduce the risk of COVID-19 transmission in care homes and prevent future outbreaks, to ensure the health and safety of both care workers and residents. Guidance estates that directors of public health and care providers should follow the guidance to ensure policies for visiting arrangements and decisions are based on a dynamic risk assessment and minimise risk wherever possible. |
| --- | --- |
|  | **Recommendations:**  Guidance focuses on five areas:  **1. Principles of a local approach and dynamic risk assessment**   - The first priority must remain preventing infections in care homes, and this means that visiting policy should still be restricted with alternatives sought wherever possible. However, as the rate of community transmission has reduced, care homes can now develop a policy for limited visits, following the advice set out in this guidance. - In the event of an outbreak in a care home and/or evidence of community hotspots or outbreaks leading to a local lockdown, care homes should rapidly impose visiting restrictions to protect vulnerable residents, staff and visitors.   **2. Advice for providers when establishing their visiting policy**   - Decisions on visiting policies require a risk assessment. - Care homes should support NHS Test and Trace by keeping a temporary record of current and previous residents, staff and visitors, as well as keeping track of visitor numbers and staff. - In the event of an outbreak in a care home and/or evidence of community hotspots or outbreaks, care homes may rapidly impose visiting restrictions to protect vulnerable residents, staff and visitors, setting out alternative options to maintain social contact for their residents while providing regular, personalised updates to residents’ loved ones.   **3. Advice for providers when taking visiting decisions for particular residents or groups of residents**   - Guidance provided in situations where a care home’s visiting policy allows for different rules to be applied to different residents. - In making these decisions, the care provider should actively involve the resident, their relatives or friends, any advocates etc.   **4. Infection control precautions**  Guidance provided regarding the precautions should be taken in respect of infection control during visits and their communication, including:   - Alternatives to in-person visiting should be actively encouraged, such as the use of telephones or video. - Visits should be limited to a single constant visitor, per resident, wherever possible, in order to limit the overall numbers of visitors to the care home and the consequent risk of infection. - Enabling a booking/appointment for visitors – ad hoc visits should not be enabled. - In line with test and trace guidance, providers should maintain a record of any visitors to a care home. - Visitors should have no contact with other residents and minimal contact with care home staff. - Visitors should follow IPC procedures, including hand washing, wearing a face covering and PPE if required. - All visitors should be screened for symptoms of acute respiratory infection before entering. - Consider whether visits could take place in a communal garden or outdoor area, or consider the use of plastic or glass barriers between residents and visitors. - Consider the possible use of designated visiting rooms. - Visitors should be encouraged to walk to the home or use their own transport. - Visitors should be encouraged to keep personal interaction with the resident to a minimum, for example avoid skin-to-skin contact (handshake, hug) and follow the latest social-distancing advice for as much of the visit as possible. - Discuss with visitors any items they wish to bring with them on their visit, such as a gift.   **5. Communicating with family and others about the visiting policy and visiting decisions** A visiting policy should be set out and shared with advice for residents and families, including:   - support for visitors on how to prepare for a visit. - provide reassurance to visitors, including that some people with dementia might struggle at first to remember or recognise them.   Friends and family should be advised that their ability to visit care homes is still being controlled, is based on a dynamic risk assessment, and is subject to the specific circumstances of the care home and those living and working within it. This is likely to mean that the frequency of visits is limited and/or controlled.  To limit risk, where visits do go ahead, this should be limited to a single constant visitor, per resident, wherever possible. This is in order to limit the overall numbers of visitors to the care home and the consequent risk of infection. |
|  | **Implementation:**  The process of considering visitors should be led by the relevant local director of public health, who should give a regular professional assessment of whether visiting is likely to be appropriate within their local authority, considering the wider risk environment. Routine awareness of this advised visiting guidance should be communicated to local Care Provider Associations, local commissioners of care homes, the clinical commissioning group infection-control lead and the PHE, local HPTs and the local outbreak board.  **1. Principles of a local approach and dynamic risk assessment**   - The approach is based on the circumstances and needs of the individual care setting including both residents and staff and the external COVID-19 environment. The external COVID-19 environment includes the prevalence and incidence of infection in the local community and/or outbreaks or hotspots which may increase risk of infection in visitors to care homes in the area. - The care home’s visiting policy should be made available and/or communicated to residents and families, together with any necessary variations to arrangements due to external events. Prior to visits being allowed in care homes, the director of public health in every area should disseminate their view on the suitability of visiting in the local authority area, considering infection rates and the wider risk environment. The decision on whether or not to allow visitors, and in what circumstances, is an operational decision and therefore ultimately for the provider and managers of each individual setting to make. Guidance provides considerations on the decision to allow visitors.   **2. Advice for providers when establishing their visiting policy**  Provides factors to be considered when making a risk assessment for a visiting policy.  **3. Advice for providers when taking visiting decisions for particular residents or groups of residents**  Signposting to the MCA and the ethical framework for adult social care. In making these decisions, the care provider should actively involve the resident, their relatives or friends, any advocates, commissioners and appropriate members of the multi-disciplinary team and, where appropriate, volunteers.  **5. Communicating with family and others about the visiting policy and visiting decisions** When considering their visiting policy, staff will need to consider the legal, decision-making framework offered by the MCA, individually for each of these residents. The government has published advice on caring for residents without relevant mental capacity - regard should be given to the ethical framework for adult social care. |
|  | **Intended outcomes:**  The first priority must remain preventing infections in care homes.  … to be to reduce the risk of COVID-19 transmission in care homes and prevent future outbreaks, to ensure the health and safety of both care workers and residents. |

| Personal protective equipment (PPE): illustrated guide for community and social care settings (12)  Published: 31/07/2020  Author:  UK Health Security Agency | **Aims:**  Guidance outlines PPE advice for social care workers working in the community. |
| --- | --- |
|  | **Recommendations:**  Guidance states that social care workers in the community should wear the correct PPE for their work. Guidance is shown on what PPE to wear for five scenarios for five scenarios:   1. Personal care involving touching the person being cared for, 2. Working within 2 metres of anyone who has a cough, 3. Within 2 metres of the individual being cared for, but not touching them, 4. When caring for a person who is shielding, 5. In the workplace and 2 metres away from people being cared for.   Wearing PPE for personal care of all individuals is recommended, whether the person receiving personal care to has symptoms or is known to have COVID-19 or not, regardless of the age of the person and no matter what the workers role is. |
|  | **Implementation:**  Links to further guidance, including ‘How to work safely in care homes’ and ‘Dementia in care homes and COVID-19’. |
|  | **Intended outcomes:**  Not explicitly stated. |

| Overview of adult social care guidance on coronavirus (COVID-19) (13)  Published: 25/08/2020  Author:  [Department of Health and Social Care](https://web.archive.org/web/20200404122850/https:/www.gov.uk/government/organisations/department-of-health-and-social-care) | **Aims:**  Provides information for adult social care providers about COVID-19 guidance and support. |
| --- | --- |
|  | **Recommendations:**  Guidance provides links to further guidance and support on key areas:  **Help with IPC** - guidance for social care providers on how to prevent, control and protect care workers and those they care for from COVID-19 infection.  **Supporting staff members at higher risk from COVID-19**  **Reducing contact between staff**  To help reduce possible transmission among staff and residents consider:   - limiting or ‘cohorting’ staff to groups of patients or floors/wings (segregation of COVID-19 positive and COVID-19 negative patients), - holding team meetings and handovers remotely, - staggering times of entry to collect equipment, including PPE, - access to regular remote supervision for teams and individuals, - remote, secure sharing of information relating to care between agencies.   **Running a medicines re-use scheme during the COVID-19 pandemic** – links to wider guidance  **Financial support for infection control and prevention measures**  Signposts to the Infection Control Fund for Adult Social Care (funding to support adult social care providers in England reduce the rate of transmission in and between care homes and to support workforce resilience).  **What to do when an outbreak is suspected**   - An outbreak is defined as 2 or more confirmed cases of COVID-19 or clinically suspected cases of COVID-19 among individuals associated with a specific setting, such as care homes, with onset dates within 14 days. - In line with the new definition, one confirmed case will be reported as an incident. If there’s a single laboratory confirmed case, this would initiate further investigation and risk assessment.   **What care homes and other social care settings must do during an outbreak**   1. Care home managers should contact their local HPT if they suspect an outbreak. HPTs will arrange the first tests for all residents and staff. Care homes should seek advice from their local HPT if they have a single possible case of COVID-19. 2. Staff should immediately instigate full infection control measures. 3. If the care home has a current outbreak and the steps for a new outbreak have been completed, they can continue regular weekly testing for staff. However, asymptomatic testing of residents is not needed unless recommended otherwise by the HPT or the Director of Public Health following a local risk assessment. If a resident develops symptoms in this time, the care home should contact the HPT to access rapid testing. Residents who have been exposed to a person with possible or confirmed COVID-19 should be isolated (or cohorted if not possible) with other similarly exposed residents until 14 days after last exposure. 4. Care home managers must postpone routine non-essential medical and other appointments, where possible, and discuss with the healthcare providers whether these could be delivered remotely. 5. During an outbreak, restrictions to visiting set out in the visiting guidance. 6. All staff and residents should be retested again 28 days after the last resident or staff had a positive test result or showed COVID-19-like symptoms. If no further cases are identified at this point, the outbreak is considered to have ended. Any further cases after this point is a new outbreak and the care home must contact the HPT. Staff or residents who have been diagnosed with COVID-19 should not be included in testing (as part of regular testing or the whole home test at 28 days after the last identified case) until 6 weeks after their initial onset of symptoms, or if staff or residents were asymptomatic when tested, until 6 weeks after their positive test result. If they develop new symptoms, they should be retested immediately.   All staff and residents should be retested again 28 days after the last resident or staff had a positive test result or showed COVID-19-like symptoms. If no further cases are identified at this point, the outbreak is considered to have ended. Any further cases after this point is a new outbreak and the care home must contact the HPT.  **Reporting an outbreak**   1. Care homes should record clinically suspected or confirmed cases in staff or residents daily onto the Capacity Tracker... This is important in notifying HPTs of a potential outbreak and notifying of recovery from an outbreak. 2. Report any outbreak to the local HPT, they will provide advice and support to manage the outbreak. Once an outbreak is confirmed, the HPT will arrange testing for all residents and staff. The HPT will also arrange a follow up test after 4 to 7 days for residents and staff who tested negative on the first round of testing or who missed the initial test. 3. If a new case is identified, follow HPT advice and undertake a risk assessment to see if all communal activities should be stopped. 4. The HPT may advise that restrictions be implemented for 28 days. 5. The outbreak can be declared over once no new cases have occurred in the 28 days since the onset of symptoms in the most recent case.   **How to manage staff during an outbreak**  Staff who have COVID-19 symptoms should not attend work, notify their line manager immediately and self-isolate for 10 days, following the guidance for household isolation and guidance on management of exposed healthcare workers.  **Advice on when symptomatic staff can return to work**  Symptomatic staff can return to work no earlier than 10 days from symptom onset, provided clinical improvement has occurred and they have been afebrile (not feverish) without medication for 48 hours and they’re medically fit to return.  **Support for those struggling to manage during an outbreak**  All care providers should contact their local authority and local health services for support during an outbreak. If local authorities are unable to meet the emergency needs of a care provider, they should report to their Strategic Co-ordination Group or LRFs for additional support.  **Caring for patients discharged from hospital or another social care facility**   - Any person who does not need an NHS hospital bed will continue to be discharged in line with the current discharge service requirements. - Admission and care of people in care homes’ guidance sets out a service model for testing people moving from the community into a care home. It is intended to supplement existing local arrangements. The care home should isolate new residents coming from the community for a 14-day period following admission. - Local authorities should ensure that sufficient alternative accommodation is available to quarantine and isolate residents, if needed, before returning to their care home from hospital, as set out in the adult social care action plan.   **Visits to care homes and other care settings –** refers to wider guidance.  **How to get social care workers and people in care homes tested**   - In England, all registered adult care homes can apply for COVID-19 tests, through their local HPT. - HPTs will provide advice and arrange the testing for all residents and staff. Care homes can continue weekly testing for staff if in a current outbreak, but it is not recommended to continue asymptomatic testing of residents, unless otherwise suggested by the HPT or Director of Public Health following a local risk assessment. - Repeat testing from 6 July 2020 with weekly testing of staff and testing of residents every 28 days in care homes without outbreaks. To access retesting, care homes will need to register on the portal for retesting. - Initially repeat testing is available for care homes for over-65s and those with dementia, expanding to mixed and specialist care homes shortly. - Care homes that do not have a suspected or current outbreak can continue regular testing and follow the whole home retesting cycle, applying for testing kits via the testing portal.   **Managing care workers during COVID-19**   - In order to support staff to return to work as soon as it is safe to do so, testing has been made available to all social care workers.   Includes further guidance on financial support and information for businesses and providers, furlough, redeploying workers and using volunteers to maintain staffing levels, recruiting and training new worker and restricting workforce movement to minimise transmission.  **Securing PPE and related supplies**  Providers contact their business-as-usual supplier where possible. If care homes are unable to obtain PPE through business-as-usual suppliers or one of the designated wholesalers, there are three emergency routes providers can use to access PPE; PPE portal, LRFs and the NSDR System.  **Information for social care providers on mental health and wellbeing and financial support**  Includes support for social care workers relating to mental health, resources and training for registered managers, wellbeing and bereavement support for social care workers and advice for employers and those who are self-employed. Also includes guidance on taking time off to care for a relative who has COVID-19 symptoms or is self-isolating, financial support for employers of social care workers and steps to take following a COVID-19 related death of a person who worked in adult social care. |
|  | **Implementation:**  **Help with IPC**  Links to guidance for care home workers; how to work safely in care homes and all social care settings and PPE illustrated guide.  **Supporting staff members at higher risk from COVID-19**  Guidance provided on the adult social care risk reduction framework, PHE guidance on IPC, guidance on safe working and DHSC guidance on health and wellbeing of the adult social care workforce.  **Reducing contact between staff**  Signposts to guidance on reducing workforce movement between care homes and minimising risk for care workers.  **What to do when an outbreak is suspected**  Guidance on implementing, care home admission, social distancing measures and shielding guidance. Also, guidance on how to work safely in care homes, admission and care of residents in a care visiting and advice on what can and can’t be done in an outbreak.  **How to manage staff during an outbreak**  Links to guidance on household isolation, guidance on management of exposed healthcare workers, guidance on restricting workforce movement and minimising workforce transmission, and advice on when symptomatic staff can return to work.  **Support if care homes struggling to manage during an outbreak** Signposting to the action plan for adult social care and the care home support package for support on how to manage during an outbreak.  **Caring for patients discharged from hospital or another social care facility**  References current discharge service requirements and advice on how to safely admit residents being discharged from hospitals to care homes. ‘Admission and care of people in care homes’ guidance sets out a service model for testing people moving from the community into a care home. Local authorities should ensure that sufficient alternative accommodation is available to quarantine and isolate residents, if needed, before returning to their care home from hospital, as set out in the adult social care action plan.  **Visits to care homes and other care settings**  Signposts to guidance for providers on arrangements for visitors in care homes.  **How to get social care workers and people in care homes tested**  Guidance on admission and care, social distancing measures and COVID-19 testing.  **Managing care workers during COVID-19**  Signposts to guidance on the management of staff and patients or residents in health and social care settings according to exposures, symptoms and test results. Includes further guidance on financial support and information for businesses and providers, furlough, redeploying workers and using volunteers to maintain staffing levels, recruiting and training new worker and restricting workforce movement to minimise transmission.  **Securing PPE and related supplies**  Provides links to tailored resources on how to work safely in care homes published by PHE.  **Information for social care providers on mental health and wellbeing and financial support**  Provides links to Every Mind Matters, Shout, Samaritans and Hospice UK. Includes links to skills for Care has created a package of support for registered managers and the CARE-branded website and app containing guidance and practical support for the social care workforce. Links to guidance on health and wellbeing, including local carers support organisation through Carers UK, and charities such as the Care Workers’ Charity. Workers should talk to their employer about their caring needs and what arrangements can be put in place. Information regarding statutory sick pay entitlements can be found in the guidance for employees. Also provides guidance on using the Capacity Tracker, and the COVID-19 ethical framework for adult social care. |
|  | **Intended outcomes:**  … how to prevent, control and protect care workers and those they care for from COVID-19 infection. |

| Adult social care: our COVID-19 winter plan 2020 to 2021 (14)  Published: 18/09/2020  Author:  [Department of Health and Social Care](https://web.archive.org/web/20200404122850/https:/www.gov.uk/government/organisations/department-of-health-and-social-care) | **Aims:**  Guidance sets out the key elements of national support available for the social care sector for winter 2020 to 2021. The government’s three overarching priorities for adult social care are:   - ensuring everyone who needs care or support can get high-quality, timely and safe care throughout the autumn and winter period, - protecting people who need care, support or safeguards, the social care workforce, and carers from infections including COVID-19, - making sure that people who need care, support or safeguards remain connected to essential, services and their loved ones whilst protecting individuals from infections including COVID-19.   Guidance aims to set out our approach to supporting the adult social care sector by detailing what the government’s national support will be establishing expectations of other parts of the system, including local authorities, NHS organisations, and care providers. Guidance will put into practice the recommendations of the Social Care Sector COVID-19 Support Taskforce, providing a stimulus for further local winter planning and preparedness. |
| --- | --- |
|  | **Recommendations:**  **Preventing and controlling the spread of infection in care settings**  **Guidance on infection prevention and outbreak management** Actions for local authorities and NHS organisations:   - continue to implement relevant guidance and circulate and promote guidance to adult social care providers in their area, including for visitors, - directors of public health should work with relevant partners including PHE and local health protection boards to control local outbreaks and should refer to the contain framework, - support care homes, working with local partners to carry out learning reviews after each outbreak to identify and share any lessons learned at local, regional and national levels.   Actions for providers:   - continue to follow all relevant guidance on how to prevent, control and manage an infection in their care setting, - undertake a learning review after an outbreak, with support from their local authority, - liaise fully with their PHE local HPT and other local partners as needed, - report shortfalls in support or equipment, to local authorities, and through established data capture portals, - ensure all care staff have ongoing training on IPC, the appropriate use of PPE, and can engage with online training for learning reviews when available. The CQC’s registration requirements support this approach during the COVID-19 pandemic in line with the relevant guidance and code of practice, - ensure staff are carrying out all relevant IPC procedures to a high standard through a robust system of audit and quality assurance, - identify and support an individual to be the lead for IPC for COVID-19 to ensure adherence to infection prevention guidance, - ensure staff do not work if they have COVID-19 symptoms, or a member of their household has symptoms or a recent positive test, or they have been told to isolate by NHS Test and Trace.   **Managing staff movement,**  National support:   - continue to support all providers to put in place measures to stop staff movement, - continue to support providers to pay staff who are self-isolating, in line with government guidance, their normal wages while doing so.   Actions for local authorities and NHS organisations:   - distribute money from the Infection Control Fund, and submit returns on how the funding has been used in line with the grant conditions, - consult the guidance available on redeploying staff and managing their movement, and support providers in their area to access other initiatives, - continue to review contingency arrangements to help manage staffing shortages, within social care provision, through the winter, with the aim of reducing the need for staff movement, - provide clear communication to social care providers regarding the importance of implementing workforce measures to limit COVID-19 infection, signpost relevant guidance, and encourage providers to make use of additional funding where appropriate, - actively monitor Capacity Tracker data to identify and act on emerging concerns regarding staff movement between care settings, including following up with care providers who are not limiting staff movement.   Actions for providers:   - Care home providers should limit all staff movement between settings unless absolutely necessary to help reduce the spread of infection, including by reviewing exclusivity arrangements with recruitment agencies, and considering the recruitment of additional staff over the winter period, - All care providers should use funding from the Infection Control Fund to meet additional costs associated with restricting workforce movement for infection control purposes, in accordance with the conditions on which it is given by local authorities.   **PPE** National support:  To continue to ensure that there is sustainable supply of PPE (as recommended by COVID-19 PPE guidance) to social care over the winter period:   - providing free PPE for COVID-19 needs (as recommended by COVID-19 PPE guidance), to care homes and domiciliary care providers via the PPE portal until March 2021. This will supplement the PPE available for their business-as-usual needs through their usual suppliers, - providing LRFs with buffer stock of free PPE to create local stockpiles which can be used to provide emergency supplies for all care providers in case of local COVID-19 spikes, - providing LRFs (those who continue with PPE distribution) with free PPE for supplying the COVID-19 needs of social care providers who are not eligible for the portal, - providing local authorities (in those areas where LRFs are ceasing PPE distribution) with free PPE for supplying social care providers who are not eligible for the portal, - publishing a PPE strategy shortly which will set out winter and longer-term plans in greater detail.   Actions for local authorities and NHS organisations:   - provide PPE for COVID-19 when required, either through the LRF (if in an area where they are continuing PPE distribution), or directly to providers (if in an area where the LRF has ceased distribution) report shortages to the LRF or to DHSC.   Actions for providers:   - follow all relevant guidance on use of PPE, including recommendations for those providing support to people with learning disabilities or autistic people, - make use of free government-funded PPE stocks where needed and in line with COVID-19 PPE guidance. Care homes and domiciliary care providers, along with some others, are eligible to register for the PPE portal guidance and can obtain free PPE through this route. Providers ineligible to register for the portal (for example, personal assistants), should obtain PPE from their LRF (if it is continuing to distribute PPE), or their local authority, - in the event of urgent need for PPE stocks, use the NSDR or contact their LRF to access free emergency supply from the LRF stockpile, - report shortages via Capacity Tracker and/or the CQC community care survey.   **COVID-19 testing**  National support:  Links to testing strategy for adult social care.  Actions for local authorities and NHS organisations:  Local authorities should:   - ensure positive cases are identified promptly, make sure care providers, as far as possible, carry out testing as per the testing strategy and, together with NHS organisations, provide local support for testing in adult social care, if needed, - actively monitor their local testing data to identify and act on emerging concerns, including following up with care homes that are not undertaking regular testing, as per the guidance.   NHS organisations should:   - continue to test people being discharged from hospital to a care home.   PHE HPTs should:   - continue to deliver their testing responsibilities, as outlined in the testing strategy. This includes continuing to arrange testing for outbreaks in care homes and other adult social care settings, as appropriate and follow the local outbreak plan as directed by their Director of Public Health. A risk-based testing regime should be implemented appropriate for the area, seeking advice from the National COVID-19 Response Centre as needed, - advise care homes on outbreak testing and IPC measures, - in an outbreak area, refer to the COVID-19 Contain Framework.   Actions for providers:  All providers should:   - ensure all staff are aware of how to access symptomatic testing, - contact their local HPT if they suspect an outbreak within their service.   All care homes should additionally ensure that they are following the guidance regarding regular testing of staff and residents.  **Seasonal flu vaccines**  National support:  Lists the groups provided with a free flu vaccine.  Actions for local authorities and NHS organisations:  Local authorities should:   - support communications campaigns encouraging eligible staff and people who receive care to receive a free flu vaccine, - direct providers to local vaccination venues, - work with local NHS partners to facilitate and encourage the delivery of flu vaccines to social care staff and residents in care homes.   Actions for providers:  All care providers should:   - support and promote, to all staff, the importance of receiving a free flu vaccination, - proactively encourage and enable staff to receive a free flu vaccination, either by providing vaccines in the workplace, through an occupational health scheme, direct arrangement or with at a community pharmacy or general practice, - report their staff flu vaccination rate via the Skills for Care Adult Social Care Workforce Data Set and flu vaccination status for staff and residents in the Capacity Tracker.   Care home providers should additionally:   - take all reasonable steps to support residents to receive a flu vaccination through the NHS scheme or occupational health schemes, where in place. Pharmacists can now vaccinate staff and residents at the same time.   **Collaboration across health and care services Safe discharge from NHS settings and preventing avoidable admissions**  National support:  The government has agreed to fund, via the NHS:   - the cost of post-discharge recovery and support services, such as rehabilitation and reablement, for up to a maximum of 6 weeks, in all settings, - urgent community response services for people who would otherwise be admitted into hospital. These services will typically provide urgent support, within 2 hours, and for a limited time (typically 48 hours) and, if required, transition into other ongoing care and support pathways, - A comprehensive care and health assessment for any ongoing care needs, including determining funding eligibility, should take place during the first 6 weeks, in a community setting. CHC assessments should resume, from September 2020, and assessments deferred due to COVID-19 should be completed as rapidly as possible. - Where a person is discharged from hospital into a care home, they will continue to be tested prior to discharge. Results should be communicated to the receiving provider. Working up a designation scheme with CQC for premises that are safe for people leaving hospital who have tested positive for COVID-19 or are awaiting a test result. No provider should be forced to admit an existing or new resident if they are unable to cope with the impact of the person’s COVID-19 illness safely. Local authorities remain responsible for providing alternative accommodation in local systems.   Actions for local authorities and NHS organisations:  Local authorities and CCGs should work together to:   - jointly commission care packages for those discharged (including commissioning of care home beds). The local authority should be the lead commissioner unless otherwise agreed between the CCG and the local authority, - establish an Executive Lead for the leadership and delivery of the discharge to assess model, - establish efficient processes to manage CHC assessments in line with the guidance on the reintroduction of NHS CHC (as well as the discharge guidance), which includes extending the use of the Trusted Assessor Model and digital assessments, - secure sufficient staff to rapidly complete deferred assessments, drawing on discharge funding but without negatively impacting on care home support, - work with partners to coordinate activity, with local and national voluntary sector organisations, to provide services and support to people requiring support around discharge from hospital and subsequent recovery.   Hospital clinical and leadership teams should additionally ensure COVID-19 testing of all people being discharged from hospital to a care home. COVID-19 test results should always be communicated to the care home before the individual leaves the hospital and be included in documentation that accompanies the person on discharge. Care homes have a right to refuse admission to residents and should not accept admissions if they cannot safely cohort or isolate them. Where possible hospitals should plan 48 hours in advance of discharge to ensure test results are available and care homes have a chance to plan for a timely discharge.  Local authorities additionally:   - are required to provide appropriate accommodation for people who have been discharged from hospital, if their care home cannot provide appropriate isolation or cohorting facilities, as set out in the Adult Social Care Action Plan. Every local authority should work with their respective CCG, to ensure that they have safe accommodation for people who have been discharged from hospital with a positive or inconclusive COVID-19 test result. Discharge funding has been made available via the NHS to cover the costs of providing alternative accommodation. - should consider adopting the cohorting and zoning recommendations published by ADASS, working with providers. This should include ensuring early partnership discussions with providers, about the safety and feasibility of implementing these arrangements within their care homes.   Actions for providers:  Care home providers should accept people discharged from hospital only when able to do so safely. They should:   - isolate all residents discharged from hospital or interim care facilities for 14 days regardless ofCOVID-19 test result, unless they have already undergone isolation for a 14-day period in another setting, in line with the admission and care of people in care homes guidance, - alert their local authority if they have been requested to receive an individual from hospital with a confirmed COVID-19 positive test result, - advise their local authority if they assess they cannot accept an individual from hospital with a confirmed COVID-19 positive test results as they are unable to safely isolate or cohort the individual, or if they cannot manage the needs of the individual due to other challenges impacts on infection control capability (such as staffing issues), - alert their local HPT in the event of positive COVID-19 test results, - continue to update the Capacity Tracker daily or more frequently if something changes,   Other care providers should:   - work with adult social care contract leads to identify capacity that can be used for hospital discharge purposes or follow-on care from reablement services, - work collaboratively with NHS primary and community care services.   **Enhanced health in care homes**  National support:  PCNs – working with community healthcare providers –will become responsible for delivering the Enhanced Health in Care Homes (EHCH) framework. This includes:   - timely access to clinical advice for care home staff and residents, including a named clinical lead from the PCN, for every care home, and weekly MDT support, - support for care home residents with suspected or confirmed COVID-19 through remote monitoring (and face-to-face assessment where clinically appropriate).   Wider support to care homes includes:   - pulse oximeters available to care homes that do not have the recommended number of devices (1 per 25 beds) which, used under clinical supervision, can help identify ‘silent hypoxia’ and rapid deterioration of people with COVID-19, - rehabilitation for those recovering from COVID-19, provided by both primary and community healthcare services, - training and development for care home staff, - support with data, IT and technology, including access to care records and secure email.   Actions for local authorities and NHS organisations:   - Guidance provided for CCGs and PCNs.   Actions for providers:  Care home providers should:   - familiarise themselves with the enhanced health in care homes service requirements and what they can expect from NHS agencies, - work collaboratively with clinical leads to delivery optimum care and support to their residents, - work with the local CCG to determine local need for oximeters.   **Technology and digital support** National support:  To continue to support the sector over winter:   - enable further deployment of NHSmail and Microsoft Teams for all care providers, - offer discounted broadband deals for care home providers, allowing care homes to improve their internet connections and access video consultations for residents and better enable connections to loved ones, - provide regularly updated support materials on the Digital Social Care website, - to support care providers to introduce new technologies, - distribute tablet devices to care homes that are in greatest need, so that care home staff can access remote health consultations for the people in their care. This will also support care home residents to stay connected with their families and loved ones. Technical and user support will be provided to set up the devices for use by care providers. - accelerate the safe adoption of products that enable care providers to access GP record information for people within their care, including the ability to reorder medications online on behalf of their residents. - publish a new version of the data security and protection toolkit for the care sector, with additional guidance and support to enable safe use of technologies.   Actions for local authorities and NHS organisations:  CCGs should:   - continue to support all care providers in their local area to enable NHSmail, collaboration tools and remote consultations for people receiving social care in all settings. - work with local authorities to support eligible care homes in their local area to apply for a tablet device as part of the NHSX offer. - have active conversations about whether appropriate local data-sharing agreements are in place between health and social care provider settings. - alert the NHSX Information Governance team if issues with sharing information are identified.   Actions for providers:  All care providers are asked to:   - consider how technical or digital solutions may help them to protect the people they support from COVID-19 and connect them to their loved ones, - engage with the NHSX DSPT support offer and register with DSPT by 30 September 2020, - alert the NHSX Information Governance team if any advice is required.   Care homes are advised to:   - take advantage of time-limited connectivity deals and complete the simple order form to apply for a tablet device.   **Acute hospital admissions** National support:   - Emergency admissions will continue to be determined by clinical decision making.   Actions for local authorities and NHS organisations:  NHS settings should:   - take a risk-based approach to routine admissions for elective care and advise patients about appropriate testing and/or isolation requirements pre-admission.   Actions for providers:  All care providers should:   - work together with NHS partners to address issues in order to reduce unnecessary emergency admissions.   If needed, care home providers should:   - support residents to self-isolate prior to admission for an elective care procedure.   **Supporting people who receive social care, the workforce, and carers Supporting independence and quality of life Visiting guidance** National support:   - published visiting guidance, - whilst most areas with high prevalence are implementing restrictions, for avoidance of doubt, in any area listed by PHE as an ‘area of intervention’, the Director of Public Health should immediately move to stop visiting except in exceptional circumstances such ascend of life.   Actions for local authorities and NHS organisations:  Directors of Public Health should:   - give a regular assessment of whether visiting care homes is likely to be appropriate, within their local authority, or within local wards, considering the wider risk environment, - if necessary, impose visiting restrictions if local incidence rates are rising, and immediately if an area is listed as ‘an area of intervention’, - In all cases exemptions should be made for visits to residents at the end of their lives.   Actions for providers:  Care home providers should:   - develop a policy for limited visits (if appropriate), in line with up-to-date guidance from the irrelevant Director of Public Health and based on dynamic risk assessments which consider the vulnerability of residents. This should include both whether their residents’ needs make them particularly clinically vulnerable to COVID-19 and whether their residents’ needs make visits particularly important. Social workers can assist with individual risk assessments, for visits, and can advise on decision-making where the person in question lacks capacity to make the decision themselves. - set out the precautions that will be taken to prevent infection during visits and ensure these are communicated in a clear and accessible way, - ensure the appropriate PPE is always worn and used correctly – which in this situation is inappropriate form of protective face covering (this may include a surgical face mask where specific care needs align to close contact care) and good hand hygiene for all visitors, - limit visitors to a single constant visitor wherever possible, with an absolute maximum of two constant visitors per resident to limit risk of disease transmission, - supervise visitors at all times to ensure that social distancing and IPC measures are adhered to, - wherever possible visits should take place outside, or in a well-ventilated room, for example with windows and doors open where safe to do so, - immediately cease visiting if advised by their respective director of public health that it is unsafe,   **End-of-life care** Any advance care decision, including Do Not Attempt Cardio-Pulmonary Resuscitation (DNACPR) decisions, should be fully discussed with the individual and their family, where possible and appropriate, and should be signed by the clinician responsible for their care. It is unacceptable for advance care plans, including DNACPR decisions, to be applied in a blanket fashion to any group of people.  National support:   - end-of-life care, including advance care planning must always be personalised. Ensuring that the system is fully aware of the importance of delivering personalised approaches to care planning, advance decisions and care delivery, and that best practice guidance is available to support this in all care settings. Guidance can be found on the NHS England website and the Resuscitation Council UK website. - In light of anecdotal reports of inappropriate practice in applying DNACPRs. This is unacceptable and there has been national action, across a number of fronts, to prevent this from happening. Continuing to work with stakeholders to understand what can be done nationally to prevent inappropriate DNACPR decisions being made for individuals. - Guidance for the public setting out what a DNACPR decision is, how it should be applied, who should be involved and what to do if an individual or their loved ones have concerns will be published by NHS England and Improvement shortly, - The CQC will urgently raise cases of inappropriate use of DNACPR as it becomes aware with the relevant bodies, including the General Medical Council, and act where registered providers are responsible.   Actions for local authorities and NHS organisations:  NHS organisations and local authorities should:   - ensure that discussions and decisions on advanced care planning, including end of life, should take place between the individual (and those people who are important to them where appropriate) and the multi-professional care team supporting them. Where a person lacks the capacity to make treatment decisions, a care plan should be developed following where applicable the best interest checklist under the MCA. - implement relevant guidance and circulate, promote and summarise guidance to the relevant providers. This should draw on the wide range of resources that have been made available to the social care sector by key health and care system partners and organisations including those on the NHS website and those published by the Royal Colleges of GPs. - All organisations should put in place resources and support to ensure that wherever practicable and safe loved ones should be afforded the opportunity to be with a dying person, particularly in the last hours of life.   Actions for providers:  All care providers should:   - have regard to the above guidance in delivering personalised approaches to care. This is applicable to all providers of end-of-life care in all scenarios, including where care is being provided in an individual’s home, - ensure they make every effort, wherever practicable and safe, to enable a dying person to be with their loved ones, particularly in the last hours of life, - If a care provider is concerned about pressures to put in place DNACPRs, they should escalate, in the first instance, using their internal whistleblowing policies.   **Supporting the workforce, including staff training** National support:   - continuing to offer rapid online induction training to help induct and train redeployed staff, new starters, existing staff and new volunteers in social care services. This training is provided free of charge when accessed through Skills for Care, meaning staff can be trained quickly ahead of winter, - guidance aimed at supporting the workforce has been published earlier this year, including guidance for carers of people with a learning disability and autistic adults’ advice on supporting people with dementia living in care homes, and guidance for supported living, - developing support to help those people working in social care who are delivering delegated health tasks that were previously undertaken by staff in community health services.   Actions for local authorities and NHS organisations:  Local authorities should:   - ensure providers are aware of the free induction training offer and encourage them to make use of it, - promote and summarise relevant guidance to care providers.   Actions for providers:  All care providers should:   - engage with the training offer and support staff to undertake training, as soon as possible, - support staff to access guidance relevant to the needs of people in their care.   **Supporting the wellbeing of the workforce** National support:   - made available, to staff, national resources and guidance, including guidance providing, - advice on how staff can manage their personal mental health in light of the current pandemic. This also provides employers with guidance, tools and advice on how to take care of the wellbeing of staff at work. - The Chief Social Workers have issued guidance, in partnership with the Tavistock and Portman NHS Trust, for the support and wellbeing of adult social workers and social care professionals, - ‘Our Frontline’, a collaboration between Samaritans, Shout, Hospice UK and Mind, provides information, emotional support and access to a crisis text service. The Samaritans and Hospice UK have also extended their support lines to provide support to social care staff. - All care workers have free access to several mobile apps to support their mental health and wellbeing, until at least December 2020. These include Daylight, Sleepio and Silvercloud. - A package of support for Registered Managers is available, recognising that they are facing particular challenges. This includes a series of webinars and a dedicated advice line. - Our offer to the workforce is brought together in the CARE workforce app, which signposts to resources, in one place, which will be available until at least December 2020. - Work with local authorities to assess access to occupational health provision and other wellbeing support available to social care staff, highlight good practice and consider where improvements can be made before the end of November 2020.   Actions for local authorities and NHS organisations:  Local authorities should:   - maintain, where possible, the additional staff support services which they put in place during the first wave of the pandemic, - review current occupational health provision with providers in their area and highlight good practice, - promote wellbeing offers to their staff and allow staff time to access support, as well as promoting to providers in their area.   Actions for providers:  All care providers should:   - reinforce the message that staff wellbeing remains of the utmost priority. There are tips and advice on how employers can take care of the wellbeing of staff at work in our guidance on the health and wellbeing of the adult social care workforce. - Continue to promote the CARE workforce app and other available resources to support their employees. - Undertake a workplace risk assessment, with a view to protecting the health, safety and welfare of all staff. Employers should have individual conversations, about COVID-19, with all members of their workforce who may be at increased risk. A risk reduction framework for adult social care has been published to provide guidance, for employers, on how to sensitively to discuss and manage specific risks to their staff – this includes risk by ethnicity, but also age, sex and underlying health conditions. This guidance will be reviewed as new evidence emerges.   **Workforce capacity** National support:  To support providers to maintain staffing over the winter period, working with the care sector to monitor and respond to vacancy and absence levels including working with:   - the Department for Work and Pensions to ensure that those who have lost their jobs duringCOVID-19 are aware of and encouraged to take up opportunities in social care. - Skills for Care to ensure rapid response induction training remains available and to share guidance and resources to help providers recruit the staff they need - use our communication channels to support recruitment into the adult social care workforce. - In addition, setting up a short-term workforce planning group to identify and implement further ways to help address workforce capacity issues which could occur over the winter period. This could include the development of staff banks and other local initiatives. Continuing to work with the Bringing Back Staff initiative to help social care providers access nurses and other professionals who sign up to be redeployed. - issued guidance on redeploying staff and using volunteers to support the sector to build capacity where most needed the NHS Volunteer Responders programme is available to the social care sector. They can be a helping hand for those clinically vulnerable to COVID-19, or any other reason support is needed during the pandemic and will continue to be so, at least until the end of March 2021. - The legislative, vetting, regulatory, and pay and conditions frameworks that have been put in place will continue to enable the temporary registration of returning staff and students to build capacity in the health and care workforce throughout the winter period. - The extension of the Infection Control Fund can be used by providers to help cover the costs of ensuring that staff who are isolating in line with government guidance can safely stay at home.   Actions for local authorities and NHS organisations:  Local authorities should:   - continue to review contingency arrangements to help manage staffing shortages within social care provision through the winter. - Consult the guidance available on deploying staff and managing their movement, and support providers in their area to access other initiatives – for example Bringing Back Staff. - Consider how voluntary groups can support provision and link-up care providers with the voluntary sector where necessary. - Support providers, in their area, to complete the capacity tracker and update their adult social care workforce data set records to help ensure effective local capacity monitoring and planning.   Actions for providers:  All care providers should:   - ensure they have read the section in this plan on managing staff movement. - Put arrangements in place to ensure they have sufficient staff to provide safe, high-quality care even in the event of increased staff absence. - Consider recruiting to fill vacancies via the Department for Work and Pensions ‘Find a Job’ website, as well as using local recruitment methods. Free recruitment campaign materials are available on our campaign website. - Continue to ensure staff receive normal wages while self-isolating in line with government guidance. The government has provided funding to support this through the Infection Control Fund. - Consider how volunteers could help support service delivery and link-up with the NHS Volunteer Responder’s programme and the wider voluntary sector. - Ensure they complete the capacity tracker and update their records to ensure effective planning for local, regional and national capacity issues.   **Shielding and people who are** **clinically extremely vulnerable** National support:  If shielding advice is reintroduced in a local area, writing to all clinically extremely vulnerable people, in that area, with further advice; and the local authority will coordinate support in that area.  Actions for local authorities and NHS organisations:   - Local authorities will coordinate local support if shielding is reintroduced in a local area. This includes provision of enhanced care and support for clinically extremely vulnerable people on the shielded persons list.   Actions for providers:  All care providers should:   - complete a workplace risk assessment to secure the health, safety and welfare of all staff, - have individual conversations, about COVID-19, with all members of the workforce who are clinically extremely vulnerable, or are otherwise identified as being at an increased risk, before a return to work or a return to their previous role, - follow government advice on safe working to take the maximum steps to ensure the safety of everyone in their workplace.   **Supporting the system Funding** National support  The extension of the Infection Control Fund will:   - continue to support care home providers to ensure staff only work in one setting, - continue to support providers to pay staff who are isolating in line with government guidance receive their normal wages while doing so, - provide further support to other settings, beyond care homes, such as domiciliary care and other community settings.   Actions for local authorities  Local authorities should:   - provide DHSC with information about how the money Infection Control Fund has been spent by 30 September 2020, - continue to maintain the information they have published on their websites about the financial support they have offered to their local adult social care market, - provide regular returns to DHSC on the spending of the extended Infection Control Fund in line with the grant conditions.   Actions for providers  All providers should:   - spend the initial Infection Control Fund by the end of September 2020, in accordance with the grant determination letter, - provide data through the Capacity Tracker, or through other relevant data collection or escalation routes in line with government guidance and the conditions of the Infection Control Fund, - implement the recommended IPC measures, - provide information to local authorities about spending supported by the Infection Control Funding line with the grant conditions, - maintain robust financial records about their use of the Infection Control Fund.   Further guidance provided on direct payments, support for unpaid carers, Care Act easements, social work and other professional leadership, social prescribing, market and provider sustainability, emergency support framework and sharing best practice and Care home support plans. |
|  | **Implementation:**  Links to the adult social care action plan, the care home support package, and the Infection Control Fund and other guidance throughout the document as stated in the main recommendations section. For each recommendation, information is provided on key actions for national support, for local authorities and NHS organisations and social care providers, including in the voluntary and community sector. |
|  | **Intended outcomes:**  …. ensure that high-quality, safe and timely care is provided to everyone who needs it, whilst protecting people who need care, their careers and the social care workforce from COVID-19.  … ensure the sector is prepared for winter, protecting people who need care and the workforce that supports them.  Managing staff movement: Stopping staff movement in and between care settings is critical to minimise the risk of infection ofCOVID-19 and other viral illnesses, including flu.  COVID-19 testing: Testing is a critical part of supporting adult social care in the fight against COVID-19 and helps to prevent and control the spread of infection and outbreaks by breaking the chain of transmission. Through the government’s testing programme, committed to delivering the testing needed to limit the spread of the virus and to save lives.  Visiting guidance: Our first priority remains to prevent infections in care homes and protect staff and residents. |

| About the Adult Social Care Infection Control Fund (15)  Published: 21/09/2020  Author:  [Department of Health and Social Care](https://web.archive.org/web/20200404122850/https:/www.gov.uk/government/organisations/department-of-health-and-social-care) | **Aims:**  Guidance prepared in response to a number of questions received from local government and care providers regarding the £600 million Adult Social Care Infection Control Fund.  The primary purpose of this fund is to support adult social care providers to reduce the rate of COVID-19 transmission in and between care homes and support wider workforce resilience. |
| --- | --- |
|  | **Recommendations:**  The funding will be available in two tranches, the first has been paid to local authorities on 22 May 2020 and the second tranche in early July 2020.  **Infection control measures**  Care homes are expected to take additional steps to reduce the spread of infection as detailed in annex C of the grant circular. Local authorities must ensure that 75% of the grant is allocated to support the following measures in respect of care homes:   - ensuring that staff who are isolating in line with government guidance receive their normal wages while doing so. - Ensuring that members of staff work in only one care home, including staff who work for one provider across several homes or staff that work on a part-time basis for multiple employers and includes agency staff. - Limiting or cohorting staff to individual groups of residents or floors/wings, including segregation of COVID-19 positive residents, for example paying for extra staff cover to provide the necessary level of care and support to residents, or paying for structural/ physical changes to support separation of floors/wings and/or residents. - Supporting active recruitment of additional staff (and volunteers) if they’re needed to enable staff to work in only one care home or to work only with an assigned group of residents or only in specified areas of a care home, including by using and paying for staff who have chosen to temporarily return to practice, including those returning through the NHS returners programme. - Steps to limit the use of public transport by members of staff. - Providing accommodation for staff who proactively choose to stay separately from their families in order to limit social interaction outside work. |
|  | **Implementation:**  Guidance includes specific restrictions on the use of funding, requirements on local authorities and requirements on providers included throughout the guidance. |
|  | **Intended outcomes:**  … funding is to support specific measures for reducing the rate of transmission in and between care homes, such as minimising the movement of staff to reduce the risk of symptomatic transmission of the virus and ensuring staff do not lose out on wages in doing so. |

| Adult Social Care Infection Control Fund: round 2 (16)  Published:  01/10/2020  Author:  Department of Health and Social Care | **Aims:**  The purpose of this fund is to support adult social care providers, including those with whom the local authority does not have a contract, to reduce the rate of COVID-19 transmission within and between care settings, in particular by helping to reduce the need for staff movements between sites. |
| --- | --- |
|  | **Recommendations:**  All funding must be used for COVID-19 infection control measures. Local authorities should pass 80% of this funding to care homes, including residential drug and alcohol services, and CQC-regulated community care providers within the local authority’s geographical area. The other 20% of the funding must be used to support care providers to take additional steps to tackle the risk of COVID-19 infections but can be allocated at the local authority’s discretion.  **IPC measures**  Providers can use this funding to pay for the continuation of infection control measures they may have already taken if they are in line with these measures:  Residential settings:   - - ensuring that staff who are isolating in line with government guidance receive their normal wages and do not lose income while doing so. At the time of issuing the grant circular, this includes:   - staff with suspected symptoms of COVID-19 waiting for a test,   - where a member of the staff’s household has suspected symptoms of COVID-19 and are waiting for a test,   - where a member of the staff’s household has tested positive for COVID-19 and is therefore self-isolating,   - any staff member for a period of at least 10 days following a positive test,   - if a member of staff is required to quarantine prior to receiving certain NHS procedures (generally people do not need to self-isolate prior to a procedure or surgery unless their consultant or care team specifically asks them to).   - Limiting all staff movement between settings unless absolutely necessary, to help reduce the spread of infection. This includes staff who work for one provider across several care homes, staff that work on a part-time basis for multiple employers in multiple care homes or other care settings (for example in primary or community care). This includes agency staff (the principle being that the fewer locations that members of staff work in the better). Where the use of agency staff is absolutely necessary, this should be by block booking.   - Limiting or cohorting staff to individual groups of residents or floors/wings, including segregation of COVID-19 positive residents.   - To support active recruitment of additional staff (and volunteers) if they’re needed to enable staff to work in only one care home or to work only with an assigned group of residents or only in specified areas of a care home, including by using and paying for staff who have chosen to temporarily return to practice, including those returning through the NHS returners programme. These staff can provide vital additional support to homes and underpin effective infection control while permanent staff are isolating or recovering from COVID-19.   - Steps to limit the use of public transport by members of staff (considering current government guidance on the safe use of other types of transport by members of staff.   - Providing accommodation for staff who proactively choose to stay separate from their families in order to limit social interaction outside work.   - Supporting safe visiting in care homes, such as dedicated staff to support and facilitate visits, additional IPC cleaning in between visits, and capital-based alterations to allow safe visiting such as altering a dedicated space.   - Ensuring that staff who need to attend work for the purposes of being tested (or potentially in the future, vaccinated) for COVID-19 are paid their usual wages to do so, and any costs associated with reaching a testing facility.   **About the 20% allocation for other care settings and infection control measures**  A non-exhaustive list of wider measures that this could include is below:   - providing support on the IPC measures outlined above to a broader range of care settings, including, but not limited to: - community and day support services (the department would like local authorities to consider using this fund to put in place IPC (IPC) measures to support the resumption of services), - other non-CQC regulated residential settings, - carers support services, - individuals who directly employ one or more personal assistants to meet their care needs, - individuals who are in receipt of direct payments, - the voluntary sector, - Paying care staff their usual wages in order to attend a GP or pharmacy to be vaccinated against flu outside of their normal working hours. - Measures the local authority could put in place to boost the resilience and supply of the adult social care workforce in their area to support effective infection control.   **Specific restrictions on the use of the funding**  **Staff who are off sick with conditions other than COVID-19, furloughed or shielding**  This funding cannot be used by providers to pay usual wages to staff who are off sick with conditions other than COVID-19, nor to top up the pay of staff who are furloughed or to pay the wages of staff who may be shielding (in line with government guidance). This funding can be used to pay usual wages of staff who are self-isolating with suspected COVID-19 symptoms (rather than only after a positive test), but those individuals must be seeking to confirm whether this is COVID-19 through a test.  **PPE**  Local authorities may use 20% of the grant on other COVID-19 infection control measures to support the care sector. This could include, for example, additional financial support for the purchase of PPE by providers or by the local authority directly (although not for costs already incurred).  **Visiting**  Providers should only use this funding to put in place extra measures to facilitate safe visiting (in line with government guidance) due to the risk of transmission of COVID-19.  **Interaction with Test and Trace**  The Infection Control Fund provides financial support to providers so they can continue to pay their staff their normal wages whilst self-isolating according to government guidelines. The fund aims to ensure that care workers do not lose income because they are self-isolating. |
|  | **Implementation:**  Links back to the Adult Social Care Infection Fund, introduced May 2020. The funding will be paid in 2 tranches. The first will be paid to local authorities on 1 October 2020. The second tranche will be paid in December 2020. It is expected that the grant will be fully spent on infection control measures by 31 March 2021.  Links to guidance on use of public transport and the test and trace scheme. Links to the Adult Social Care Winter Plan the government’s commitment to the provision of free PPE for COVID-19 needs for adult social care providers until March 2021. Guidance includes requirements for local authorities and requirements for providers.  Annexe provides a non-exhaustive list of examples of ways in which providers can spend funding as part of the ‘per beds’ or ‘per user’ allocation. |
|  | **Intended outcomes:**  … to reduce the rate of COVID-19 transmission within and between care settings, in particular by helping to reduce the need for staff movements between sites.  … supporting providers with the additional costs they will face in complying with the government guidance on IPC with respect to COVID-19, particularly workforce measures that restrict staff movement. |

| Designated settings for people discharged to a care home (17)  Published: 16/12/2020  Author:  [Department of Health and Social Care](https://www.gov.uk/government/organisations/department-of-health-and-social-care), [UK Health Security Agency](https://www.gov.uk/government/organisations/uk-health-security-agency), [Care Quality Commission](https://www.gov.uk/government/organisations/care-quality-commission), and [NHS England](https://www.gov.uk/government/organisations/nhs-england) | **Aims:**  Guidance developed to support safe and timely discharge and protect care home residents and staff from COVID-19 throughout winter. Document provides guidance in the delivery of the designation scheme, for local authorities, clinical commissioning groups, care providers and people who use these services. |
| --- | --- |
|  | **Recommendations:**   - Discharges from hospital should follow the principle of ‘Home First’ and only a small proportion of people who are in hospital will be discharged to care homes.   **The new requirement**   - Every patient must receive a COVID-19 PCR test result within 48 hours prior to discharge. No one will be discharged into, or back into, a registered care home setting without being tested, and having received their test result. Separate policy applies for individuals who have tested COVID-19 positive within 90 days of illness onset. - Everyone with a COVID-19 positive test result being discharged into or back into a registered care home setting should first be discharged into a designated setting. Designated settings should have the additional policies, procedures, equipment, staffing and training in place to maintain infection control and have the capability to support the care needs of residents as set out in CQC’s IPC protocol. This is an important precaution to protect care home residents and minimise, where possible, the risk of infection. - People should undergo a 14-day period of isolation before moving into a care home from a designated setting, whether that be hospital or local authority commissioned - the total 14-day period of isolation can be shared across two designated settings if IPC practices are not breached. Subject to the care home provider’s decision and a clinical assessment to determine if the individual is likely to be infectious, residents will not have to undergo a further period of isolation. - These designated premises will need to have undergone an inspection by CQC to assure that they meet the latest CQC infection prevention control standards, as set out in CQC’s IPC protocol. The results of these inspections will be posted on the CQC website as per usual practice. - Everyone being discharged into a care home must have a time-stamped reported COVID-19 test result, and this must be communicated to the person themselves and the care home prior to the person being discharged from hospital. The care home’s registered manager should continue to assure themselves that all its admissions or readmissions are consistent with this requirement. Local authorities should ensure they are involved in the discharge process for anyone being admitted to a care home for the first time. - Local authorities must ensure that they have sufficient designated settings available. This is vital to minimise transmission of COVID-19 and protect the lives of those living and working in care homes. If a local authority does not have any ‘switched on’ designated settings available, either due to lack of CQC assurance, local authority nominated beds, or assured beds not being ‘switched on’, the local authority must take rapid steps to ensure designated settings are nominated and assured. - In exceptional circumstances where designated setting arrangements are not yet ‘switched on’ in the local area, current discharge arrangements for COVID-19 positive individuals, as set out in the existing discharge guidance and admissions to care home guidance, should continue to apply. This includes the requirement to provide a test result prior to discharge and notify the person’s COVID-19 status to the person themselves, unpaid carers, and care providers. Nobody should be discharged to a care home without a test result.   **Locating and designating settings**  It is important that sufficient settings are located and designated as quickly as possible to meet potential demand across England over winter. It is intended for every local authority to have access to at least one designated setting or suitable alternative premises (for example, NHS community hospital beds). Local authorities will also be able to identify more than one facility to be CQC assured if needed, to respond to geographical spread and size, and to consider the specific needs of particular people and increasing demands.  **COVID-19 testing prior to discharge to a care home**   - NHS provider organisations must ensure all people being discharged into care homes have received a COVID-19 test within the preceding 48 hours of the discharge date. - All individuals who test positive for COVID-19 within 48 hours of being discharged into a care home should be discharged into a designated setting in the first instance. - Those individuals who test positive for COVID-19 in a hospital setting within 48 hours of being discharged into a care home should undergo a 14-day isolation period before moving into the care home. The total 14-day isolation period can be shared across the hospital and a designated setting if IPC practices are not breached.   **Discharge of people who have tested positive for COVID-19**  Designated settings are being established within each health and social care system to support the safe ongoing care and isolation of people who have tested positive for COVID-19 and are not returning home on discharge. These settings will be used for those who would otherwise be returning to the care home from where they were admitted, or for the small proportion of individuals who are unable to go home and therefore being discharged to a care home for the first time.  If a local authority does not have any ‘switched on’ designated settings available, either due to lack of CQC assurance, local authority nominated beds, or assured beds not being ‘switched on’, the local authority must take rapid steps to ensure designated settings are nominated and assured. In exceptional circumstances where designated setting arrangements are not yet ‘switched on’ in the local area, individuals should be discharged according to current discharge arrangements for COVID-19 positive individuals.   - All residents should be discharged to a designated setting in the first instance. Designated settings are being established within each health and social care system to support the safe ongoing care and isolation of people who have tested positive for COVID-19 and are not returning home on discharge. These settings will be used for those who would otherwise be returning to the care home from where they were admitted, or for the small proportion of individuals who are unable to go home and therefore being discharged to a care home for the first time. - NHS provider organisations must ensure all people being discharged into care homes have received a COVID-19 test within the preceding 48 hours of the discharge date. All individuals who test positive for COVID-19 within 48 hours of being discharged into a care home should be discharged into a designated setting in the first instance. Those individuals who test positive for COVID-19 in a hospital setting within 48 hours of being discharged into a care home should undergo a 14-day isolation period before moving into the care home. - People who have been discharged to a designated facility will remain in this setting for 14 days, or the remainder of their isolation period, after discharge. At the end of this isolation period, the person can move to their usual or new place of residence in another care setting without the need for a further COVID-19 test or further isolation. - In relation to confirmed COVID-19 positive cases, no care home will be forced to admit an existing or new resident to the care home if they are unable to cope with the impact of the person’s COVID-19 illness. - If appropriate isolation or cohorted care is not available with a local care provider, the individual’s local authority will be required to secure alternative appropriate accommodation and care for the remainder of the required isolation period. - In exceptional circumstances where designated setting arrangements are not yet ‘switched on’ in the local area, individuals should be discharged according to current discharge arrangements for COVID-19 positive individuals, as set out in the existing discharge guidance. |
|  | **Implementation:**  Links to adult social care winter plan, the hospital discharge service guidance, testing and visiting. The designation scheme is intended for those:   - who are leaving hospital and require care within a CQC-registered care home for the first time, or are returning to an existing placement, and - have tested positive for COVID-19 within the 48 hours preceding their discharge from hospital and/or those who are within an appropriate formal isolation period having tested positive for disease. For those entering care homes this would be 14 days in line with the care homes admission guidance.   **Locating and designating settings**   - Further guidance provided on the type of facility required, and expectations to be met in terms of infection prevention control and clinical support.   **Managing designated setting capacity**   - Local authorities must ensure that sufficient settings are available to meet expected needs now and over the winter period. The costs of the designated facilities will be met through the £588 million discharge funding.   Further guidance provided on for discharge managers, and further guidance on exemptions on clinical or care grounds, and support for people without relevant mental capacity, support for care providers and providers of designated settings, and information collection and governance and funding.  Everyone being discharged into a care home must have a time-stamped reported COVID-19 test result, and this must be communicated to the person themselves and the care home prior to the person being discharged from hospital. The care home’s registered manager should continue to assure themselves that all its admissions or readmissions are consistent with this requirement. Local authorities should ensure they are involved in the discharge process for anyone being admitted to a care home for the first time.  NHS provider organisations must ensure that COVID-19 test results for all people being discharged into a care home are received and shared with the individual themselves, their key relatives or advocates, and the relevant care home provider prior to discharge taking place. NHS medical staff and ward managers are responsible for ensuring that COVID-19 test results are available before discharge and are shared with individuals and receiving care homes. |
|  | **Intended outcomes:**  …ensures that everyone being discharged from hospitals to care homes who is COVID-19 positive, is discharged to premises that meet a set of agreed control standards to complete the recommended 14-day isolation period.  … to minimise transmission of COVID-19 and protect the lives of those living and working in care homes.  To further support safe and timely discharge and protect care home residents and staff from COVID-19 throughout winter… |

| Adult Social Care Rapid Testing Fund: guidance (18)  Published: 15/01/2021  Author:  [Department of Health and Social Care](https://web.archive.org/web/20200404122850/https:/www.gov.uk/government/organisations/department-of-health-and-social-care) | **Aims:**  The main purpose of this funding is to support additional rapid testing of staff in care homes, and to support visiting professionals and enable indoors, close contact visiting where possible. |
| --- | --- |
|  | **Recommendations:**  Funding will provide help to protect residents and workers from COVID-19 and will be paid in a single instalment in January 2021. This grant can be used to cover expenditure from 2 December 2020 to 31 March 2021. Funding must be used to support increased lateral flow device (LFD) testing in care settings.  **Measures that this funding can be spent on**   - Paying for staff costs associated with training and carrying out LFD testing, including time taken to: - attend webinars, read online guidance and complete an online competency assessment, - explain the full LFD testing process to those being tested, and ensuring that they understand all other IPC measures, - ensure that any LFD tests are completed properly, including overseeing the self-swabbing process, processing tests and logging results, - wait for results, if staff are coming in to undertake tests on site prior to their shift, - welcoming visitors, gaining consent and overseeing that PPE is correctly donned. - Costs associated with recruiting staff to facilitate increased testing, - Costs associated with the creation of a separate testing area where staff and visitors can be tested and wait for their result. This includes the cost of reduced occupancy where this is required to convert a bedroom into a testing area, but only if this is the only option available to the care home, - Costs associated with disposal of LFD tests and testing equipment.   Local authorities must use 20% of the funding to support the care sector to operationally deliver LFD testing, but this can be allocated at the local authority’s discretion. This could include:   - supporting care homes or other providers that are currently experiencing an outbreak to ensure that they have the resources needed to administer the LFD tests and equipment that they need to increase LFD testing, - supporting smaller homes to implement LFD testing as they may face relatively higher costs compared to large homes, - supporting other settings such as supported living and extra care settings eligible for LFD testing.   Funding is separate to the Infection Control Fund and the extension to the Infection Control Fund. |
|  | **Implementation:**   - Signposts to guidance on visitor testing in care homes and guidance on the use of LFD tests for visitors and visiting professionals in care homes. - Local authorities should pass 80% of this funding to care homes within the local authority’s geographical area on a ‘per beds’ basis. The remaining 20% of the funding must be used to support the care sector to implement increased LFD testing but can be allocated at the local authority’s discretion. - Care homes in receipt of LFD tests are required to register the results of all tests as per the care home testing guidance for staff and residents or visitors and visiting professionals. - Guidance includes requirements for local authorities and providers. |
|  | **Intended outcomes:**  … to support additional rapid testing of staff in care homes, and to support visiting professionals and enable indoors, close contact visiting where possible. |

| Your care home during winter (19)  Published: 22/01/2021– earliest version identified  Author:  [Department of Health and Social Care](https://web.archive.org/web/20200404122850/https:/www.gov.uk/government/organisations/department-of-health-and-social-care) | **Aims:**  Not explicitly stated. |
| --- | --- |
|  | **Recommendations:**  **IPC measures**   - It is essential that good infection control is maintained, and guidance is followed at all times, including on the journey to work and during breaks. Transmission can happen at any time, including when making colleagues drinks or sharing lifts. - PPE must be worn, and guidance followed, regardless of whether supporting people with COVID-19 or not. - Wear PPE properly, change it carefully and stay stocked up.   **Testing**   - Routine and regular testing is a crucial part of keeping care homes safe. - Residents continue to be tested monthly.   **Visiting**   - Visits to care homes can take place with arrangements such as substantial screens, visiting pods, or behind windows. - End-of-life visits should always be supported.   **Wellbeing**  There is a range of support available to support all levels of concerns:   - social care staff who may be struggling to cope and need help can send a text message with ‘FRONTLINE’ to 85258 to start a conversation. This service is offered by Shout and is free on all major mobile networks. - The Samaritans confidential staff support line is open for all social care staff who might be feeling increasingly stressed, anxious or overwhelmed. - Hospice UK’s bereavement and trauma line is open for all social care staff. |
|  | **Implementation:**  Provides links to guidance on COVID-19 vaccination. |
|  | **Intended outcomes:**  Not explicitly stated. |

| Workforce Capacity Fund for adult social care (20)  Published: 29/01/2021  Author:  [Department of Health and Social Care](https://web.archive.org/web/20200404122850/https:/www.gov.uk/government/organisations/department-of-health-and-social-care) | **Aims:**  The purpose of this funding is to enable local authorities to deliver measures to supplement and strengthen adult social care staff capacity to ensure that safe and continuous care is achieved. |
| --- | --- |
|  | **Recommendations:**  The purpose of this funding is to enable local authorities to deliver measures to supplement and strengthen adult social care staff capacity to ensure that safe and continuous care is achieved to deliver the following outcomes:   - maintain care provision and continuity of care for recipients where pressing workforce shortages may put this at risk. - support providers to restrict staff movement between care homes and other care settings in all but exceptional circumstances. which is critical for managing the risk of outbreaks and infection in care homes. - support safe and timely hospital discharges to a range of care environments including domiciliary care, to prevent or address delays as a result of workforce shortages. - enable care providers to care for new service users where need arises.   **Identifying and implementing measures to increase workforce capacity**  Guidance sets out a range of tried and tested initiatives which other local authorities and care providers have successfully implemented to increase capacity. These examples can be used to address staffing shortages to ensure continuity of care and support, and to support providers to restrict staff movement between care homes and other settings in all but exceptional circumstances, by for example, supporting providers to access additional staffing resource to minimise deployment of those who work in multiple settings.  Guidance provides examples on how the funding can be used increase workforce capacity, including to address staffing shortages, to support, and to support providers to restrict staff movement between care homes and other settings, and supporting providers to access additional staffing resource to minimise deployment of those who work in multiple settings. Examples include:   - Establishing or expanding local authority staff banks, - Supporting administrative tasks so experienced and skilled staff can focus on providing care, - Redeployment of existing staff into new roles. This would cover local authority employed staff as well as staff from other services – for example, closed day services, - Local recruitment initiatives, - Funding new training costs for new recruits or to increase productivity of existing staff.   Other types of workforce capacity measures discussed, including incentivising take-up of additional hours, generating new supply of workers into social care and free resources to support recruitment and redeployment. |
|  | **Implementation:**  This is a ring-fenced grant of £120 million that will be paid in two instalments to local authorities, the first instalment will be paid in early February 2021, the second instalment worth £36 million (30%) will be paid in March 2021.  The department's expectation is that the grant will be fully spent on staffing capacity measures by 31 March 2021. The funding must be used to deliver new or additional measures which support the purpose of the fund, or the funding can be used to increase the scale of activities which already deliver additional workforce capacity where these exist within the local authority or providers.  Provides links to the adult social care COVID-19 winter plan, Skills for Care Area Teams, Local Care Providers Associations, the Care and Health Improvement Programme led by the Local Government Association, Association of Directors of Adult Services and the Care Provider Alliance.  Guidance includes reporting requirements and financial management. |
|  | **Intended outcomes:**  …restriction of routine staff movement remains critical to managing the risk of outbreaks and infection in care homes.  … emergence of a new and highly transmissible variant of COVID-19 has resulted in increased staffing shortages due to staff testing positive for COVID-19 or having to self-isolate.  …some people being discharged from hospital may require complex or increased social care as they recover from COVID-19 and other illnesses. |

| Restricting workforce movement between care homes and other care settings (21)  Published: 01/03/2021  Author:  [Department of Health and Social Care](https://web.archive.org/web/20200404122850/https:/www.gov.uk/government/organisations/department-of-health-and-social-care) | **Aims:**  Guidance sets out expectations of providers on limiting the routine movement of staff, and how to manage the risks of deploying individuals who work in multiple settings in those exceptional circumstances where it is the only remaining mechanism to ensure enough staff are available to care for service users safely. |
| --- | --- |
|  | **Recommendations:**  **Restricting routine staff movement**   - To reduce the risk of infections and outbreaks in care homes, providers should not deploy staff to provide nursing care or personal care if those individuals are also providing a regulated activity in another setting. This is for both permanent and temporary staff, including agency and bank staff, staff who work across multiple sites for the same employer and staff who work for more than one health and social care provider. - Staffing requirements should be planned so routine movement is not necessary to maintain safe staffing levels. Mitigations such as block booking should be used to further minimise staff movement where agency or other temporary staff are needed. - Should a provider need to deploy an individual who usually attends 2 settings, the provider should ensure there is a 10-day interval between the individual attending the 2 settings and require a negative test result prior to the individual entering the home. - Where a provider uses agency or other temporary workers, it should take steps to ensure those agency workers have not attended another health or social care setting in the previous 10 days. For example, providers could consider using block bookings and exclusivity contracts with agencies to limit agency staff movement. - Restrictions on staff movement should also apply to care homes that have been assured by the CQC to operate as a designated setting. Designated settings require a separate staff team in order to operate, and staff movement should be limited unless absolutely necessary, in accordance with this guidance. Where designated settings are a zoned setting within a care home (and not a ‘stand-alone unit’) providers should also ensure staff, movement is limited between designated and non-designated areas in line with this guidance on restricting staff movement between care homes and other settings. - While the safety of residents and staff is paramount, providers should, where possible, limit or ‘cohort’ staff to individual groups of residents or floors/wings, including segregation of COVID-positive and COVID-negative residents. - Providers should take steps to limit use of public transport by members of staff. Staff should avoid lift-sharing arrangements and travel solo in their own vehicle where possible.   Guidance provided on allowing staff movement in exceptional circumstances and further guidance on testing. Providers deploying staff working in multiple locations in exceptional circumstances should ensure this is for as limited a period of time as possible and should only be for as long as is needed for the provider to resolve any staffing issues. |
|  | **Implementation:**   - Links to winter plan for social care, which confirmed that stopping staff movement in and between care settings is critical to minimise the risk of infection of COVID-19. Care home providers should continue to limit all staff movement between settings unless absolutely necessary to help reduce the spread of infection. - Links to Infection Control Fund supports adult social care providers to reduce the rate of COVID-19 transmission within and between care settings. This includes compensating staff whose normal hours are reduced due to restrictions on their movement. - Links to Workforce Capacity Fund 120 million fund for local authorities to support staffing capacity for provision of adult social care in their areas, used to support providers to restrict staff movement by, for example, supporting providers to access additional staffing resource to minimise deployment of those who work in multiple settings. - Links to designated settings guidance, care home COVID-19 testing guidance for testing of staff and residents. - Includes incentivising the take-up of additional hours of existing social care staff by using the funding to, for example, pay overtime rates to encourage staff to work shifts additional to their usual or contracted hours, or cover childcare costs to allow staff to take on hours they would usually be unable to work due to child caring responsibilities. |
|  | **Intended outcomes:**  … reduce the risk of infections and outbreaks in care homes. |

| Coronavirus (COVID-19) testing available for adult social care in England (22)  Published: 24/03/2021 – earliest version identified July 2021  Author:  UK Health Security Agency | **Aims:**  Not explicitly stated. |
| --- | --- |
|  | **Recommendations:**  **Regular testing cycle for care home residents and staff:**   - Staff testing: Weekly polymerase chain reaction (PCR) and twice weekly LFT (one of the LFT on the same day as their PCR). - Resident testing: Every 28 days with PCR.   **Outbreak testing for care home residents and staff**   - Outbreak defined as two or more clinically suspected or confirmed positives (LFT or PCR) among residents or staff detected in the same 14-day period. Two or more cases therefore usually requires outbreak testing, HPT to advise. - One or more clinically suspected or confirmed cases in staff or residents: Immediately contact the local HPT for advice and start rapid response daily staff testing. Report situation to HPT for risk assessment and ask if outbreak testing required. - Outbreak testing: PCR tests for all staff and residents on day 1 of the outbreak and once between days 4-7, start of daily LFT testing of all staff until there has not been a positive result in 5 days. - Determining the end of outbreak testing: All staff and residents should be tested with PCR 14 days after the last resident or staff had a positive test result or showed COVID-19-like symptoms. Specific guidance given for variants of concern. If there are no positive PCR results from this “end of outbreak testing”, the HPT will declare the outbreak over.   **Family and Friend Visitors**  All family and friends’ visitors to care homes should be tested each time they visit with an LFT   - If a visitor receives a negative result, visits can proceed as long as visitors wear appropriate PPE throughout the visit and follow all IPC measures. - If a visitor receives a positive result, they should conduct a confirmatory PCR and register this at the care home using the ‘home’ registration route. The visitor should then go home to self-isolate immediately to await their result.   **Visiting professionals** (professionals who visit care homes as part of delivering their role, including health professionals, CQC inspectors and maintenance workers)   - The default position is that without a negative test, the professional should not be allowed into the care home. - NHS staff should be tested twice a week using LFT through their employer, via NHS Testing. - CQC Inspectors should be tested using LFT on the day of their visit to a care home, in addition to their current weekly PCR testing.   All visiting professionals who are not regularly tested through another route should be tested on the day of their visit, similar to visitors. |
|  | **Implementation:**  Includes links to ordering testing kits, antibody testing and further information. |
|  | **Intended outcomes:**  Not explicitly stated. |

| Adult Social Care Infection Control and Testing Fund (23)  Published: 29/03/2021  Author:  Department of Health and Social Care | **Aims:**  The purpose of this fund is to support adult social care providers, including those with whom the local authority does not have a contract, to:   - reduce the rate of COVID-19 transmission within and between care settings through effective IPC practices and increase uptake of staff vaccination, - conduct rapid testing of staff and visitors in care homes, high risk supported living and extra care settings, to enable close contact visiting where possible. |
| --- | --- |
|  | **Recommendations:**  Funding consolidates two distinct allocations of funding; the IPC fund and the rapid testing fund.  **IPC funding**  Local authorities should pass 70% of this funding to care homes, including residential drug and alcohol services, and CQC-regulated community care providers within the local authority’s geographical area. Local authorities must assure themselves that all direct funding for providers from this allocation is spent on the following IPC measures. Providers can use this funding to pay for the continuation of IPC measures they may have already taken if they are in line with these measures:  **Care homes (including residential drug and alcohol settings):**   - ensuring that staff who are isolating in line with government guidance receive their normal wages and do not lose income while doing so. At the time of issuing the grant circular, this includes: - staff with suspected symptoms of COVID-19 waiting for a test, - where a member of the staff’s household has suspected symptoms of COVID-19 and are waiting for a test, - where a member of the staff’s household has tested positive for COVID-19 and is therefore self-isolating, - any staff member for a period of at least 10 days following a positive test, - if a member of staff is required to quarantine prior to receiving certain NHS procedures (generally people do not need to self-isolate prior to a procedure or surgery unless their consultant or care team specifically asks them to). - limiting all staff movement between settings unless absolutely necessary, to help reduce the spread of infection. This includes staff who work for one provider across several care homes, staff that work on a part-time basis for multiple employers in multiple care homes or other care settings (for example in primary or community care). This includes agency staff. Mitigations such as block booking should be used to further minimise staff movement where agency or other temporary staff are needed. - limiting or cohorting staff to individual groups of residents or floors/wings, including segregation of COVID-19 positive residents. - to support active recruitment of additional staff (and volunteers) if they’re needed to enable staff to work in only one care home or to work only with an assigned group of residents or only in specified areas of a care home, including by using and paying for staff who have chosen to temporarily return to practice, including those returning through the NHS returners programme. These staff can provide vital additional support to homes and underpin effective IPC while permanent staff are isolating or recovering from COVID-19 steps to limit the use of public transport by members of staff (considering current government guidance on the safe use of other types of transport by members of staff). - providing accommodation for staff who proactively choose to stay separate from their families in order to limit social interaction outside work. - costs of PCR testing; including ensuring that staff who need to attend work or another location for the purposes of being tested for COVID-19 are paid their usual wages to do so, any costs associated with reaching a testing facility, and any reasonable administrative costs associated with organising and recording outcomes of COVID-19 tests. - costs of vaccination; including ensuring that staff who need to attend work or another location for the purposes of being vaccinated for COVID-19 are paid their usual wages to do so, any costs associated with reaching a vaccination facility, and any reasonable administrative costs associated with organising COVID-19 vaccinations where these were not being supported by other government funding streams.   **Rapid testing funding**  Care homes can use this funding to pay for the continuation of measures that they may have already taken if they are in line with the below:   - paying for staff costs associated with training and carrying out lateral flow testing, including time to: - attend webinars, read online guidance and complete an online competency assessment, - explain the full rapid LFT process to those being tested, and ensuring that they understand all other IPC measures, - ensure that any rapid LFTs are completed properly, including overseeing the self-swabbing process, processing tests and logging results, - wait for results, if staff are taking tests prior to their shift. - Supporting safe visiting, including: - welcoming visitors, - gaining consent to conduct lateral flow testing, - overseeing that PPE is correctly donned, - additional IPC cleaning in between visits; and - alterations to allow safe visiting such as altering a dedicated space. - Costs associated with recruiting staff to facilitate increased testing. - Costs associated with the maintenance of a separate testing area where staff and visitors can be tested and wait for their result. This includes the cost of reduced occupancy where this is required to convert a bedroom into a testing area, but only if this is the only option available to the setting. We expect that most costs will have been covered by the first Rapid Testing Fund. - Costs associated with disposal of LFTs and testing equipment.   **Local authority discretionary funding**  **IPC measures:**  A non-exhaustive list of wider measures that the funding could be used for is below:   - providing additional support to care homes or other providers that are currently experiencing an outbreak to ensure that they are able to put in place sufficient IPC measures, - providing support on the IPC measures outlined above to a broader range of care settings, including, but not limited to: - community and day support services, - carers support services, - individuals who directly employ one or more personal assistants to meet their care needs, - individuals who are in receipt of direct payments, - the voluntary sector, - measures the local authority could put in place to boost the resilience and supply of the adult social care workforce in their area to support effective IPC.   **Rapid testing measures:**  Local authorities to use their discretionary portion of the rapid testing allocation to support:   - supported living and extra care settings eligible for LFTs, - care homes or other providers that are currently experiencing an outbreak to ensure that they have the resources needed to administer the LFTs and equipment that they need to increase lateral flow testing, - smaller homes to implement lateral flow testing as they may face relatively higher costs compared to large homes, - other parts of the sector with lateral flow testing in line with any further rollouts.   **Specific restrictions on the use of the funding**  **Staff who are off sick with conditions other than COVID-19, furloughed or shielding**  This funding cannot be used by providers to pay usual wages to staff who are off sick with conditions other than COVID-19, nor to top up the pay of staff who are furloughed or to pay the wages of staff who may be shielding (in line with government guidance). This funding can be used to pay usual wages of staff who are self-isolating with suspected COVID-19 symptoms (rather than only after a positive test), but those individuals must be seeking to confirm whether this is COVID-19  through a test. In these circumstances, where a member of staff receives a negative test for COVID, a provider can still use this fund to pay usual wages where the symptoms were suspected to be COVID-19 in line with government guidance.  **PPE**  Local authorities may use their 30% discretionary portion of the IPC allocation on other COVID-19 IPC measures to support the care sector. This could include, for example, additional financial support for the purchase of PPE by providers or by the local authority directly (although not for costs already incurred), however it I expected that the PPE portal to be the first port of call.  **Designated settings**  To prevent the risk of infections entering care homes, anyone who is likely to be infectious with COVID-19 should be discharged to a designated setting, a facility that meets a set of agreed standards to specifically provide safe care for COVID-19 positive residents. The department is providing an additional £594 million through the hospital discharge programme to ensure that patients who have tested positive for the virus to be discharged safely from hospital into a specifically designated setting where they will receive appropriate care in a COVID-19-secure environment, before returning or moving into a care home or other care environment to prevent the spread of COVID-19.  Therefore, the Infection Control and Testing Fund should only be used for the IPC and rapid testing measures outlined. Any additional costs incurred by a designated setting to reach the standards to provide safe care for COVID-19 positive residents should be met from the hospital discharge programme.  **Visiting**  Where funding is spent on supporting visiting, this must be limited to measures that relate to managing the risks of COVID-19 transmission through visiting – in line with government guidance. Funding must not be spent on generic visiting facilities.  **Interaction with Test and Trace**  The Infection Control and Testing Fund provides financial support to providers so they can continue to pay their staff their full wages while they are self-isolating according to government guidelines on COVID-19. The fund aims to ensure that care workers do not lose income because they are self-isolating.  Infection Control and Testing Fund to be the primary way to support social care workers who need to stay at home and self-isolate. If an individual is receiving their full wage from their employer through the Infection Control and Testing Fund, they will not be eligible for the Test and Trace Support Payment scheme. |
|  | **Implementation:**  The funding will be paid to local authorities in April 2021…expect the grant to be fully spent on IPC and rapid testing measures by 30 June 2021. Signposts to guidance on public transport, testing service for extra care and supported living settings, designated settings and test and trace payments guidance.  Care providers in receipt of LFTs are required to register the results of all tests asper the care home COVID-19 testing guidance for regular and outbreak testing of staff and resident’s guidance, and the testing for professionals visiting care homes guidance.  During the lifetime of the extended Infection Control Fund, consistent feedback was received that certain providers had more significant IPC costs than others, due to the nature of the care provided (affecting staffing ratios) or due to the impact of a local outbreak. Accordingly, the size of the discretionary proportion to allow local authorities to provide additional support to such providers as necessary has increased.  Accordingly, local authorities have discretion over how to use the remaining 30% of the IPC allocation of the grant. The funding must nevertheless be used to support care providers to take additional steps to tackle the risk of COVID-19 infections, and the Department would like local authorities to consider using this fund to put in place IPC measures to support the resumption of services. Local authorities must use the remainder of the rapid testing allocation of the grant (nationally, 40% of the rapid testing funding) to support the care sector to operationally deliver lateral flow testing.  Guidance includes requirements for local authorities and requirements for providers. Annex provides a non-exhaustive list of examples of ways in which providers can spend funding as part of the ‘per beds’ or ‘per user’ allocation. |
|  | **Intended outcomes:**  … reduce the rate of COVID-19 transmission within and between care settings through effective IPC practices and increase uptake of staff vaccination.  …support additional lateral flow testing of staff in care homes, to enable indoors, close contact visiting where possible. |

| Testing for professionals visiting care homes (24)  Published: 20/05/2021  Author:  Department of Health and Social Care | **Aims:**  Guidance sets out the testing policy for health, social care and other professionals who may need to visit residents within care homes to provide services. |
| --- | --- |
|  | **Recommendations:**  **NHS professionals visiting care homes who are part of regular staff testing**   - Given the risks, the default position is that a visiting professional should not be allowed entry to a care home without proof of a negative test within the last 72 hours. - Care homes must ask the NHS professional when they were last tested and see proof of the result and date of the relevant professional’s test. - If the individual has not been tested (or is unable to provide proof) and it is not possible to test prior to entry, the care home will need to make a risk-based decision regarding whether to permit entry, considering the reason and urgency of the visit. The default position is that without proof of a recent negative test or a negative rapid LFT on the door of the care home, the professional should not be admitted.   **Testing for CQC inspectors visiting care homes**   - The CQC inspector should provide evidence to the care home or care setting of the negative rapid LFT result from earlier in the day when they arrive. As CQC inspectors by law have a right to enter a care setting as part of an inspection, they should not be denied access if they do not provide this evidence.   **Professionals not regularly tested through NHS or CQC staff testing**   - Professionals who are not part of a regular testing regime (that is, those who are not included in NHS staff and CQC inspectors) should be tested on the door of the care home. Professionals who have to visit multiple care homes per day do not need to be tested at a care home more than once a day. - Additional guidance provided for professionals who have recently tested positive for COVID-19 using a PCR test and void or invalid results. |
|  | **Implementation:**  Care home managers have ultimate responsibility for the safety of their care home, including the residents and staff. Guidance should be implemented by care homes and professionals by 22 March 2021 if not before.  Where the manager makes a risk-based decision to allow entry of someone without evidence of a negative test, all IPC measures must continue to be followed in order to mitigate the risk, including correct use of PPE, cleaning, ventilation and distancing. |
|  | **Intended outcomes:**  … it is essential that professionals and all staff are tested regularly before visiting care homes to reduce the risk of transmission across different settings and to help keep residents and staff safe.  … outlines how health, social care and other professionals visiting care homes can provide care homes with the necessary assurances.  …testing plays an important role by enabling the identification of a greater number of infectious individuals without symptoms who could be unknowingly passing the virus onto others, therefore helping keep care homes safe and safeguarding residents. |

| Adult social care extension to Infection Control and Testing Fund 2021 (25)  Published:  02/07/2021  Author:  Department of Health and Social Care | **Aims:**  The purpose of this fund is to support adult social care providers, including those with whom the local authority does not have a contract, to:   1. reduce the rate of COVID-19 transmission within and between care settings through effective IPC practices and increase uptake of staff vaccination; and 2. conduct testing of staff and visitors in care homes, high risk supported living and extra care settings, in order to enable close contact visiting where possible. |
| --- | --- |
|  | **Recommendations:**  This funding consists of two distinct allocations– IPC funding and testing funding. All direct funding must be used for the IPC measures or testing measures outlined.  **IPC funding**  Local authorities should pass 70% of this funding to care homes, including residential drug and alcohol services, and CQC-regulated community care providers within the local authority’s geographical area. Providers can use this funding to pay for the continuation of IPC measures they may have already taken if they are in line with these measures:  Care homes:   - ensuring that staff who are isolating in line with government guidance receive their normal wages and do not lose income while doing so. At the time of issuing the grant circular, this includes: - staff with suspected symptoms of COVID-19 waiting for a test, where a member of the staff’s household has suspected symptoms of COVID-19 and are waiting for a test, - where a member of the staff’s household has tested positive for COVID-19 and is therefore self-isolating, - any staff member for a period of at least 10 days following a positive test, - if a member of staff is required to quarantine prior to receiving certain NHS procedures (generally people do not need to self-isolate prior to a procedure or surgery unless their consultant or care team specifically asks them to). - limiting all staff movement between settings unless absolutely necessary, to help reduce the spread of infection. This includes staff who work for one provider across several care homes, staff that work on a part-time basis for multiple employers in multiple care homes or other care settings (for example in primary or community care). This includes agency staff. Mitigations such as block booking should be used to further minimise staff movement where agency or other temporary staff are needed. - limiting or cohorting staff to individual groups of residents or floors/wings, including segregation of COVID-19 positive residents. - to support active recruitment of additional staff (and volunteers) if they’re needed to enable staff to work in only one care home or to work only with an assigned group of residents or only in specified areas of a care home, including by using and paying for. - staff who have chosen to temporarily return to practice, including those returning through the NHS returners programme. These staff can provide vital additional support to homes and underpin effective IPC while permanent staff are isolating or recovering from COVID-19. - Costs of vaccination; including ensuring that staff who need to attend work or another location for the purposes of being vaccinated for COVID-19 are paid their usual wages to do so, any costs associated with reaching a vaccination facility, and any reasonable administrative costs associated with organising COVID-19 vaccinations where these were not being supported by other government funding streams.   **Testing funding**  Care homes can use this funding to pay for the continuation of measures that they may have already taken if they are in line with the below:     - Paying for staff costs associated with training and carrying out lateral flow testing, including time to: attend webinars, read online guidance and complete an online competency assessment:   - explain the full lateral flow test (LFT) process to those being tested, and ensuring that they understand all other IPC measures,   - ensure that any LFTs are completed properly, including overseeing the self-swabbing process, processing tests and logging results,   - wait for results, if staff are taking tests prior to their shift.   - Supporting safe visiting, including:   - welcoming visitors,   - gaining consent to conduct lateral flow testing,   - overseeing that PPE is correctly donned,   - additional IPC cleaning in between visits,   - alterations to allow safe visiting such as altering a dedicated space. - Costs associated with recruiting staff to facilitate increased testing. - Costs associated with the maintenance of a separate testing area where staff and visitors can be tested and wait for their result. This includes the cost of reduced occupancy where this is required to convert a bedroom into a testing area, but only if this is the only option available to the setting. We expect that most costs will have been covered by the first Rapid Testing Fund, which ran from December 2020 to March 2021. - Costs associated with disposal of LFTs and testing equipment. - Costs of PCR testing, including:   - ensuring that staff who need to attend work or another location for the purposes of being tested for COVID-19 are paid their usual wages to do so,   - any costs associated with reaching a testing facility,   - any reasonable administrative costs associated with organising and recording outcomes of COVID-19 tests.   **Local authority discretionary funding**  **IPC measures**  Local authorities must use 30% of the IPC allocation to support the care sector to put in place other COVID-19 infection control measures, but this can be allocated at their discretion. A non-exhaustive list of wider measures that the funding could be used for is below:   - providing additional support to care homes or other providers that are currently experiencing an outbreak to ensure that they are able to put in place sufficient IPC measures, - providing support on the IPC measures outlined above to a broader range of care settings, including, but not limited to: - community and day support services, - carers support services, - individuals who directly employ one or more personal assistants to meet their care needs, - individuals who are in receipt of direct payments, - the voluntary sector, - measures the local authority could put in place to boost the resilience and supply of the adult social care workforce in their area to support effective IPC, - steps to limit the use of public transport by members of staff (considering current government guidance on the safe use of other types of transport by members of staff), - providing accommodation for staff who proactively choose to stay separate from their families in order to limit social interaction outside work.   **Testing measures**  Local authorities to use their discretionary portion of the testing allocation to support:   - supported living and extra care settings eligible for LFTs, - care homes or other providers that are currently experiencing an outbreak to ensure that they have the resources needed to administer the LFTs and equipment that they need to increase lateral flow testing, - smaller homes to implement lateral flow testing as they may face relatively higher costs compared to large homes, - other parts of the sector with lateral flow testing in line with any further rollouts.   **Specific restrictions on the use of the funding**  **Staff who are off sick with conditions other than COVID-19**  This funding cannot be used by providers to pay usual wages to staff who are off sick with conditions other than COVID-19. This funding can be used to pay usual wages of staff who are self-isolating with suspected COVID-19 symptoms (rather than only after a positive test), but those individuals must be seeking to confirm whether this is COVID-19 through a test.  **PPE**  Local authorities may use their 30% discretionary portion of the IPC allocation on other COVID-19 IPC measures to support the care sector. This could include, for example, additional financial support for the purchase of PPE by providers or by the local authority directly (although not for costs already incurred), however the PPE portal is expected to be the first port of call for CQC-registered providers, and LRFs/local authorities for non-CQC registered providers.  **Designated settings**  The Infection Control and Testing Fund should only be used for the IPC and testing measures outlined. Any additional costs incurred by a designated setting to reach the standards to provide safe care for COVID-19 positive residents should be met from the hospital discharge programme.  **Visiting**  Where funding is spent on supporting visiting, this must be limited to measures that relate to managing the risks of COVID-19 transmission through visiting – in line with government guidance. Funding must not be spent on generic visiting facilities. |
|  | **Implementation:**  The funding will be paid to local authorities in July 2021. Expectation of the grant to be fully spent on IPC and testing measures by 30 September 2021. Care providers in receipt of LFTs are required to register the results of all tests as per the testing guidance for staff and residents, and the visitors and visiting professional’s guidance.  Provides links to the use of public transport, Test and Trace Support Payment Scheme, the Coronavirus Statutory Sick Pay Rebate Scheme, guidance on visiting care homes and guidance on designated settings. Guidance includes requirements for local authorities and requirements for providers. Annex provides a non-exhaustive list of examples of ways in which providers can spend funding as part of the ‘per beds’ or ‘per user’ allocation. |
|  | **Intended outcomes:**  … supporting care providers to reduce transmission and re-enabling close contact visiting.  …reduce the rate of COVID-19 transmission within and between care settings through effective IPC practices and increase uptake of staff vaccination.  …conduct testing of staff and visitors in care homes, high risk supported living and extra care settings, in order to enable close contact visiting where possible. |

| Coronavirus (COVID-19) vaccination of people working or deployed in care homes: operational guidance (26)  Published: 04/08/2021  Author:  [Department of Health and Social Care](https://web.archive.org/web/20200404122850/https:/www.gov.uk/government/organisations/department-of-health-and-social-care) | **Aims:**  These regulations require registered persons of all CQC registered care homes to ensure that a person does not enter the indoor premises unless they have been vaccinated. |
| --- | --- |
|  | **Recommendations:**  **Summary of the regulations**  The regulations require that all CQC-registered service providers (or registered managers) of accommodation for those who require nursing or personal care in a care home to ensure that a person does not enter the care home unless:   - the person resides in the care home used by the registered person (a resident), - the person has provided the registered person, (or those acting on behalf of the registered person) with satisfactory evidence that: - they have been vaccinated with the complete course of an authorised vaccine (the individual can prove they are fully vaccinated), - they, for clinical reasons, should not be vaccinated (the individual is exempt for medical reasons), - it is reasonably necessary for the person to provide emergency assistance in the care home. This includes a person is a friend or relative of the resident visiting the resident, a person visiting a resident who is dying, a person providing comfort or support to a resident in relation to a resident’s bereavement following the death of a relative or friend, or a person is under the age of 18.   **Residents**   - If someone is a resident or being admitted as a resident, they and an accompanying friend or relative will not require proof of vaccination. - The regulations require prospective residents and their families visiting care homes to provide evidence of vaccination or medical exemption. Care home managers may want to consider remote visits via video link for any prospective residents who are not vaccinated.   Further guidance provided on emergency assistance, services and urgent maintenance work.  **Friends, relatives and essential care givers**   - Friends, family (who also may be unpaid carers) and essential care givers will not need to show proof of vaccination or medical exemption. - Visits from family and friends are vital for the health and wellbeing of people living in care homes. It would be unjustifiably detrimental to residents to deprive them of contact with, and care from, their loved ones.   **Death and bereavement**  People do not need to show proof of vaccination or exemption if they are visiting a resident who is dying (that is in their last days of life) or they are providing comfort or support to a resident following the death of a relative or friend.  **Recruitment of new staff**  The regulations also apply to new staff recruited by the registered provider. It is important to note that only new recruits that have had a full course of a Medicines and Healthcare products Regulatory Agency approved COVID-19 vaccine or are medically exempt from the requirement are eligible to work in the care home. |
|  | **Implementation:**  From 11th November 2021, all care home workers, and anyone entering a care home, will need to be fully vaccinated, unless they are exempt under the regulations. Grace period for vaccination starts 22 July 2021, the last date for care home workers to get their first dose so they are fully vaccinated by the time the regulations come into force 16 September 2021 and the regulations come into force 11 November 2021.  Guidance links to the National Booking Service, other pathways to vaccination and a range of published resources with information about the vaccine that can be used to support difficult conversations, including the Vaccine Communications Toolkit for Adult Social Care.  **Summary of the regulations**  The vaccination requirement only applies to people who go inside a care setting. As long as someone is not entering the building, they would not need to show vaccination status.  **Registered persons**  The registered person is responsible for ensuring that everyone who enters their care home is either vaccinated or exempt. The registered person will be the person registered with the CQC as a manager or service provider. Provides general guidance on demonstrating evidence, guidance on medical exemptions, and guidance for registered persons.  **Guidance for staff**  For care home staff, this means that they will only be able to continue to work inside a care home if they are vaccinated, unless they are  a) under the age of 18, b) medically exempt.  **Redeployment** – care home manager should explore all options available to unvaccinated staff. This could include moving staff to an alternative role; however, it should not be assumed that it will be possible for staff to be redeployed.  **Dismissal** - If staff are unable to provide proof of vaccination or exemption, then all options available should be explored, however the regulations may provide a fair reason for dismissal.  Guidance for residents and relatives and friends of residents provided, alongside guidance for visiting professionals. Includes links to wider support and guidance for local authorities. |
|  | **Intended outcomes:**  … policy has been introduced to ensure the safety of you, other residents and staff to reduce the risk of COVID-19 spreading in the home. |

| Adult Social Care Infection Control and Testing Fund: round 3 (27)  Published:  21/10/2021  Author:  Department of Health and Social Care | **Aims:**  The purpose of this fund is to support adult social care providers (including those with whom the local authority does not have a contract) to:   - reduce the rate of COVID-19 transmission within and between care settings through effective IPC practices and increase COVID-19 and flu vaccine uptake among staff. - conduct testing of staff and visitors in care settings to identify and isolate positive cases, and in order to enable close contact visiting where possible. |
| --- | --- |
|  | **Recommendations:**  The 'direct funding for providers' portion of this funding consists of three distinct allocations:   - IPC funding, - vaccines funding, - testing funding.   **IPC funding**  Local authorities should pass the direct funding to care homes, including residential drug and alcohol services, and CQC-regulated community care providers within the local authority’s geographical area. This represents 70% of the total IPC allocation. Providers can use this funding to pay for the continuation of IPC measures they may have already taken if they are in line with these measures.  **For care homes (including residential drug and alcohol settings):**   - ensuring that staff who are isolating in line with government guidance receive their normal wages and do not lose income while doing so. It is important to note that self-isolation guidance for fully vaccinated and unvaccinated or partially vaccinated (not exempt) staff differs, as reflected below. At the time of issuing the grant circular, this includes: - all staff with suspected symptoms of COVID-19 waiting for a test, - all staff members with a positive lateral flow test (LFT) waiting for a PCR test result, - all staff members for a period of 10 days following a positive PCR test, - any staff member unvaccinated or partially vaccinated (not exempt) identified as a contact of a COVID-19 case while isolating as advised by Test and Trace or organisation, - any staff member fully vaccinated that develops symptoms after being identified as a contact of a COVID-19 case and is therefore required to isolate while waiting for a test result (if positive the above procedure applies), - any staff member who is not required to self-isolate but is asked not to work, in line with government guidance. - limiting staff movement between settings in line with the latest guidance, to help reduce the spread of infection. This includes staff who work for one provider across several care homes, staff that work on a part-time basis for multiple employers in multiple care homes or other care settings (for example in primary or community care). This includes agency staff. - limiting or cohorting staff to individual groups of residents or floors/wings, including segregation of COVID-19 positive residents. - to support active recruitment of additional staff (and volunteers) if they're needed to enable staff to work in only one care home or to work only with an assigned group of residents or only in specified areas of a care home, including by using and paying for staff who have chosen to temporarily return to practice, including those returning through the NHS returners programme. These staff can provide vital additional support to homes and underpin effective IPC while permanent staff are isolating or recovering from COVID-19.   **Vaccines funding**  Each local authority has received an allocation of funding that has been specifically made available to support care providers and social care staff with the costs associated with accessing COVID-19 and flu vaccinations. This funding has been made available to support the following measures:   - ensuring that staff who need to attend work or another location for the purposes of being vaccinated for COVID-19 or flu are paid their usual wages to do so, - any costs associated with reaching a vaccination facility, - any reasonable administrative costs associated with organising COVID-19 or flu vaccinations where these were not being supported by other government funding streams.   **Testing funding**  At a national level, this represents 70% of the testing allocation. Care homes can use this funding to pay for the continuation of measures that they may have already taken if they are in line with the below:   - paying for staff costs associated with training, including time to: attend webinars, read online guidance, for example guidance on new test kit types, and complete an online competency assessment, - costs associated with conducting visitor tests to support safe visiting in care settings, including: - welcoming visitors, - gaining consent to conduct lateral flow testing, - overseeing that PPE is correctly donned, - additional IPC cleaning in between visits, - ensuring that any LFTs are completed properly, including overseeing the self-swabbing process, processing tests and logging results, - costs associated with staff lateral flow testing, for example, for staff to wait for results if staff are taking tests onsite prior to their shift, - costs associated with recruiting staff to facilitate increased testing, - costs associated with the maintenance of a separate testing area where staff and visitors can be tested and wait for their result, - costs associated with disposal of LFTs and testing equipment, - costs of PCR testing, including: - ensuring that staff who need to attend work or another location for the purposes of being tested for COVID-19 are paid their usual wages to do so, - any costs associated with reaching a testing facility, - any reasonable administrative costs associated with organising and recording outcomes of COVID-19 tests.   **Local authority discretionary funding**  The local authority discretionary portion of this funding consists of 3 allocations:   - IPC funding, - vaccines funding, - testing funding.   **IPC measures**  Local authorities must use 30% of the IPC allocation to support the care sector to put in place other COVID-19 infection control measures, but this can be allocated at their discretion. A non-exhaustive list of wider measures that the funding could be used for is:   - providing additional support to care homes or other providers that are currently experiencing an outbreak to ensure that they are able to put in place sufficient IPC measures, - providing support on the IPC measures outlined above to a broader range of care settings, including, but not limited to: - community and day support services, - carers support services, - individuals who directly employ one or more personal assistants to meet their care needs, - individuals who are in receipt of direct payments, - the voluntary sector, - having measures the local authority could put in place to boost the resilience and supply of the adult social care workforce in their area to support effective IPC - taking steps to limit the use of public transport by members of staff (considering current government guidance on the safe use of other types of transport by members of staff), - providing accommodation for staff who proactively choose to stay separate from their families in order to limit social interaction outside work.   **Vaccines measures**  Local authorities should use 30% of their vaccines allocation to support staff in other care settings, including non-registered settings, and to provide additional support to providers where required in order to undertake the following measures:   - ensuring that staff who need to attend work or another location for the purposes of being vaccinated for COVID-19 or flu are paid their usual wages to do so, - any costs associated with reaching a vaccination facility, - any reasonable administrative costs associated with organising COVID-19 or flu vaccinations where these were not being supported by other government funding streams.   **Testing measures**  As such, we expect local authorities to use their discretionary portion of the testing allocation to support:   - supported living and extra care settings eligible for LFTs (for eligibility see guidance on testing service for extra care and supported living settings, - care homes or other providers that are currently experiencing an outbreak to ensure that they have the resources needed to administer the LFTs and equipment that they need to increase lateral flow testing, - smaller homes who may face relatively higher costs compared to large homes and as such who may require additional support, - CQC-regulated community care providers with the costs of PCR testing; including ensuring that staff who need to attend work or another location for the purposes of being tested for COVID-19 are paid their usual wages to do so, any costs associated with reaching a testing facility, and any reasonable administrative costs associated with organising and recording outcomes of COVID-19 tests. - other parts of the sector using lateral flow tests as part of an adult social care testing regime that can demonstrate need for funding.   **Specific restrictions on the use of the funding**  **Staff who are off sick with conditions other than COVID-19**  This funding cannot be used by providers to pay usual wages to staff who are off sick with conditions other than COVID-19. This funding can be used to pay usual wages of staff who are self-isolating with suspected COVID-19 symptoms (rather than only after a positive test), but those individuals must be seeking to confirm whether this is COVID-19 through a test. In these circumstances, where a member of staff receives a negative test for COVID, a provider can still use this fund to pay usual wages where the symptoms were suspected to be COVID-19, in line with government guidance.  **PPE**  Local authorities may use their 30% discretionary portion of the IPC allocation on other COVID-19 IPC measures to support the care sector. This could include, for example, additional financial support for the purchase of PPE by providers or by the local authority (although not for costs already incurred), the PPE portal is expected to be the first port of call for CQC-registered providers, and LRFs/local authorities for non-CQC registered providers.  **Designated settings**  Therefore, the Infection Control and Testing Fund should only be used for the IPC and testing measures outlined. Any additional costs incurred by a designated setting to reach the standards to provide safe care for COVID-19 positive residents should be met from the hospital discharge programme.  **Visiting**  Where funding is spent on supporting visiting, this must be limited to measures that relate to managing the risks of COVID-19 transmission through visiting – in line with government guidance. Funding must not be spent on generic visiting facilities. |
|  | **Implementation:**  The funding will be paid in 2 tranches. The first 60% of the fund will be paid to local authorities in October 2021. The remaining 40% of the fund will be paid in January 2022. This will include allocations for IPC, vaccines and testing.  Provides links to the use of public transport, Test and Trace Support Payment Scheme, the Coronavirus Statutory Sick Pay Rebate Scheme, guidance on visiting care homes and guidance on designated settings.  Guidance includes requirements for local authorities and requirements for providers. Annex provides a non-exhaustive list of examples of ways in which providers can spend funding as part of the ‘per beds’ or ‘per user’ allocation. |
|  | **Intended outcomes:**  …reduce the rate of COVID-19 transmission within and between care settings through effective IPC practices and increase COVID-19 and flu vaccine uptake among staff.  …conduct testing of staff and visitors in care settings to identify and isolate positive cases, and in order to enable close contact visiting where possible. |

| Workforce Recruitment and Retention Fund for adult social care (28)  Published: 03/11/2021  Author:  Department of Health and Social Care | **Aims:**  The purpose of this funding is to support local authorities to address adult social care workforce capacity pressures in their geographical area through recruitment and retention activity this winter. |
| --- | --- |
|  | **Recommendations:**  The purpose of this funding is to support local authorities to address adult social care workforce capacity pressures in their geographical area through recruitment and retention activity this winter, in order to:   - support providers to maintain the provision of safe care and bolstering capacity within providers to deliver more hours of care, - support timely and safe discharge from hospital to where ongoing care and support is needed, - support providers to prevent admission to hospital, - enable timely new care provision in the community, - support and boost retention of staff within social care.   This allocation of the grant must only be used to deliver measures that address local workforce capacity pressures in adult social care. Guidance provides examples of:   - supporting payments to boost the hours provided by the existing workforce – including childcare costs and overtime payments, - investment in measures to support staff and boost retention of staff within social care – including occupational health, wellbeing measures, incentive and retention payments, - the creation and maintenance of measures to secure additional or redeployed capacity from current care workers. For example, shared staff banks, redeploying local authority staff, emergency support measures, overtime payments, - local recruitment initiatives, - activities to support hospital discharge or to prevent or address delays as a result of workforce capacity shortages, - activities which support the recruitment of local authority employed social care staff, or which enhance or retain the capacity of existing local authority employed social care staff, - local authorities and, where funding has been passported, providers to use the grant to cover reasonable administrative and/or set up costs they incur for new measures that deliver additional staffing capacity through recruitment and retention activity. |
|  | **Implementation:**  This is a ring-fenced grant of £162.5 million that will be paid in two instalments to local authorities, the first instalment worth £97.5 million (60%) will be paid in November 2021, the second instalment worth £65 million (40%) will be paid in January 2022.  The department's expectation is that the grant will be fully spent on addressing local workforce capacity pressures through recruitment and retention activity by 31 March 2022.  The funding must be used to deliver new or additional measures which support the purpose of the fund, or the funding can be used to increase the scale of activities which already deliver additional workforce capacity where these exist within the local authority or providers.  Guidance includes reporting requirements and financial management. |
|  | **Intended outcomes:**  The main purpose of the Workforce Recruitment and Retention Fund is to support local authorities to address adult social care workforce capacity pressures in their geographical area this winter, in order to:   - support providers to maintain the provision of safe care and bolstering capacity within providers to deliver more hours of care, - support timely and safe discharge from hospital to where ongoing care and support is needed, - support providers to prevent admission to hospital, - enable timely new care provision in the community, - support and boost retention of staff within social care. |

| Workforce Recruitment and Retention Fund for adult social care, round 2 (29)  Published: 16/12/2021  Author:  Department of Health and Social Care | **Aims:**  The main purpose of the Workforce Recruitment and Retention Fund, round 2, is to support local authorities to urgently address adult social care workforce capacity pressures in their geographical area through recruitment and retention activity this winter. |
| --- | --- |
|  | **Recommendations:**  The main purpose of the Workforce Recruitment and Retention Fund, round 2, is to support local authorities to urgently address adult social care workforce capacity pressures in their geographical area through recruitment and retention activity this winter, in order to:   - support timely and safe discharge from hospital to where ongoing care and support is needed, - support providers to maintain the provision of safe care and bolstering capacity within providers to deliver more hours of care, - support providers to prevent admission to hospital, - enable timely new care provision in the community, - support and boost retention of staff within social care.   This allocation of the grant must only be used to deliver measures that address local workforce capacity pressures in adult social care between 10 December 2021 and 31 March 2022 through recruitment and retention activity. It will be important to retain existing staff capacity as well as encourage new and returning entrants. Examples of this include, but are not limited to:   - activities to support hospital discharge or to prevent or address delays because of workforce capacity shortages, - enhancing and bolstering measures already undertaken through the Workforce Recruitment and Retention Fund (round 1), - supporting payments to boost the hours provided by the existing workforce – including childcare costs and overtime payments, - the creation and maintenance of measures to secure additional or redeployed capacity from current care workers. For example, staff banks and redeploying local authority staff in line with national staff movement guidance, emergency support measures and overtime payments, - local recruitment initiatives, - activities which support the recruitment of local authority employed social care staff, or which enhance or retain the capacity of existing local authority employed social care staff, - local authorities and, where funding has been passported, providers may also use the grant to cover reasonable administrative and set up costs they incur for new measures that deliver additional staffing capacity through recruitment and retention activity.   Noting the link between adult social care workforce capacity and hospital discharge, local authorities should engage with CCGs and NHS trusts to work collaboratively to address workforce pressures which in some areas are delaying people from returning to their home from hospital. |
|  | **Implementation:**  This is a ring-fenced grant of £300 million that will be paid in two instalments to local authorities, the first instalment worth £210 million (70%) will be paid in early in January 2022, the second instalment worth £90 million (30%) will be paid in February 2022.  The department's expectation is that the grant will be fully spent on addressing local workforce capacity pressures through recruitment and retention activity by 31 March 2022.  Unlike round 1 of the Workforce Recruitment and Retention Fund, the second round of the fund can be used for bringing forward planned uplifts relating to pay in advance of the new financial year.  This grant is separate from the extension to the Infection Control and Testing Fund, and as such cannot be used to support those infection control, vaccine or testing measures. This funding cannot be used to address general financial pressures that providers might be experiencing. All providers should be completing the Capacity Tracker regularly. In order to receive passported funding through this grant, providers must already be completing the Capacity Tracker at least once per week.  Guidance includes reporting requirements and financial management. |
|  | **Intended outcomes:**  The main purpose of the Workforce Recruitment and Retention Fund, round 2, is to support local authorities to address adult social care workforce capacity pressures in their geographical area this winter, in order to:   - support timely and safe discharge from hospital to where ongoing care and support is needed, - support providers to maintain the provision of safe care and bolstering capacity within providers to deliver more hours of care, - support providers to prevent admission to hospital, - enable timely new care provision in the community, - support and boost retention of staff within social care. |

| Adult Social Care Omicron Support Fund: guidance (30)  Published: 10/01/2022  Author:  Department of Health and Social Care | **Aims:**  The purpose of this fund is to support the sector with measures already covered by the infection prevention and control (IPC) allocation of the Infection Control and Testing Fund (round 3) to reduce the rate of COVID-19 transmission within and between care settings through effective IPC practices. |
| --- | --- |
|  | **Recommendations:**  The purpose of this fund is to support the sector with measures already covered by the infection prevention and control (IPC) allocation of the Infection Control and Testing Fund (round 3) to reduce the rate of COVID-19 transmission within and between care settings through effective IPC practices.  Additionally, this funding may also be used to increase ventilation in care homes, and to enhance local authorities’ current direct payment offer particularly when the only way a person’s care needs can be met is by a friend or family member, or to enhance support for carers. It may also be used to pay for temporary staffing to cover increased staff absence caused by COVID-19 and maintain staffing levels and workforce capacity.  Local authorities have discretion to use the funding as needed locally, to support the adult social care sector, including relevant local authority staff, in its COVID-19 response, and in particular increased challenges posed by the Omicron variant. Local authorities should consider the following as appropriate uses of the grant:   - ensuring that staff who are isolating in line with government guidance receive their normal wages and do not lose income while doing so, - paying for temporary cover for staff who are unable to work because they are unwell due to COVID-19and/or are isolating in line with government guidance, to maintain safe workforce capacity in care services, - paying for temporary staffing (overtime or agency) to maintain safe staffing levels, - limiting staff movement between settings in line with the latest guidance, to help reduce the spread of infection – this includes staff who work for one provider across several settings, staff that work on apart-time basis for multiple employers and agency staff, - limiting or cohorting staff to individual groups of people receiving care, including segregation of COVID-19 positive residents in care homes - steps to limit the use of public transport by members of staff, - providing accommodation for staff who proactively choose to stay separate from their families in order to limit social interaction outside work, - support to providers in purchasing CO2 monitors or air cleaners for use in care homes to monitor and improve ventilation, - enhancing local authorities’ current direct payment offer to support care provided by friends and family, including any additional support the carer may need to assist them to continue in their caring role, - providing additional support to care homes or other providers that are currently experiencing an outbreak to ensure that they are able to put in place sufficient IPC measures, - providing support to community groups and paying volunteer expenses. |
|  | **Implementation:**  This is a grant of £60 million that will be paid in full in January 2022. The reporting requirements for this grant have been minimised in order to prioritise speed.  Local authorities are not required to pass funding on to providers. However, where local authorities choose to pass funding on, they must only pay funding to providers who have completed the Capacity Tracker at least twice (2 consecutive weeks) and have committed to completing the Tracker at least once per week until the conclusion of the fund.  Guidance includes reporting requirements and financial management. |
|  | **Intended outcomes:**  … reduce the rate of COVID-19 transmission within and between care settings through effective IPC practices. |

| A guide to the spring booster for those aged 75 years and older residents in care homes (31)  Published: 03/03/2022  Author:  UK Health Security Agency | **Aims:**  … people aged 75 years and over, those in care homes and those aged 12 years and over with a weakened immune system are being offered the spring booster, as protection from the vaccine may be lower and may decline more quickly older people and those with a weakened immune system. |
| --- | --- |
|  | **Recommendations:**  People aged 75 years and older, residents in care homes for older people, and those with weakened immune systems will be offered a spring booster of COVID-19 vaccine. Appointments will be available from the National Booking Service shortly. |
|  | **Implementation:**  This group should be offered an appointment around 6 months (and not before 3 months) since their last dose of vaccine. Discusses common side effects and includes links to further information. |
|  | **Intended outcomes:**  … this booster should help to reduce your risk of being admitted to hospital with COVID-19. |

| Infection prevention and control in adult social care: COVID-19 supplement (32)  Published: 31/03/2022  Author:  [Department of Health and Social Care](https://web.archive.org/web/20200404122850/https:/www.gov.uk/government/organisations/department-of-health-and-social-care) | **Aims:**  Guidance should be used to help reduce the spread of COVID-19 in adult social care settings. Supplement provides additional information regarding safe working when caring for people with COVID-19 in the provision of adult social care services. |
| --- | --- |
|  | **Recommendations:**  The supplement includes guidance on:  **Staff IPC considerations**  **Vaccination**  All people working in health and social care settings, including volunteers and unpaid carers, have a responsibility to be vaccinated against COVID-19.  **Testing**  Signposting to regular testing in adult social care.  **PPE**  Appropriate PPE should be worn by care workers and visitors to residential care settings, subject to a risk assessment. Guidance provided on gloves, aprons and eye protection, face masks and aerosol-generating procedures.  **Staff movement**   - Care homes are not normally required to limit staff movement between sites or services; however, they may be asked to limit staff movement by the local Director of Public Health or HPT if there is high prevalence of COVID-19 locally or in an outbreak. - Guidance if a staff member develops COVID-19 symptoms, if a staff member receives a positive lateral flow or PCR test result – advice regarding self-isolation and links to further guidance. In addition, social care staff with COVID-19 should not attend work until they have had 2 consecutive negative lateral flow test results (taken at least 24 hours apart), they feel well, and they do not have a high temperature. Staff who are contacts of confirmed cases can continue working.   **IPC considerations for people receiving care:**  **Vaccination**  Wherever possible they are encouraged to get their COVID-19 vaccines ahead of entering adult social care settings.  **Testing**  Signposts to the COVID-19 vaccination: guide for adults and adult social care testing guidance.  **Environmental considerations**  Guidance provided on ventilation and waste management.  **Considerations specific to care homes**   - **Admission of care home residents from a care facility or the community** Residents should take both of the following: a PCR test within the 72 hours before they’re admitted (or a lateral flow test if they have tested positive for COVID-19 in the past 90 days) and a lateral flow test on the day of admission. These tests should be provided by the care home. If an individual tests positive on either of these tests and continues to be admitted to the care home, they should be isolated on arrival and follow the guidance on care home residents who are symptomatic or test positive for COVID-19. - **Discharge from hospital into a care home** The NHS will do a PCR test within 48 hours prior to an individual’s discharge into a care home, or a lateral flow test if the individual has tested positive for COVID-19 in the last 90 days. The test result should be shared with the individual themselves, their key relatives or advocate and the relevant care provider before the discharge takes place. If an individual tests positive prior to discharge, they can be admitted to the care home, if the home is satisfied, they can be cared for safely. They should be isolated on arrival for 10 days and follow the guidance below on care home residents who are symptomatic or test positive for COVID-19. - Care home residents who are close contacts of a COVID-19 case are no longer advised to isolate nor undertake additional testing. - Care home residents who are symptomatic or test positive for COVID-19 - All residents who test positive for COVID-19 with either lateral flow or PCR tests, regardless of whether they are symptomatic or asymptomatic, should isolate in the care home for 10 days from when the symptoms started, or from the date of the test if they did not have symptoms. Isolation does not preclude receiving one visitor, going into outdoor spaces within the care home grounds through a route where they are not in contact with other care home residents. - **Support caring for care home residents who test positive for COVID-19** Consideration should be given to having a smaller number of workers dedicated to supporting the person during their infectious period.   **Visiting arrangements in care homes**   - There should not normally be any restrictions to visits into or out of the care home. The right to private and family life is a human right protected in law. Where visiting is modified during an outbreak of COVID-19 or where a care home resident has confirmed COVID-19, every resident should be enabled to continue to receive one visitor inside the care home. End-of-life visiting should always be supported, and testing is not required in any circumstances for an end-of-life visit. Visitors who are providing personal care should wear appropriate PPE and have a negative COVID-19 lateral flow test result from a LFD before entering a care home, unless medically exempt. - Visiting professionals - guidance for NHS staff, and CQC inspectors.   **Outbreak management**   - An outbreak consists of 2 or more positive (or clinically suspected) linked cases of COVID-19 associated with the same setting within a 14-day period. This applies to both staff and residents and includes PCR and lateral flow test results. |
|  | **Implementation:**  **Staff IPC considerations**  **Vaccination**   - To minimise risk to people who receive care and support, health and social care providers should encourage and support all their staff to get a COVID-19 vaccine and a booster dose as and when they are eligible, as well a vaccine for seasonal influenza. - Providers can do this by putting in place arrangements to facilitate staff access to vaccinations, and regularly reviewing the immunisation status of their workforce. - To ensure the safety of people who receive care, providers should undertake risk assessments wherever possible. These should consider the COVID-19 vaccination status of both staff members and the people they care for. - Signposts to national booking service for vaccination.   **Testing**  Signposting to regular testing in adult social care.  **PPE**  Signposts to instructions for donning and doffing PPE.  **Staff movement**  Signposting to regular testing in adult social care and guidance on staying at home/isolation.  **Vaccination**  Signposts to the COVID-19 vaccination: guide for adults and adult social care testing guidance.  **Environmental considerations**  Signposts to standard precautions and IPC: resource for adult social care.  **Considerations specific to care homes**  Signposts to guidance on who is at higher risk of becoming severely unwell, and the Care Provider Alliance has produced guidance on COVID-19 oximetry at home.  **Outbreak management**   - If an outbreak is suspected, the HPT should be informed. A risk assessment should be undertaken with the HPT or other local partner to see if the clinical situation can be considered an outbreak and if outbreak management measures are needed. - If an outbreak is declared as a result of the risk assessment, then measures will be taken. These will include testing and may also include temporarily stopping or reducing communal activities. closure of the home to further admissions restriction of movement of staff providing direct care to avoid ‘seeding’ of outbreaks between different settings. - Changes to visiting: some forms of visiting should continue if individual risk assessments are carried out. One visitor per resident should always be able to visit inside the care home. - In specific situations, where the local or national risk assessment indicates that cases may be caused by a variant with vaccine escape potential or other concerns, additional measures may be advised. |
|  | **Intended outcomes:**  ….to help reduce the spread of COVID-19 in adult social care settings. |

| Health and wellbeing of the adult social care workforce (33)  Published: 19/05/2022  Author:  [Department of Health and Social Care](https://web.archive.org/web/20200404122850/https:/www.gov.uk/government/organisations/department-of-health-and-social-care) | **Aims:**  Guidance includes tips, advice and toolkits for employers and managers to use, to help build the resilience of their team and address any concerns their staff may have.  Guidance also includes how social care workers can manage their personal mental health in the current circumstances. |
| --- | --- |
|  | **Recommendations:**  **Staff wellbeing and support**   - Signposts to listening service, talking therapies and coaching, and improving access to occupational health services. - Employers encouraged to create ‘Wellness Action Plans’. - Advice for managers on maintaining team resilience available on the Skills for Care website.   **Support for registered managers**   - Registered manager networks offering local management support, including Facebook and WhatsApp groups. - Advice telephone line and email inbox provided by Skills for Care. - Specific guidance provided for employees with underlying health conditions - if an employee cannot work from home, it is no longer advised that they do not attend the workplace. - Employers should have individual conversations with all members of their workforce who have underlying health conditions or are otherwise identified as being at increased risk, before a return to work or a return to a previous role if the employee can continue to work from home in their current role, they should be supported to do so. |
|  | **Implementation:**  **Staff wellbeing and support**   - Signposts to Adult Social Care Reform White Paper. - Signposting to wellbeing, support and resilience building resources. - Signposting to Digital Social Care for guidance on best practice for information sharing and how technology can be used to support staff and the people they care for. - Signposting to Skills for Care Workforce Wellbeing Resource Finder. - Further guidance provided in maintaining personal mental wellbeing, building resilience and managing stress and anxiety, physical wellbeing and financial wellbeing. - Example of good practice also provided. |
|  | **Intended outcomes:**  Not explicitly stated, however states that the government is committed to helping staff recover from their extraordinary role in helping the country through the pandemic. To ensure employers have the resources to build resilience and address staff concerns, related to COVID-19 and beyond. |

**Acronyms:** Acute respiratory distress syndrome (ARDS), Care Quality Commission (CQC), Clinical commissioning groups (CCGs), Continuing Healthcare (CHC), Data Security Protection Toolkit (DSPT), Department for Health and Social Care (DHSC), Deprivation of Liberty Safeguards (DoLS), Disclosure and Barring Service (DBS), Do not attempt cardio-pulmonary resuscitation (DNACPR), General Practitioner (GP), Health Protection Team (HPT), Infection Prevention and Control (IPC), Lateral flow device (LFD), Local Resilience Forums (LRF), Mental Capacity Act (MCA), Multidisciplinary team (MDT), National Health Service (NHS), National Supply Disruption Response (NSDR), North of England Commissioning Support (NECS), Office for National Statistics (ONS), Personal protective equipment (PPE), Primary Care Networks (PCNs), Polymerase chain reaction (PCR), Public Health England (PHE), Social Care Institute for Excellence (SCIE)

Where possible, text has been replicated verbatim from the document identified, or paraphrased to aid understanding. In cases where guidance signposts to previously published guidance, the data was not extracted a second time, but is referred to.

**References**

1. Public Health England. Guidance for social or community care and residential settings on COVID-19 2020 [updated 25 February 2020. Available from: <https://www.gov.uk/government/publications/guidance-for-social-or-community-care-and-residential-settings-on-covid-19/guidance-for-social-or-community-care-and-residential-settings-on-covid-19>

2. Department of Health and Social Care. Coronavirus (COVID-19): hospital discharge service requirements 2020 [updated 19 March 2020. Available from: [https://www.gov.uk/government/publications/coronavirus-covid-19-hospital-discharge-service-requirements](https://web.archive.org/web/20200322081118/https://www.gov.uk/government/publications/coronavirus-covid-19-hospital-discharge-service-requirements).

3. Department of Health and Social Care. Responding to COVID-19: the ethical framework for adult social care 2020 [updated 19 March 2020. Available from: [https://www.gov.uk/government/publications/covid-19-ethical-framework-for-adult-social-care/responding-to-covid-19-the-ethical-framework-for-adult-social-care.](https://web.archive.org/web/20200319100944/https://www.gov.uk/government/publications/covid-19-ethical-framework-for-adult-social-care/responding-to-covid-19-the-ethical-framework-for-adult-social-care.)

4. Department of Health and Social Care, Care Quality Commission, Public Health England, NHS England. Coronavirus (COVID-19): admission and care of people in care homes 2020 [updated 02 April 2020. Available from: [https:/www.gov.uk/government/publications/coronavirus-covid-19-admission-and-care-of-people-in-care-homes.](https://web.archive.org/web/20200404122850/https:/www.gov.uk/government/publications/coronavirus-covid-19-admission-and-care-of-people-in-care-homes.)

5. Public Health England. COVID-19: management of exposed health care workers and patients in hospital settings 2020 [updated 04 April 2020. Available from: https://www.gov.uk/government/publications/covid-19-management-of-exposed-healthcare-workers-and-patients-in-hospital-settings.

6. Department of Health and Social Care. Coronavirus (COVID-19): looking after people who lack mental capacity 2020 [updated 09 April 2020. Available from: <https://www.gov.uk/government/publications/coronavirus-covid-19-looking-after-people-who-lack-mental-capacity>.

7. Department of Health and Social Care. COVID-19: our action plan for adult social care 2020 [updated 14 December 2020. Available from: <https://www.gov.uk/government/publications/coronavirus-covid-19-adult-social-care-action-plan/covid-19-our-action-plan-for-adult-social-care>.

8. Public Health England. COVID-19: how to work safely in care homes 2020 [updated 17 April 2020. Available from: <https://www.gov.uk/government/publications/covid-19-how-to-work-safely-in-care-homes>.

9. Department of Health and Social Care. Coronavirus (COVID-19): care home support package 2020 [updated 15 May 2020. Available from: <https://www.gov.uk/government/publications/coronavirus-covid-19-support-for-care-homes.>

10. Department of Health and Social Care. Coronavirus (COVID-19): reducing risk in adult social care 2020 [updated 19 June 2020. Available from: <https://www.gov.uk/government/publications/coronavirus-covid-19-reducing-risk-in-adult-social-care>.

11. Department of Health and Social Care. Update on policies for visiting arrangements in care homes 2020 [updated 22 July 2020. Available from: <https://www.gov.uk/government/publications/visiting-care-homes-during-coronavirus>

12. UK Health Security Agency. PPE guide for community and social care settings 2020 [updated 31 July 2020. Available from: <https://www.gov.uk/government/publications/personal-protective-equipment-ppe-illustrated-guide-for-community-and-social-care-settings>.

13. Department of Health and Social Care. Overview of adult social care guidance on coronavirus (COVID-19) 2020 [updated 25 August 2020. Available from: <https://www.gov.uk/guidance/overview-of-adult-social-care-guidance-on-coronavirus-covid-19>.

14. Department of Health and Social Care. Adult social care: our COVID-19 winter plan 2020 to 2021 2020 [updated 18 September 2020. Available from: <https://www.gov.uk/government/publications/adult-social-care-coronavirus-covid-19-winter-plan-2020-to-2021/adult-social-care-our-covid-19-winter-plan-2020-to-2021>.

15. Department of Health and Social Care. About the Adult Social Care Infection Control Fund 2020 [updated 15 June 2021. Available from: <https://www.gov.uk/government/publications/adult-social-care-infection-control-fund/about-the-adult-social-care-infection-control-fund>.

16. Department of Health and Social Care. Adult Social Care Infection Control Fund – round 2: guidance 2020 [updated 01 July 2022. Available from: <https://www.gov.uk/government/publications/adult-social-care-infection-control-fund-round-2/adult-social-care-infection-control-fund-round-2-guidance>.

17. Care Quality Commission, Department of Health and Social Care, NHS England, Public Health England. Discharge into care homes: designated settings 2020 [updated 11 February 2022. Available from: <https://www.gov.uk/government/publications/designated-settings-for-people-discharged-to-a-care-home/discharge-into-care-homes-designated-settings>.

18. Department of Health and Social Care. Adult Social Care Rapid Testing Fund: guidance 2020 [updated 01 July 2022. Available from: <https://www.gov.uk/government/publications/adult-social-care-rapid-testing-fund/adult-social-care-rapid-testing-fund-guidance#background>.

19. Department of Health and Social Care. Your care home during winter 2021 [updated 01 April 2021. Available from: <https://www.gov.uk/government/publications/coronavirus-covid-19-support-for-care-homes/your-care-home-during-winter>.

20. Department of Health and Social Care. Workforce Capacity Fund for adult social care 2021 [updated 29 January 2021. Available from: <https://www.gov.uk/government/publications/workforce-capacity-fund-for-adult-social-care>.

21. Department of Health and Social Care. Restricting workforce movement between care homes and other care settings 2021 [updated 01 March 2021. Available from: <https://www.gov.uk/government/publications/restricting-workforce-movement-between-care-homes-and-other-care-settings>.

22. UK Health Security Agency. Coronavirus (COVID-19) testing for adult social care services 2021 [updated 24 March 2021. Available from: <https://www.gov.uk/government/publications/coronavirus-covid-19-testing-for-adult-social-care-settings>.

23. Department of Health and Social Care. Adult social care infection control and testing fund 2021 [updated 29 March 2021. Available from: <https://www.gov.uk/government/publications/adult-social-care-infection-control-and-testing-fund.>

24. Department of Health and Social Care. Testing for professionals visiting care homes 2021 [updated 01 April 2022. Available from: <https://www.gov.uk/government/publications/coronavirus-covid-19-testing-in-adult-care-homes/testing-for-professionals-visiting-care-homes>.

25. Department of Health and Social Care. Adult Social Care Extension to Infection Control and Testing Fund Ring-Fenced Grant 2021 2021 [updated 02 July 2021. Available from: <https://www.gov.uk/government/publications/adult-social-care-extension-to-infection-control-and-testing-fund-2021>.

26. Department of Health and Social Care. COVID-19 vaccination of people working or deployed in care homes: operational guidance 2021 [updated 04 August 2021. Available from: <https://www.gov.uk/government/publications/vaccination-of-people-working-or-deployed-in-care-homes-operational-guidance>.

27. Department of Health and Social Care. Adult Social Care Infection Control and Testing Fund, round 3: guidance 2021 [updated 21 October 2021. Available from: <https://www.gov.uk/government/publications/adult-social-care-infection-control-and-testing-fund-round-3>.

28. Department of Health & Social Care. Workforce Recruitment and Retention Fund for adult social care 2021 [updated 3 November 2021. Available from: <https://www.gov.uk/government/publications/workforce-recruitment-and-retention-fund-for-adult-social-care.>

29. Department of Health & Social Care. Workforce Recruitment and Retention Fund for adult social care, round 2 2021 [updated 16 December 2021. Available from: <https://www.gov.uk/government/publications/workforce-recruitment-and-retention-fund-for-adult-social-care-round-2.>

30. Department of Health & Social Care. Adult Social Care Omicron Support Fund: guidance 2022 [updated 10 January 2022. Available from: <https://www.gov.uk/government/publications/adult-social-care-omicron-support-fund/adult-social-care-omicron-support-fund-guidance.>

31. UK Health Security Agency. A guide to the spring booster for those aged 75 years and older and older residents in care homes 2022 [updated 24 March 2022. Available from: <https://www.gov.uk/government/publications/covid-19-vaccination-spring-booster-resources/a-guide-to-the-spring-booster-for-those-aged-75-years-and-older-residents-in-care-homes>.

32. Department of Health and Social Care. Infection prevention and control in adult social care: COVID-19 supplement 2022 [updated 31 March 2022. Available from: <https://www.gov.uk/government/publications/infection-prevention-and-control-in-adult-social-care-covid-19-supplement>.

33. Department of Health and Social Care. Health and wellbeing of the adult social care workforce 2022 [updated 19 May 2022. Available from: <https://www.gov.uk/government/publications/health-and-wellbeing-of-the-adult-social-care-workforce/health-and-wellbeing-of-the-adult-social-care-workforce>.
